# A local and global sensitivity analysis of a mathematical model of coagulation and platelet deposition under flow

**DOI:** 10.1371/journal.pone.0200917

**Published:** 2018-07-26

**Authors:** Kathryn G. Link, Michael T. Stobb, Jorge Di Paola, Keith B. Neeves, Aaron L. Fogelson, Suzanne S. Sindi, Karin Leiderman

**Affiliations:** 1 Department of Mathematics, University of Utah, Salt Lake City, UT, United States of America; 2 Department of Bioengineering, University of Utah, Salt Lake City, UT, United States of America; 3 Department of Applied Mathematics, University of California Merced, Merced, CA, United States of America; 4 Department of Applied Mathematics and Statistics, Colorado School of Mines, Golden, CO, United States of America; 5 Department of Pediatrics, University of Colorado School of Medicine, Aurora, CO, United States of America; 6 Department of Chemical and Biological Engineering, Colorado School of Mines, Golden, CO, United States of America; Institut d’Investigacions Biomediques de Barcelona, SPAIN

## Abstract

The hemostatic response involves blood coagulation and platelet aggregation to stop blood loss from an injured blood vessel. The complexity of these processes make it difficult to intuit the overall hemostatic response without quantitative methods. Mathematical models aim to address this challenge but are often accompanied by numerous parameters choices and thus need to be analyzed for sensitivity to such choices. Here we use local and global sensitivity analyses to study a model of coagulation and platelet deposition under flow. To relate with clinical assays, we measured the sensitivity of three specific thrombin metrics: lag time, maximum relative rate of generation, and final concentration after 20 minutes. In addition, we varied parameters of three different classes: plasma protein levels, kinetic rate constants, and platelet characteristics. In terms of an overall ranking of the model’s sensitivities, we found that the local and global methods provided similar information. Our local analysis, in agreement with previous findings, shows that varying parameters within 50-150% of baseline values, in a one-at-a-time (OAT) fashion, always leads to significant thrombin generation in 20 minutes. Our global analysis gave a different and novel result highlighting groups of parameters, still varying within the normal 50-150%, that produced little or no thrombin in 20 minutes. Variations in either plasma levels or platelet characteristics, using either OAT or simultaneous variations, always led to strong thrombin production and overall, relatively low output variance. Simultaneous variation in kinetics rate constants or in a subset of all three parameter classes led to the highest overall output variance, incorporating instances with little to no thrombin production. The global analysis revealed multiple parameter interactions in the lag time and final concentration leading to relatively high variance; high variance was also observed in the thrombin generation rate, but parameters attributed to that variance acted independently and additively.

## Introduction

When vascular injury causes blood to flow out of a vessel, the body’s response is hemostasis. Often the hemostatic response is thought of as being comprised of two stages, primary hemostasis and secondary hemostasis, though these processes begin simultaneously and are intricately intertwined. Primary hemostasis involves formation of a platelet plug which prevents the loss of blood cells and slows the outflow of plasma from the vessel. The plug forms by platelet adhesion to collagen and von Willebrand factor on the injured vessel wall and by aggregation of platelets to the wall-adherent platelets and to one another. Secondary hemostasis involves formation of a fibrin mesh in and around the platelet plug that mechanically stabilizes the plug. Blood coagulation is a key process in secondary hemostasis; it involves a network of enzyme reactions that produce the enzyme thrombin. Thrombin removes small peptide chains from the plasma protein fibrinogen thereby producing fibrin monomers. These monomers polymerize to form the fibrin mesh.

It is important to localize the hemostatic processes, in particular the production and action of thrombin, to the site of injury. Doing so is a challenge because hemostasis occurs in the presence of continued blood flow. A major part of accomplishing localization relies on the fact that the critical coagulation enzymes are enzyme complexes that form on surfaces of cells that are themselves part of or attached to the injured vessel. These include subendothelial cells and aggregated platelets. An important aspect of coagulation is that coagulation reactions are regulated by the properties of these cellular surfaces in that the formation of the enzyme complexes on them is influenced by the limited availability of binding sites and by competition among coagulation proteins to bind to these sites [1]. Additional localization mechanisms involve inhibitors in the plasma or on the endothelial cells which line intact vessels which clear active enzymes that are carried downstream of the injury by flow.

The diversity and complexity of the processes comprising hemostasis make it extremely difficult to intuit the system’s response without quantitative methods and a growing number of mathematical models have been developed to try to address this challenge. Such models can be powerful tools because they allow one to track the concentrations of every protein, enzyme complex, and cell during simulations of hemostasis. This makes them potentially very helpful in interpreting experimental data, elucidating biochemical and biophysical mechanisms, and in guiding experimental design. Recently developed models differ from one another in that they focus on different aspects of the hemostatic process and attempt to simulate events under different conditions. For example, many models of coagulation were developed as companion tools for static *in vitro* coagulation experiments using synthetic plasma with lipid vesicles providing the reaction surfaces in place of platelet surfaces [2–5] or using whole blood [6, 7]. Most models regard all species as well-mixed and only track the variation in concentrations in time. Others account for spatial and temporal variations, treating transport from one location to another to simulate the reaction-diffusion waves observed in some *in vitro* studies of coagulation in plasma [8, 9], to simulate *in vitro* flow assays with whole blood [10, 11], or to simulate small vascular injuries under flow [11–19].

Mathematical models of static situations simulate a “closed system” in which coagulation reactions occur in buffer (or plasma) in the presence of phospholipid surfaces (either lipid vesicles or platelets), and there is *no resupply* of proteins. Although the biochemical species vary from model to model because of differing assumptions on the underlying kinetic schemes, these models typically consist of a system of ordinary differential equations used to track concentrations of coagulation proteins in time starting with prescribed initial concentrations (initial conditions). The expressions that model enzymatic reactions are formed using the classical two-step reactions of enzyme kinetics in which there are association rates (*k*^+^) for binding of the substrate and enzyme, rates for the dissociation of the enzyme-substrate complex (*k*^−^), and catalytic turn over rates (*k*^*cat*^) for the rate that the complex turns over enzymes. Most of these models assume that there is a vast excess of cell (phospholipid) surface [2–5, 7] and do not track the binding sites. The first model to track limited numbers of cell binding sites [12] is the original version of the model considered in this paper. That model was further developed in [14, 16, 20, 21] and inspired similar models [4, 6]. These models account for reversible binding of proteins to these sites with rates *k*^*on*^ and rates *k*^*off*^ for binding and unbinding, respectively.

Experimentally-measured values of the Michaelis-Menten constant, *K*_*M*_ = (*k*^−^+*k*^*cat*^)/*k*^+^, and catalytic rate *k*^*cat*^ can be found in the literature for most of the coagulation reactions. However, these rate constants are often measured in isolation, i.e., considering only one reaction and the reactants involved in that reaction and are measured under varying experimental conditions, e.g., temperature, chromogenic vs. fluorogenic substrate, etc., so there is uncertainty in the parameter values. Further, it is questionable whether it is valid to use Michaelis-Menton reaction kinetics to model enzyme reactions for which either the enzyme or substrate, or both, are involved in other reactions. Hence, mass action descriptions of complex formation, dissociation, and product formation may be preferable. To parameterize such reactions, using the experimentally measured *K*_*M*_ and *k*^*cat*^ values, it is typical to choose a value for one of *k*^*on*^ or *k*^*off*^ and use the measured *k*^*cat*^ and *K*_*M*_ values to determine the other [12]. Similarly, for surface binding, published dissociation constants are ratios *K*_*d*_ = *k*^*off*^/ *k*^*on*^ of the parameters *k*^*on*^ and *k*^*off*^ needed for the mathematical model, and again it is typical to choose *k*^*off*^ and use the measured *K*_*d*_ to determine *k*^*on*^. Because of the uncertainties in experimental measurements (of *K*_*M*_, *k*^*cat*^, *K*_*d*_) and choices of kinetic schemes and rates, there is considerable variation in the inputs to mathematical models. How these uncertainties affect the models’ outputs is a major focus of this paper that we address through a systematic local and global sensitivity analysis. As we will describe in greater detail, we seek to understand how uncertainties in system inputs are propagated to uncertainties in particular model outputs taken to represent clinically relevant clotting behavior.

Mathematical models of microfluidic assays or small vessel injuries under flow simulate “open systems” in which there is a continual supply of coagulation reactants and removal of reactants and products at rates that depend on the flow rate. The supply and removal of coagulation proteins slow some reactions (e.g., by lowering the concentration of an enzyme) and speed other reactions (e.g., by maintaining a near-constant level of the substrates). Moreover, platelets provide their procoagulant surfaces to further enzyme production, but also inhibit coagulation activity on the subendothlium when they pave it over during adhesion and aggregation [12, 16, 22]. The contributions of these platelet-mediated processes should depend on the platelet count, the rate of adhesion of platelets to the subendothelium, and the numbers of binding sites on each platelet for the various coagulation proteins.

It is for precisely these nonintuitive situations that mathematical models are needed to predict system responses. Of the models mentioned above, the ones developed by our group are the most comprehensive in terms of coagulation reactions in plasma and also, perhaps even more importantly, the reactions taking place on the subendothelium and the surface of activated platelets [12, 14, 16, 20]. The sensitivity of mathematical models to *biophysical* attributes, i.e., flow (shear) rate, platelet adhesion rates, activation rates, as well as numbers of specific binding site numbers on activated platelet surfaces has never been thoroughly studied. So, in addition to analyzing the model’s sensitivity to kinetic and binding rates, investigating its sensitivity to biophysical parameters is a major goal of the current work.

In what follows, we describe the main features of the mathematical model that we analyze in this paper and then give an overview of sensitivity analysis methods and how they have been applied to other models of coagulation.

### Mathematical model of coagulation under flow

We analyze the model of Fogelson et al. [16] that simulates the clotting response due to a small injury in a blood vessel wall. Here we give an overview of the model but more details can be found in our previous works [12, 14, 16, 20]. The full list of reactions, rate constants, and model equations can be found in S1 Appendix.

**Fig 1:**
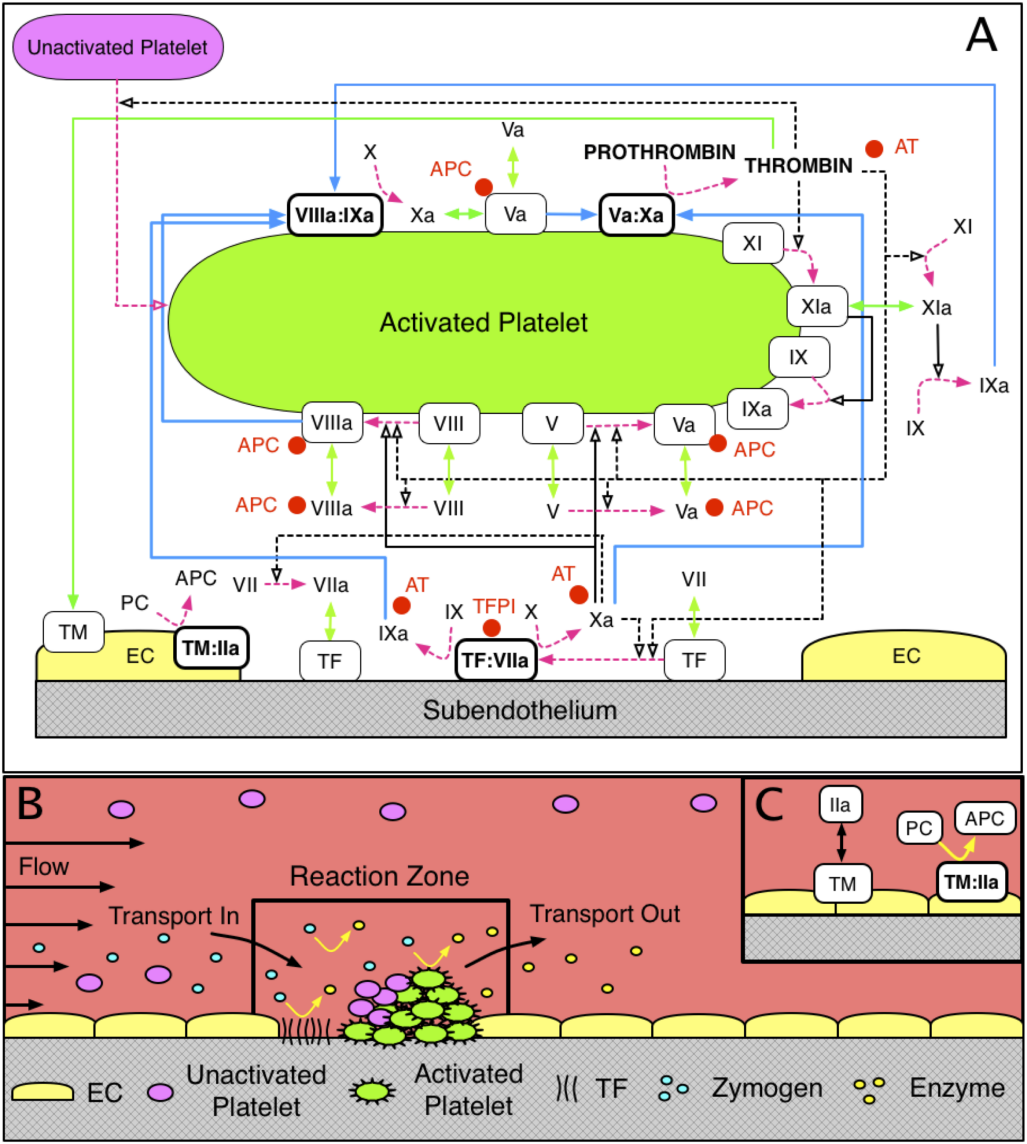
Schematic of (A) coagulation reactions included in our model. Dashed magenta arrows show cellular or chemical activation processes. Blue arrows show chemical transport in the fluid or on a surface. Green segments with two arrowheads depict binding and unbinding from a surface. Rectangular boxes denote surface-bound species. Solid black lines with open arrows show enzyme action in a forward direction, while dashed black lines with open arrows show feedback action of enzymes. Red disks show chemical inhibitors. Schematic of (B) reaction zone and (C) endothelial zone.

In the model, the clotting response is simulated within a small reaction zone located just above a small patch of exposed subendothelium (SE). Tissue factor (TF) and collagen embedded in the SE come into contact with clotting factors and platelets in the flowing plasma (see Fig 1B) to initiate the response. The initial height of the reaction zone is determined by the flow’s shear rate (the derivative of the tangential velocity component in a direction normal to the vessel wall) and platelet and protein diffusivities. Clotting factor concentrations in the reaction zone change due to their involvement in the coagulation reactions depicted in Fig 1C and also by transport in and out of the zone. Similarly, platelet concentrations change as platelets adhere to the injured wall, become activated, and as platelets are transported in and out of the zone. As platelets build up in the reaction zone the height and volume of the reaction zone increase with the volume of plasma and platelets in it changing accordingly. The concentration of each species in the reaction zone plasma is tracked with an ordinary differential equation; this choice relies on the assumption that each species is uniformly distributed (well-mixed). An additional well-mixed endothelial zone is located adjacent to the reaction zone, in the direction perpendicular to the flow (Fig 1C) with height equal to that of the reaction zone and width dependent on the flow shear rate and protein diffusion coefficients [14]. Thrombin produced in the reaction zone can diffuse from the reaction zone into the endothelial zone, bind to thrombomodulin (TM), and produce activated protein C (APC), which may then diffuse back into the reaction zone.

Platelets are assumed to be either mobile and unactivated, or stationary, activated and bound either to collagen in the SE or to other activated platelets. Platelets become activated at prescribed rates upon contact with the SE, exposure to thrombin, or contact with other activated platelets. The last of these types of activation is used as a surrogate for activation by platelet-released ADP which we do not explicitly track in this model. Protein species are assumed to be either freely moving in the fluid, bound to the SE, or bound to an activated platelet surface (APS). To move from the SE to an APS, or vice versa, a protein is subject to flow and thus might be transported out of the reaction zone.

Fig 1A shows the schematic of the coagulation reactions we consider in the model. The reactions involve many coagulation factors and cofactors: inactive enzyme precursors (zymogens), active enzymes, as well as inactive and active cofactors. Although cofactors have an active form, they do not function as enzymes, i.e., they do not activate other proteins; rather, they make the enzymes to which they bind vastly more effective than the enzymes would be alone. Next, we describe the coagulation reactions using the typical Roman numeral notation to represent the factors involved in them. For example, we refer to factor X as either ‘factor X’ or ‘fX’. Since most of the proteins have an inactive and active form, we differentiate these forms with the letter ‘a’ attached to the Roman numeral, e.g., fXa represents the active form of factor X.

In Fig 1A, the zymogens are factors VII, IX, X, and II (prothrombin) and have corresponding active enzyme factors VIIa, IXa, Xa, and IIa (thrombin), respectively. The inactive/active cofactor pairs are V/Va, and VIII/VIIIa. Also seen in Fig 1A is that many of the coagulation reactions occur only on a cellular surface, either SE or APS. We point out that there are three important surface-bound enzyme-cofactor complexes: TF:VIIa on the SE (“extrinsic tenase”) and VIIIa:IXa (“intrinsic tenase” which below we refer to simply as tenase) and Va:Xa (“prothrombinase”) on APS. Their substrates (i.e., the proteins that the enzyme complexes activate) must also be bound to the cellular surface to become activated [1].

As described above, our model incorporates specific binding sites on an APS to which each zymogen/enzyme pair compete; this is in contrast to a competing view of a surface on which all zymogen/enzyme pairs compete for one large number of shared binding sites [23]. Our assumptions are based on numerous studies in [24–29] that underscore specific binding of factors V/Va, VIII/VIIIa, IX/IXa, X/Xa, XI/XIa, respectively. Additional support for the existence of two populations of FIXa receptors comes from a series of studies, [30, 31], in which the authors characterized the numbers and binding parameters of the receptors. Other assumptions about protein interactions follow, and further discussion of them including citations to the literature can be found in [12, 14, 16]:

- Factors VII and VIIa can bind to TF in the SE. Factor Xa can activate fVII in plasma and when it is bound to TF. Factor Xa can bind the TF:VII complex directly from plasma without having to first bind the SE.
- Factors IX and X are activated by the TF:VIIa complex on the SE; they bind TF:VIIa directly from plasma. Factor X is also activated by the VIIIa:IXa (‘tenase’) complex on an APS.
- Cofactors V and VIII are activated by thrombin in plasma and by thrombin and fXa on an APS.
- Factor IX is activated by fXIa in plasma and on an APS. Factor XI is activated by thrombin in plasma and on an APS.
- The chemical inhibitors/inactivators that we include in the model are antithrombin (AT), activated protein C (APC), and tissue factor pathway inhibitor (TFPI). Since the concentration of AT is high in plasma, we assume it acts in a first-order manner to inactivate fluid-phase fIXa, fXa, fXIa, and thrombin. APC can bind to fluid-phase and platelet-bound fVa and fVIIIa to permanently inactivate them with second-order kinetics, but cannot bind to fVIIIa within a tenase complex or to fVa within a prothrombinase complex. APC is produced in the endothelial zone by a complex of thrombomodulin and thrombin. TFPI present in the plasma must first bind to fXa and then the complex TFPI:Xa must bind to the TF:VIIa complex to inhibit it.
- The activity of the TF:VIIa complex decreases as platelet deposition on the injured tissue increases, i.e., we assume that a platelet physically blocks the activity of the TF:VIIa complexes on the patch of SE to which the platelet has adhered.

Each simulation with the mathematical model requires setting numerous input values. We specify the initial plasma concentrations of platelet and protein species, the rate constants for all reactions, the numbers of specific binding sites for coagulation factors on each APS, the dimensions of the injury, the flow velocity near the injured wall, the diffusion coefficients for all fluid-phase species, and the density of exposed TF. The outputs of the model are the concentration of every protein species in the reaction zone at each instant of time from initiation of the injury until the completion of the simulation, and the concentrations of platelets attached either directly to the SE or to other platelets. A complete listing of the model’s differential equations and of the base parameter values used in the simulations can be found in S1 Appendix.

### Uncertainty and sensitivity analyses

Sensitivity analysis (SA) refers to a broad set of mathematical approaches designed to quantify how variation in model outputs may be attributed to model inputs (e.g., initial conditions and rate constants) [32–34]. These approaches allow researchers to assess how much trust to put in results obtained from a particular mathematical model. In addition, because the relevant output behavior of high-dimensional systems, such as biochemical reaction networks, is often dominated by relatively few parameters, SA provides a way to isolate these parameters so that they can be targeted by further studies.

One of the most straightforward ways to perform a SA is to vary each model input parameter one at a time (OAT) while other input parameters remain constant. Such methods typically assign importance to inputs by their impact on the approximate derivatives of outputs with respect to a change in the input [33–35]. These methods are inherently “local” in that they do not study the impact of varying parameters together. Local approaches can be informative if there is little uncertainty in model inputs or if there is little interaction between inputs (i.e., inputs act linearly or additively) [36]. However, it is unclear if this is the case in coagulation due to multiple positive and negative feedback mechanisms. Thus, in this study we employ both local and global methods to gain additional information about model sensitivity.

Global sensitivity analysis (GSA) methods consider the changes in model outputs as input parameters are allowed to vary simultaneously over specified ranges [32, 33, 35]. Typically, global methods require more computational work than local methods, but have the ability to uncover relationships between multiple parameters and they cope well with nonlinear and non-additive responses. GSA methods are often probabilistic in nature; they consider the uncertainty in model inputs and outputs as probability distributions. In these approaches the variance in model output is decomposed to attribute fractions of the variance to individual model inputs and also groups of model inputs. Many variance-based sensitivity methods consider Sobol indices [33] to express the decomposed variance as being due to variations in a single model input in isolation (first-order Sobol index), variations due to interactions involving two input parameters together (second-order Sobol index), and so on to variations due to all possible interactions, (total order Sobol index) [33]. As we describe in greater detail below, we utilize Sobol indices in our analysis and compute them through Monte-Carlo sampling. Before developing the details of our SA approach, we first describe prior SA studies of mathematical models of coagulation.

We are aware of four major SA studies of coagulation models, all of which simulate static coagulation. Danforth and colleagues [37, 38] studied sensitivity to kinetic rate constants and initial clotting factor concentrations in a model that simulates synthetic plasma in the presence of lipid vesicles (not platelets) [3, 5] with the lipids present at excess concentrations. They used an OAT, local SA that included varying individual kinetic rate constants in their first study [37]. There they examined the changes in a number of output protein species concentrations in time as kinetic rate constants were varied between 10—1000% of their reported literature value. Overall, they identified 5 of 44 kinetic rate constants that explained 50% of the variation in all model species’ outputs and 25% of the variation in thrombin output. They report many of the highest sensitivities of thrombin levels to rate constants for chemical inhibition/inactivation, i.e., by AT and TFPI, and to the reactions involving TF and VIIa. In addition, they examined how uncertainty in the output varies at a set of fixed time points, chosen to represent key moments in the process of thrombin generation. For example, they examined the time of a thrombin “burst”, described as the time that the thrombin concentration rises rapidly to physiologically relevant levels (the assume this level to be 2 nM). However, the timing of these events in their model changed significantly due to variations in the kinetic rate constants themselves, and thus it is not clear how one should interpret the sensitivities they computed at fixed time values. In a subsequent study, Danforth and colleagues studied sensitivity in the same model to variations in the initial plasma concentrations; there they analyzed sensitivity of three thrombin metrics (analogous to the ones used in the current study) to individual variations and pairwise variations of parameters within a “normal” range [38]. They reported that individual variations in two factors (TFPI and prothrombin) accounted for about 50% of the observed sensitivity of the model output. In regards to their three thrombin metrics, they reported that the pairwise changes in factor levels resulted in higher output variation than with individual variations. They found that the pairs, AT with TFPI, and AT with prothrombin, had the largest effect on the time to 2 nM thrombin and the maximum thrombin concentration. They concluded that the inhibitors TFPI and AT were the most potent inducers of overall variation. In a closed, static system, it is clear that these inhibitors play an important role in regulating thrombin generation because not only are they are the sole inhibitors in the system, but because all enzymes susceptible to inhibition in a closed system will be fully inhibited (or inactivated) in a finite amount of time. Our mathematical model with platelets and flow is less sensitive to such inhibitors since the flow and platelet coverage of the SE are often the dominant inhibitors of different parts of the process [12, 14, 16]; more discussion of these model differences will be described below.

Anand and colleagues used the SA method of Danforth to analyze sensitivity of a model of fibrin generation to kinetic rate constants [39, 40]. They reported that the fibrin concentration was most sensitive to the rate constants responsible for inactivation of FVIIIa (both intrinsic and by APC). Although their model incorporated diffusion near an injury patch in a static fluid, their sensitivity measure was reported for the average concentration throughout the spatial domain. The same group used this SA method in two follow up studies of a static model of thrombin generation that included platelets; they investigated the sensitivity of the model to rate constants [41] and to initial concentrations [42]. Thrombin generation was reported to be most sensitive to the rate constant responsible for activation of FX by tenase; however this reaction was assumed to obey Michaelis-Menton kinetics and it is unclear how this assumption affected the SA. In terms of the varied initial concentrations, they reported that thrombin generation was most sensitive to initial levels of FVIII and FVIIIa, but it is not clear why FVIIIa levels were varied in the study.

Chatterjee, Diamond, and colleagues [7] built on the models in [3, 5] and developed a different static model that accounted for platelets and platelet activation. They used this model to investigate the behavior of blood treated by corn trypsin inhibitor (CTI inhibits the intrinsic pathway via XIa). They did not consider protein binding to and unbinding from platelet surfaces, but instead assumed that reaction rates increased as a function of number of activated platelets. A brief summary of a SA was included in S1 Appendix. Similar to the approach taken by Danforth et al. [37], they conducted a local SA of 105 kinetic parameters. However, rather than a variance-based sensitivity, they considered the derivatives of system outputs at a large set of points in parameter space in an OAT fashion. Following the approach of Bentele et al. [43], their sensitivities were weighted by how closely the resulting thrombin response matched the thrombin response with the mean parameter values using a Boltzmann distribution. They then observed the most sensitive parameters were those involved with direct TF and fVIIa interactions.

Finally, Luan and colleagues [6] studied the sensitivity of a model very similar to the one used in this work, but without flow. Their model included binding/unbinding of proteins to/from specific surface binding sites, but their analysis considered only the sensitivity of kinetic rate constants (including binding rates) and not binding site numbers or other aspects of platelet function. Their goal was to identify fragile mechanisms, small perturbations to which the system output exhibited high sensitivity, and robust ones where the system maintained its performance in the face of perturbation and uncertainty. They argued that the most sensitive (or fragile) mechanisms represented ideal therapeutic targets. They conducted a local sensitivity analysis of 148 kinetic parameters, computing what they called overall state sensitivity coefficients (OSSCs) following the approach taken in [44]. Four of the top five mechanisms they identified as fragile involved factors X and Xa or the activation of platelets by thrombin. They found the mechanisms involving factors IX and IXa were moderately robust by their definitions.

In this study, we use both local and global sensitivity analysis methods to better understand what information can be gleaned from each, in the context of coagulation and platelet deposition under flow. As we explain in greater detail below, our local methods consider the direction and magnitude of change for three metrics of thrombin production. Our global sensitivity analysis methods consider the fraction of variance in these thrombin metrics that is attributable to each parameter according to computed Sobol indices.

We have divided our sensitivity analysis into four parts according to the class of parameters under study: A) sensitivity to plasma levels of zymogens and inhibitors, B) sensitivity to kinetic rate constants including those for surface binding/unbinding, C) sensitivity to biophysical and platelet attributes and, D) sensitivity to all classes of parameters considered together. In parts A and C we found the local and global analyses produced consistent results in terms of ranking the overall sensitivity of the thrombin metrics to the parameters in those classes. This suggests that the dominant impacts of input uncertainty in these restricted classes may be understood through consideration of each parameter in isolation. However, in parts B and D, we observed a number of instances where varying parameters together resulted in much greater variance in model output than varying them in isolation. While the variance attributable to higher-order effects was not a majority of the total system variance in these cases, the higher-order effects were particularly pronounced for two of our three output metrics when all parameters were varied together (part D). Overall, our results provide compelling evidence that to fully characterize the behavior of coagulation, the joint impact of multiple parameters should be considered. Beyond measuring the amount of variance in the output, as we illustrate in our analysis, global methods also proved useful for identifying groupings of parameters associated with extreme model output behavior, which is notable since the parameters varied within normal physiological ranges.

## Materials and methods

Here we describe the methods used to analyze the sensitivity of our model’s output. We focus on the model’s production of thrombin and quantify this in terms of three metrics, similar to those used in the *in vitro* experiments from our previous study [45]:

1. **Lag time:** A measure of how fast the system is turned on. Specifically, we quantify the amount of time it takes for thrombin to reach 1 nM, denoted by *t*_1*nM*_. This concentration of thrombin is known to have significant effects on platelet activation and coagulation [46].
2. **Maximum relative rate:** A measure of how fast thrombin is produced once the system is turned on. Specifically, it is defined by
  $$\begin{array}{c} \max_{t>t_{1nM}}\{\frac{d[\text{thrombin}]}{dt}/\left[\text{thrombin}\right]\}, \end{array}$$
  where *t*_1*nM*_ is the time it takes for thrombin concentration to reach 1.0 nM.
3. **Final concentration:** The total thrombin concentration after 20 minutes.

**Fig 2:**
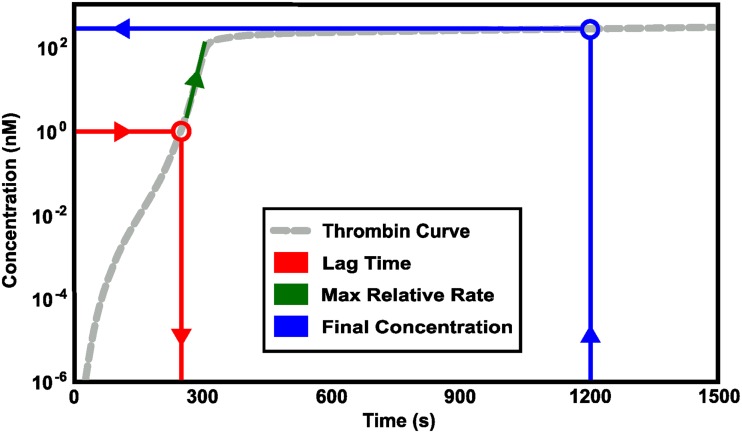
Schematic of thrombin metrics. Three physiologically relevant metrics of Thrombin generation were calculated for sensitivity analysis: 1) lag time (*Red*), 2) maximum relative rate of thrombin generation, measured after 0.1nM of thrombin have been obtained (*Green*), and 3) final concentration, the total concentration of thrombin at 20 minutes (*Blue*).

Fig 2 illustrates how these metrics relate to a curve of thrombin concentration vs. time. In all comparisons that follow, we assess the variation and sensitivity of thrombin generation according to these three metrics.

Next, we detail the mathematical and statistical methods used in our local and global sensitivity analyses. We examined the sensitivity of the model’s output to three types of parameter variations: (i) the plasma levels of seven zymogens and two inhibitors, (ii) the values of 96 kinetic rate constants, and (iii) the platelet number density (“platelet count”), the numbers per activated platelet of nine types of platelet surface binding sites, and the rates of six platelet responses. We also studied the effect of the flow’s shear rate on model outputs. We conducted local and global sensitivity studies in parallel for the plasma levels and platelet characteristics. For the 96-dimensional parameter space of kinetic rate constants, a full global analysis would have been too computationally costly. For these parameters, we used the Morris Method [47] as a screening method to determine a subset that seemed likely to have a strong influence on the thrombin concentration, either through direct effects by themselves or through interactions with other parameters. This subset was then analyzed with our global methods. A similar process was carried out on the union of the three classes of parameters to generate a subset that was then used for global analysis.

### Local sensitivity analysis

The local approach we use is a variant of the one-at-a-time (OAT) method used by Saltelli et al. [34, 48]. Our initial approach to local sensitivity was to pursue a derivative based method to quantify the sensitivity of each metric with respect to changes in the parameter. We used a centered difference to approximate the derivative with respect to each parameter OAT at a range of parameter values (50%, 75%, 100%, 125%, and 150% of the baseline values.) However, we observed some unexpected behavior with respect to the system’s metrics which caused us to develop an alternative approach to local sensitivity.

**Fig 3:**
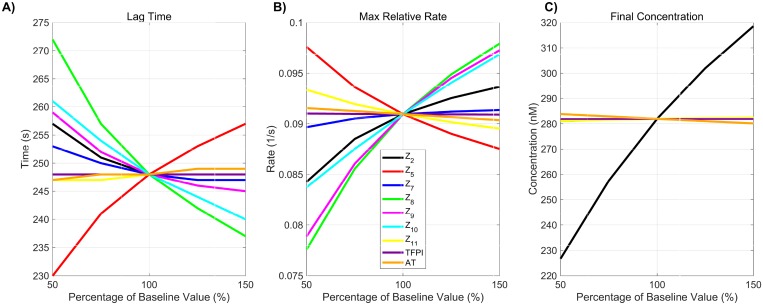
Monotonicity of change in thrombin generation due to variation in plasma levels. Variation in the three physiologically relevant metrics of thrombin generation: 1) lag time; 2) maximum relative rate; and 3) final concentration that occur with variations in plasma levels.

Despite the nonlinearity of the model equations, and the multiple positive and negative feedback loops in the coagulation system, we found that each of the metrics we were interested in behaved monotonically with respect to varying each plasma level and kinetic rate constant OAT from 50% to 150% of the standard values. For example, in Fig 3 we show how each of our three metrics varies with plasma levels and note that, as each level ranges from 50% to 150% of its standard value, the metric is either monotonically increasing or decreasing. The monotonicity property was also seen for nearly all instances of OAT variation in platelet characteristics (see S1 and S2 Figs). The only exception was non-monotonic variation in the lag time with the platelet count *PL*^*UP*^ and adhesion rate *k*_adh_. Because of this surprising behavior of system outputs, we quantified parameters by the absolute difference they produced in each metric when considering their extremal values (i.e., 50% and 150%). For each metric, we ranked the parameters by considering their relative absolute difference.

More specifically, let **x** = (*x*_1_, *x*_2_, …, *x*_*P*_) be the standard model parameter values and *m*_*i*_(**x**_*j*,*y*%_) represent the value of the *i*-th metric when parameter *j* is chosen to be *y*% of its standard value and all other parameters are chosen to be their standard value. We define the local sensitivity of the *i*-th metric to the *j*-th parameter as:

$$\begin{array}{c} LS_{j}^{i}=\frac{|m_{i}\left(x_{j,150\%}\right)-m_{i}\left(x_{j,50\%}\right)|}{\max_{k}\left(\right|m_{i}\left(x_{k,150\%}\right)-m_{i}\left(x_{k,50\%}\right)\left|\right)}. \end{array}$$ (1)

**Fig 4:**
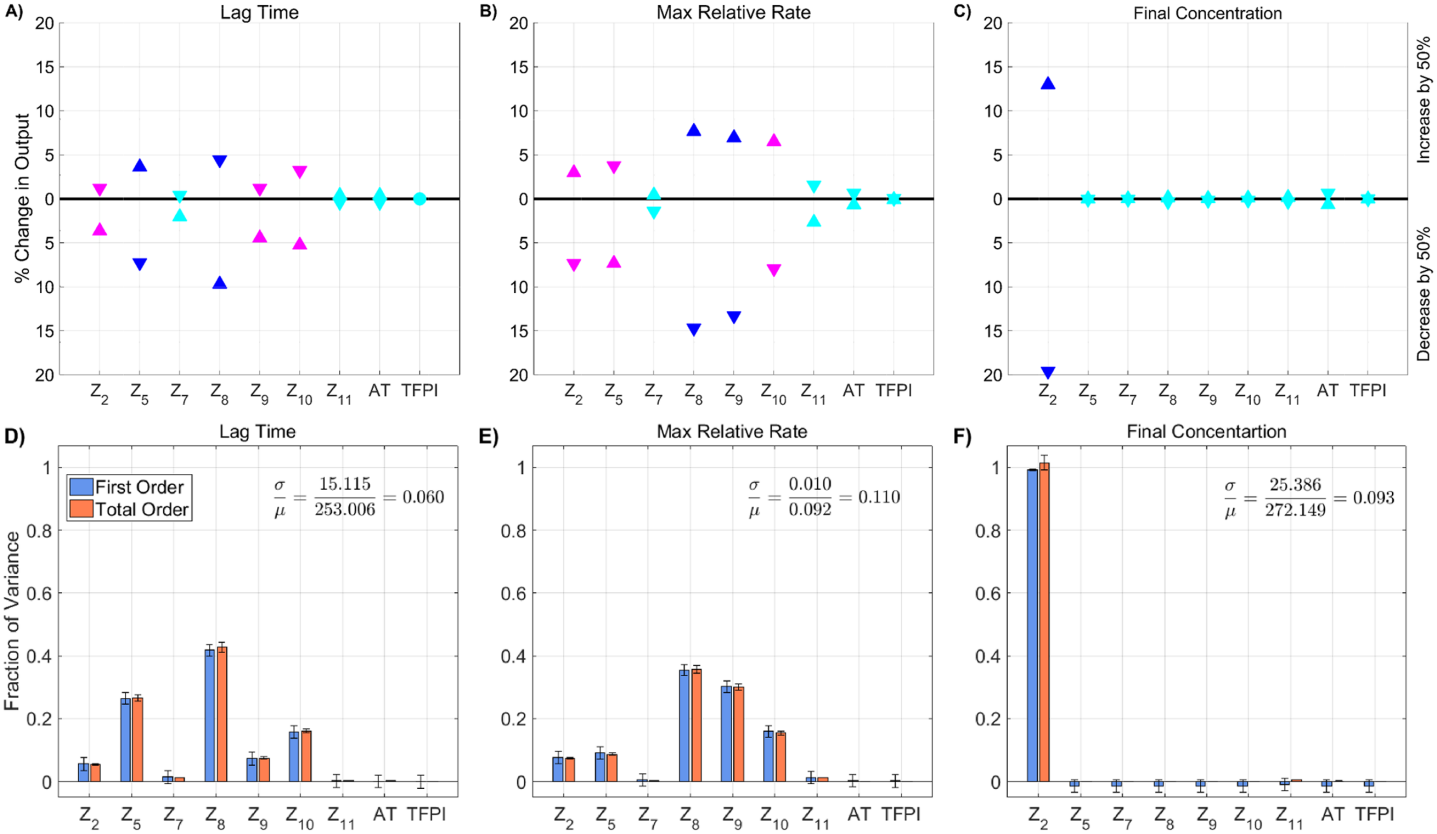
Sensitivity of total thrombin generation to plasma levels. Variation in the **A,D)** lag time; **B,E)** maximum relative rate; **C,F)** final concentration; to zymogen and chemical inhibitor levels using the local (OAT) method (**A-C**) and global Sobol method (**D-F**). *Local*: Sensitivities $LS_{j}^{i}$ that lie between 0.75 and 1 (blue), between 0.25 and 0.75 (magenta), less than 0.25 (cyan) determine the rank-ordered list of initial levels. The percent change of thrombin generation measures from baseline model output for each initial condition are represented by triangles. The direction of variation of the input parameter is indicated with an upwards or downwards facing triangle. *Global*: First and Total Order Sobol indices are plotted as bars with errors of 2 standard deviation about the mean, computed with 5,000 bootstrap samples of the original 110,000 function evaluations. The coefficient of variation is included to provide a scale for the fraction of variance. No total order index was statistically significantly larger than the first order index, indicating that the model output is not significantly effected by interactions between the parameters considered here.

Each $LS_{j}^{i}$ is a number between 0 and 1 and we use these values to rank input sensitivities. In our local sensitivity results (e.g., top of Fig 4), we color results in blue when $LS_{j}^{i}\in \left(0.75,1\right]$, magenta when $LS_{j}^{i}\in \left(0.25,0.75\right]$ and cyan otherwise. In addition, because the response of the system outputs is monotonic throughout the entire range, we show separately the change in each metric for the 50% increase and decrease for each parameter indicating with a up/down triangle if the metric increases or decreases.

### Morris method

Because a full global analysis of all model parameters or of the 96 kinetic rate constant subset was computationally expensive, we sought to select subsets of parameters that seemed likely to have strong effects. To determine which model parameters may be considered to have effects which are negligible, linear or involved in interactions with each other, we implemented the method of Morris [47]. Here, we briefly detail this method and discuss our basis for selecting which parameters were used in our global sensitivity results.

The Morris method involves individually randomized OAT experiments and the calculation of two sensitivity measures μ*, the Morris mean, and *σ*, the standard deviation. The lower and upper bounds of the interval of sampling were set to 50% and 150% of the standard parameter values. The Morris design requires a random selection of a “base” sample point x*, which is a vector of length equal to the number *P* of parameters. Using five Morris levels (*p* = 5), each parameter value is increased or decreased by 25% of its standard value (Δ = 1/(*p* − 1) = 1/4). A sequence of *P* + 1 sample points x*, **x**^(1)^, **x**^(2)^, …, **x**^(*P*)^, called a trajectory, is generated with the property that two consecutive points differ only one parameter’s value. For this analysis, we generated 1000 random trajectories and selected *r* = 25 samples in a way to maximize their “spread” in the input space. The spread is based on the sum of the geometric distances between coupled points of any two fixed trajectories. More details regarding sample selection are found in [49].

An elementary effect for each parameter on each metric was calculated from the sample trajectories. For example, suppose that in the *k*^*th*^ trajectory a step in the *j*^th^ model parameter occurs between sample points $x_{k}^{\left(\ell_{j}\right)}$ and $x_{k}^{\left(\ell_{j}+1\right)}$. For a given choice of parameters, **x**, let *m*_*i*_(**x**) be the value of the *i*^th^ metric of thrombin generation. The elementary effect for model parameter *j* and trajectory *k* associated with the thrombin generation metric *i* is:

$$\begin{array}{c} d_{j,k}^{i}=\frac{1}{\Delta }|m_{i}(x_{k}^{\left(\ell_{j}\right)})-m_{i}(x_{k}^{\left(\ell_{j}+1\right)})|. \end{array}$$ (2)

The Morris mean and standard deviation for the *j*^*th*^ parameter and the *i*^th^ thrombin generation metric are defined as the empirical mean and variance over all trajectories:

$$\begin{array}{c} \mu_{i,j}^{*}=\frac{1}{r}\sum_{k=1}^{r}d_{j,k}^{i}\;\;\;\;\;\text{and}\;\;\;\;\;\sigma_{i,j}=\sqrt{\frac{1}{\left(r-1\right)}\sum_{k=1}^{r}{\left(d_{j,k}^{i}-\mu_{i,j}^{*}\right)}^{2}}, \end{array}$$

where *r* is the number of trajectories. Thus the method produces a coordinate pair, $\left(\mu_{i,j}^{*},\sigma_{i,j}\right)$, for each thrombin generation metric and each parameter. Typically, the coordinate pairs $\left\{\left(\mu_{i,j}^{*},\sigma_{i,j}\right)\right\}$ are grouped into three sets according to those that have a negligible effect on the metric (both $\mu_{i,j}^{*}$, *σ*_*i*,*j*_ small), a linear effect on the metric ($\mu_{i,j}^{*}>\sigma_{i,j}$ with *σ* small) and those likely to have interaction effects ($\mu_{i,j}^{*}<\sigma_{i,j}$ with both $\mu_{i,j}^{*}$, *σ*_*i*,*j*_ large).

When determining which parameters to select for global analysis, we took a different approach. First, we performed the procedure detailed above, with parameter space filling trajectory selection, three times to generate path sets *P*_1_,*P*_2_, and *P*_3_. Because results selected by this method are path-dependent, these multiple path sets allowed to mitigate the risk of missing important parameters. Next, we normalized all $\mu_{i,j}^{*}$ and *σ*_*i*,*j*_ by dividing each by the largest values observed for that metric:

$$\begin{array}{c} \mu_{i,\text{max}}^{*}=\max_{j}\left\{\mu_{i,j}^{*}\right\}\;\text{and}\;\sigma_{i,\text{max}}=\max_{j}\left\{\sigma_{i,j}\right\}. \end{array}$$

This normalization restricts all coordinate pairs to the unit square. Inputs with an *ℓ*_2_-norm less than 0.5 are deemed to have negligible effects on thrombin generation, whereas parameters with an *ℓ*_2_-norm greater than or equal to 0.5 were viewed as potentially able to induce significant change in thrombin generation (individually or via interactions). We selected all parameters whose normalized coordinate pair was at a distance ≥ 0.5 from the origin for the lag time, maximum relative rate, *or* final concentration for *any* of the path sets (*P*_1_, *P*_2_, and *P*_3_).

### Global sensitivity analysis

Global sensitivity analysis considers the impact of varying parameters *simultaneously* and uniformly over their full range of possible values, here values between 50% and 150% of baseline. As such, we consider the underlying system output to be a random variable over a probability space of parameter inputs, and quantify the sensitivity of a model output by its variance. We estimate the effects of parameter variations by using Monte Carlo sampling to explore our parameter space. While computationally taxing, Monte Carlo sampling is easily implemented and applicable to all models, including those that contain non-linear interactions among model parameters [33].

We quantify the variance of model outputs by Sobol sensitivity indices (SIs) [48, 50]. Assuming that parameters are independent, the variance of the *i*-th model metric, *m*_*i*_, with respect to random parameter inputs **x** = (*x*_1_, *x*_2_, …, *x*_*P*_), may be decomposed as:

$$\begin{array}{c} \text{Var}[m_{i}\left(x\right)]=V^{i}=\sum_{j=1}^{P}V_{j}^{i}+\sum_{j=1}^{P}\sum_{k=j+1}^{P}V_{jk}^{i}+\cdots +V_{1\ldots P}^{i}. \end{array}$$ (3)

The total variance is a sum of first order effects of the *j*-th parameter on metric *i*, $V_{j}^{i}$, as well as higher order effects from interactions of parameters. (For example, $V_{jk}^{i}$ is the contribution to the variance of metric *i* from *j*-th and *k*-th parameters.) The first order SIs are formed by normalizing the first order variance terms by the total variance

$$\begin{array}{c} GS_{j}^{i}=\frac{V_{j}^{i}}{V^{i}}, \end{array}$$ (4)

where $\sum GS_{j}^{i}=1$ if and only if all interaction terms have zero contribution to the variance of the model metric *m*_*i*_. The total order SI, $GS_{j}^{i,Tot}$, captures the effect on *V*^*i*^ of parameter *j*, including those from interaction terms, by summing all variance terms where the *j*-th parameter appears, typically written as

$$\begin{array}{c} GS_{j}^{i,Tot}=\frac{V^{i}-V_{-j}^{i}}{V^{i}}, \end{array}$$ (5)

where $V_{-j}^{i}$ is the sum over the set of all variance terms *not* containing the *j*-th parameter. Unlike the first order SIs, the total order SIs can sum to greater than one as all of the interaction terms between parameters appear in each parameter’s total SI.

Several estimators for the partial variances are found in the literature [48], with varying accuracy and efficiency. We have elected to use the first order variance estimator [50], defined as

$$\begin{array}{c} V_{j}^{i}\approx \frac{1}{N}\sum_{k=1}^{N}m_{i}\;(x^{\left(k\right)})\;m_{i}\;(x_{j}^{\left(k\right)})-E\left[m_{i}\right], \end{array}$$ (6)

where **x**^(*k*)^ and $x_{j}^{\left(k\right)}$ are each a sample of all model parameters and differ only in the *j*-th parameter’s value. While true variances are never negative, this estimator may produce negative estimates, but it generally produces estimates with a low absolute error compared to other methods [48].

Similarly, the total variance due to the *j*-th parameter, (i.e. the sum of all variance terms containing the *j*-th parameter) is estimated using the following estimator (from [51]), with

$$\begin{array}{c} V^{i}-V_{-j}^{i}\approx \frac{1}{2N}\sum_{k=1}^{N}{\left[m_{i}\;\left(x^{\left(k\right)}\right)-m_{i}\;\left(x_{-j}^{\left(k\right)}\right)\right]}^{2}, \end{array}$$ (7)

where again **x**^(*k*)^ and $x_{-j}^{\left(k\right)}$ are sample points in the parameter space of the model parameters, however these differ in all parameter values *except* that of the *j*-th parameter, for which they share the same value. Due to this estimator’s non-negative nature, it is extraordinarily efficient, producing estimates with extremely low error, especially compared with the first order variance estimator in 6. So while the true first order variance is less than or equal to the total variance, the noise in the computed estimates can lead to violations of this relationship.

The total number of function evaluations required are *N*(*P* + 2), where *N* is the number of samples for each individual parameter and *P* is the number of parameters. Unless otherwise stated, we used *N* = 10,000. Confidence intervals for the SI estimates were generated using a bootstrap approach [52]. Unless otherwise specified, confidence intervals were computed based on 5,000 resamples.

### Robustness to shear rate

The shear rate we use in the sensitivity analyses of this paper is fixed at 100 s^−1^. Previous work [12, 14, 16, 45] includes the investigation of coagulation and platelet deposition dynamics in an environment with this shear rate and other shear rates and shows that large variations in shear rate affects thrombin generation. This raises the question of the robustness of the sensitivity analyses to small perturbations in shear rate. Due to computational limitations, we did not test robustness of the global Sobol method to shear rate. We did, however, explore the effects of small perturbations in shear rate on the Morris method screening. The method of Morris with the same path set was conducted three times using three shear rates, 90, 100, 110 (1/s). Results from this experiment represent the effects of a 10% change in shear rate (see S3 Fig) and give the associated *ℓ*_2_-norm of the Morris sensitivity measures μ*, *σ* for lag time*, maximum relative rate*, and final amount. Parameters with the normalized *ℓ*_2_-norm of the Morris mean μ* and standard deviation *σ* greater than 0.5 for any of the path subsets were chosen for comparison across shear rates. The Morris sensitivity norms for all three thrombin generation metrics are essentially the same across all three shear rates. These results highlight that the method of Morris is not sensitive to small perturbations in shear rate. We proceed with confidence in our analyses at a fixed shear rate of 100 s^−1^.

## Results

### Varying plasma levels

Plasma levels of procoagulant and anticoagulant species (e.g., zymogens and chemical inhibitors) naturally vary between 50% and 150% of their baseline physiological value [53]. In this section we explore how thrombin generation is affected by variations within this normal range of zymogen and chemical inhibitor levels. We use a local SA approach in which thrombin generation is simulated using three samples from each zymogen and chemical inhibitor level, 50%, 100%, and 150% of normal. We report only these three cases due to the monotonicity in the model responses to changes in plasma levels (see Methods section). A global SA was also used in which each zymogen and chemical inhibitor were simultaneously sampled from a uniform distribution between 50% and 150% of normal, resulting in a total of 110,000 distinct evaluations of the model. Fig 4A–4C show results from the local SA and display the percent changes in each thrombin metric and identify the directionality of the sensitivity for each measure of generation, with upward/downward facing triangles indicating a 50% increase/decrease in the input from baseline. The color of the triangle indicates overall sensitivity, $LS_{j}^{i}$, where highest to lowest sensitivity is represented by the ordering blue, magenta, and cyan. Fig 4D–4F display results from the global SA and show the first and total order Sobol indices computed for each initial condition and metric. The height of the bars indicate the fraction of variance that is attributable to the model output from each parameter individually (first order) and from the parameter itself and its interactions with other parameters (total order).

The local SA results in Fig 4A–4C reveal the most influential zymogen concentrations when perturbed one at a time for each thrombin metric. We found that variations in fVIII (*Z*_8_) and fV (*Z*_5_) levels have the greatest effect on the lag time. An increase/decrease of fVIII and fV levels by 50% leads to approximately a 10% change in lag time from baseline. This is equivalent to an increase/decrease of 25 seconds. Both chemical inhibitor levels, TFPI and AT, have little effect on the lag time (Fig 4A). Variations in fVIII and fIX (*Z*_9_) levels, which influence the rate of formation of the tenase complex on platelets, have the largest effect on the maximum relative rate of thrombin generation (Fig 4B). Increasing fVIII or fIX levels by 50% leads to less than a 10% increase in this rate. A stronger effect is seen when both zymogens are (individually) decreased by 50%. The maximum relative rate decreases by 14% and 13%, when fVIII and fIX levels are decreased. Lastly, prothrombin (*Z*_2_) levels most dramatically affect the final concentration (see Fig 4C). A 50% decrease in prothrombin level results in a 20% decrease in thrombin levels (55 nM), making it the largest OAT effect on thrombin generation produced by varying plasma levels.

The global SA results are in agreement with the findings of the local analysis in that the ranking of the most influential parameters are nearly identical for the two methods on all three metrics (*p* < 0.001 from a rank permutation test [54]). Varying all zymogen and chemical inhibitor levels between 50% and 150% of normal simultaneously resulted in a coefficient of variation in the lag time of 0.06 (Fig 4D), indicating that the lag time is robust to changes in plasma levels within the normal range. As in the local SA, fVIII and fV play the largest role in changes to the lag time, accounting for 26% and 42% of the output variance, respectively. The maximum relative rate of thrombin generation (Fig 4E) had the greatest coefficient of variation across all three metrics at 0.11. The global SA shows that fVIII, fIX, and fX are the main contributors to the model variance, at 36%, 30%, and 16% respectively, as in the case of the local SA. The variation in the final concentration (Fig 4F) is entirely explained by variation in the plasma prothrombin levels. For all three metrics, the output variance is dominated by the first-order effects of parameters. No total-order Sobol index statistically exceeds the first-order index (*p* < 0.05). This implies that the model is additive in this regime and is not strongly impacted by interactions between parameters.

**Fig 5:**
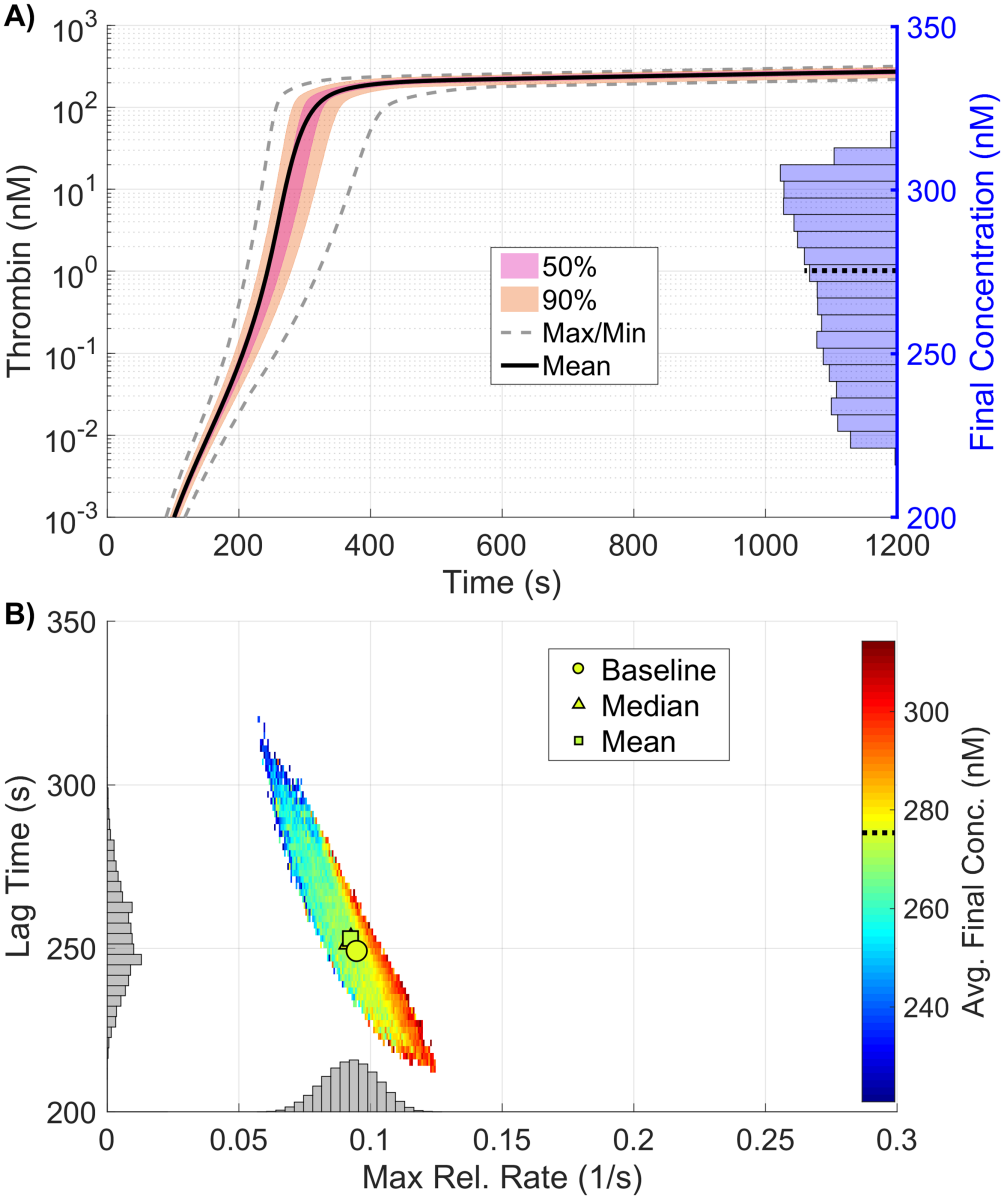
Variation in thrombin generation as a result of varying plasma levels. **A)** *Left Axis:* Thrombin concentration time series showing the mean (solid black line) and boundaries that encompass 50% of the data (pink), 90% of the data (orange), and the maximum/minimum of the computed solutions (gray-dashed) generated by uniformly varying initial zymogen plasma levels from 50-150% of normal simultaneously (110,000 total function evaluations). *Right Axis:* Marginal histogram of final thrombin concentration at *t* = 1200 seconds. **B)** Heatmap and marginal histograms relating three important thrombin generation metrics: lag time (*y-axis*), maximum relative rate (*x-axis*), and final concentration (*color-axis*). Results were obtained by post-processing samples used to compute the global sensitivity indices. Dashed black bar in (A) and (B) represents the baseline case of 275nM of thrombin at 20 minutes.

The samples used in the global SA not only allow us to probe the sensitivity of the model parameters, but also to interrogate the resulting distribution of thrombin generation metrics. Fig 5 shows the resulting output from the 110,000 samples of the coagulation model used to compute the Sobol indices above in which plasma levels varied independently between 50% and 150%. Fig 5A shows a quantile plot in time of the thrombin curves generated from the samples, with the max/min corresponding to the empirical support (note, the lines on the graph do *not* refer to actual thrombin curves but instead to computed quantiles). The individual distributions of the three thrombin generation metrics resulting from the sampling are shown as histograms (final concentration in A, with lag time (*y-axis*) and the maximum relative rate (*x-axis*) in B), along with their joint distribution, shown as a colored 2D histogram in B. The dependent relationship between the three metrics is clearly seen, as lag time decreases almost linearly with the maximum relative rate, while the average final concentration increases nearly linearly in the same range. While moderate variations in each metric can be seen, the mean and median of the full collection is within 3% of a baseline reference thrombin curve for all metrics, implying that though variations do exist, the typical response is always consistent with that of the baseline case.

### Varying kinetic rate constants

In this section we explore how variations in the values of kinetic rate constants (KRCs) affect thrombin generation. First, we use our local SA approach and vary the KRC values to 50% and 150% of their baseline values. In addition to a local SA method, we performed a screening using the method of Morris as a precursor to a global SA. The screening selected parameters that either had large individual effects upon the model output or a high likelihood of interacting with other parameters. With this screening, we determined a subset of 25 parameters (out of 96) to include in our global SA.

#### Local sensitivity analysis

We performed a local SA by varying each of the 96 KRCs individually and report the 25 most sensitive parameters in S4 Fig. The greatest effect on lag time (an increase of 27% or 1 min) occurred with a 50% decrease in the rate of activation of fX by TF:VIIa $k_{z_{10}^{m}:e_{7}^{m}}^{cat}$. The greatest effects on the maximum relative rate of thrombin generation occurred for variations in nine KRCs; a 50% change in any one of these altered this rate by about 20%. The nine KRCs are the rate $k_{8}^{\text{off}}$ of fVIII/fVIIIa unbinding from its platelet surface binding sites, the rates of activation of prothrombin by prothrombinase $k_{z_{2}^{m}:PRO}^{\text{cat}}$, of fX by tenase $k_{z_{10}^{m}:TEN}^{\text{cat}}$, and of fIX by TF:VIIa $k_{z_{9}:e_{7}^{m}}^{\text{cat}}$, the Michaelis-Menten constant for activation of TF:VII by fXa $K_{z_{7}^{m}:e_{10}}^{\text{M}}$, the rates of fIX/fIXa binding to and unbinding from receptors on platelet surfaces, $k_{9}^{\text{on}}$ and $k_{9}^{\text{off}}$, and of thrombin binding to the platelet surface $k_{2}^{*,\text{on}}$. The most dramatic effects on the final thrombin concentration occurred with 50% decreases in the rate of prothrombin binding to platelets $k_{2}^{\text{on}}$ and the rate of activation of prothrombin to thrombin $k_{z_{2}^{m}:PRO}^{\text{cat}}$. These changes caused an 18% and 15% reduction in the final thrombin concentration, respectively. The largest effect on any of the metrics was that which occurred with a 50% reduction in the rate of activation of fX by TF:VIIa.

The local SA reveals that overall the ten most sensitive KRCs, when perturbed one at a time to 50% or 150% of their baseline values, are $k_{z_{9}:e_{7}^{m}}^{\text{cat}}$, $k_{z_{10}:e_{7}^{m}}^{\text{cat}}$, and $K_{z_{7}^{m}:e_{10}}^{\text{M}}$, which are involved in activation of fIX and fX by TF:VIIa on the subendothelium; $k_{9}^{\text{on}}$, $k_{9}^{\text{off}}$, and $k_{8}^{\text{off}}$ which influence the rate of formation of tenase on the platelet surfaces; $k_{z_{10}^{m}:TEN}^{\text{cat}}$, which is the rate of activation of fX by tenase; $k_{2}^{\text{on}}$ and $k_{z_{2}^{m}:PRO}^{\text{cat}}$ which directly affect thrombin production by prothrombinase; and $k_{2}^{*,\text{on}}$ which affects the ability of thrombin to feedback and activate the cofactors fVIIIa and fVa of tenase and prothrombinase, respectively. This analysis identifies parameters that are potential candidates for the global SA, but selection of this subset is not informed by interaction effects. This motivates our use of the method of Morris to do additional screening. We note that the maximum percent change in output for any of these KRCs, varied one at a time, is less than 30% and that many of them induced changes of less than 10%.

#### Method of Morris

The method of Morris procedure was applied to 96 kinetic rate constants with three path sets *P*_1_, *P*_2_, and *P*_3_. S5 Fig highlights the KRCs screened as candidates for the global Sobol method. Selection of parameters was based on the criteria that the normalized *ℓ*_2_-norm of the Morris mean μ* and standard deviation *σ* be ≥ 0.5 for any of the path subsets and any of the thrombin generation metrics. The selected subset of 25 KRCs includes activation rates of zymogens by enzymes, binding/unbinding rates on a platelet surface and formation/dissociation rates of complexes. Kinetic rates with *ℓ*_2_-norms ≥ 0.5 in *all three* thrombin generation metrics include binding rates of fVIII/fVIIIa, fIX/fIXa, and thrombin to their respective binding sites on an activated platelet surface ($k_{8}^{on}$, $k_{9}^{on}$, $k_{2}^{on,*}$), activation rates of fX by tenase and prothrombin by prothrombinase ($k_{z_{10}^{m}:TEN}^{\text{cat}}$, $k_{z_{2}^{m}:PRO}^{\text{cat}}$), and the formation rate of tenase ($k_{e_{8}^{m}:e_{9}^{m}}^{+}$).

The activation rates of fIX and fX by TF:VIIa, the unbinding rates of fV/fVa, fVIII/fVIIIa, fIX/fIXa, and fX/fXa from a platelet surface, the activation rates of fV by thrombin and fXa, and the formation rate of prothrombinase on a platelet surface have significant Morris sensitivity norms associated with lag time. All parameters selected except the binding rate of prothrombin and the unbinding rates of thrombin, fV/fVa, and fX/fXa on the platelet surface have significant Morris *ℓ*_2_-norms associated with the maximum relative rate of thrombin generation. Lastly, the binding rate of prothrombin, the unbinding rate of thrombin and the formation rate of prothrombinase all have significant *ℓ*_2_-norms associated with the final concentration of thrombin. The final list of 25 kinetic rate constants all correspond to the formation of tenase and prothrombinase on a platelet surface and the activation of thrombin.

#### Global sensitivity analysis

**Fig 6:**
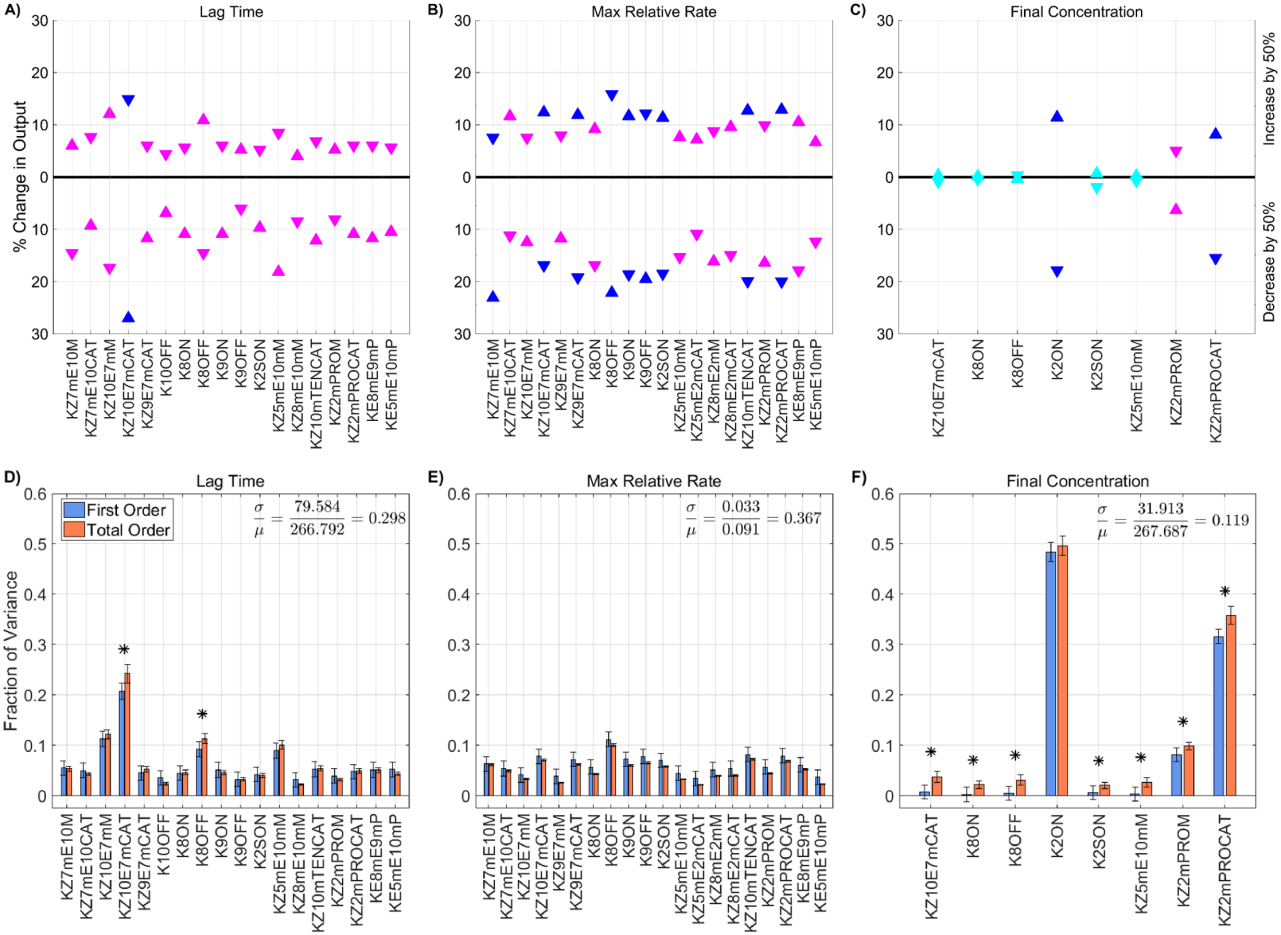
Sensitivity of thrombin generation to KRCs. Variation in the **A,D)** lag time; **B,E)** maximum relative rate; **C,F)** final concentration; to platelet characteristics using the local (OAT) method (**A-C**) and global Sobol method (**D-F**). *Local*: Sensitivities $LS_{j}^{i}$ that lie between 0.75 and 1 (blue), between 0.25 and 0.75 (magenta), less than 0.25 (cyan) determine the rank-ordered list of kinetic rate constants. The percent change of thrombin generation measures from standard model output for each initial condition are represented by triangles. The direction of variation is indicated with an upwards or downwards facing triangle. *Global*: First and Total Order Sobol indices are plotted as bars with errors of 2 standard deviation about the mean, computed with 5,000 bootstrap samples of the original 540,000 function evaluations. The coefficient of variation is included to provide a scale for the fraction of variance. PCs with Total Order index statistically significantly larger than the First order index are indicated with a star.

Fig 6 shows the global SA for the 25 KRCs obtained by the Morris screening. The KRCs were uniformly sampled, simultaneously, from 50% to 150% of their baseline values. Note, only parameters with a non-trivial total order index are displayed (*p* < 0.05). While not shown here, the local SA results for the omitted parameters agree well with the global SA results in that they were all approximately zero. The coefficients of variation for all metrics range between 0.12 for the final concentration and 0.37 for the maximum relative rate and are significantly greater than those observed when varying plasma zymogen and inhibition levels, indicating that the model output is more strongly dependent on the values of the KRCs, at least when they are varied *simultaneously* and uniformly. The global Sobol indices agree with the local SA results, yielding the same relative ranking of parameter importance for the tested variables in all metrics (*p* < 0.05 from a rank permutation test [54]). The global results show that several parameters have non-trivial interaction effects (i.e., total order effect is statistically larger than the first order effect with *p* < 0.05), which we have indicated with an asterisk. For lag time, both $k_{z_{10}^{m}:e_{7}^{m}}^{\text{cat}}$ and $k_{8}^{\text{off}}$ have statistically significant interaction effects (*p* < 0.05), which account for approximately 3% and 2% of the total output variance, respectively. While the final concentration output is dominated by the two first order effects of $k_{2}^{\text{on}}$, the binding rate of fII to the platelet surface, and $k_{z_{2}^{m}:PRO}^{\text{cat}}$, the activation of fII by prothrombinase, numerous significant interaction effects also are present, most of which have no significant first order effect at all (i.e., the first order term’s confidence intervals contain zero). These interaction effects are potentially important, accounting for up to 10% of the variance in the final concentration.

**Fig 7:**
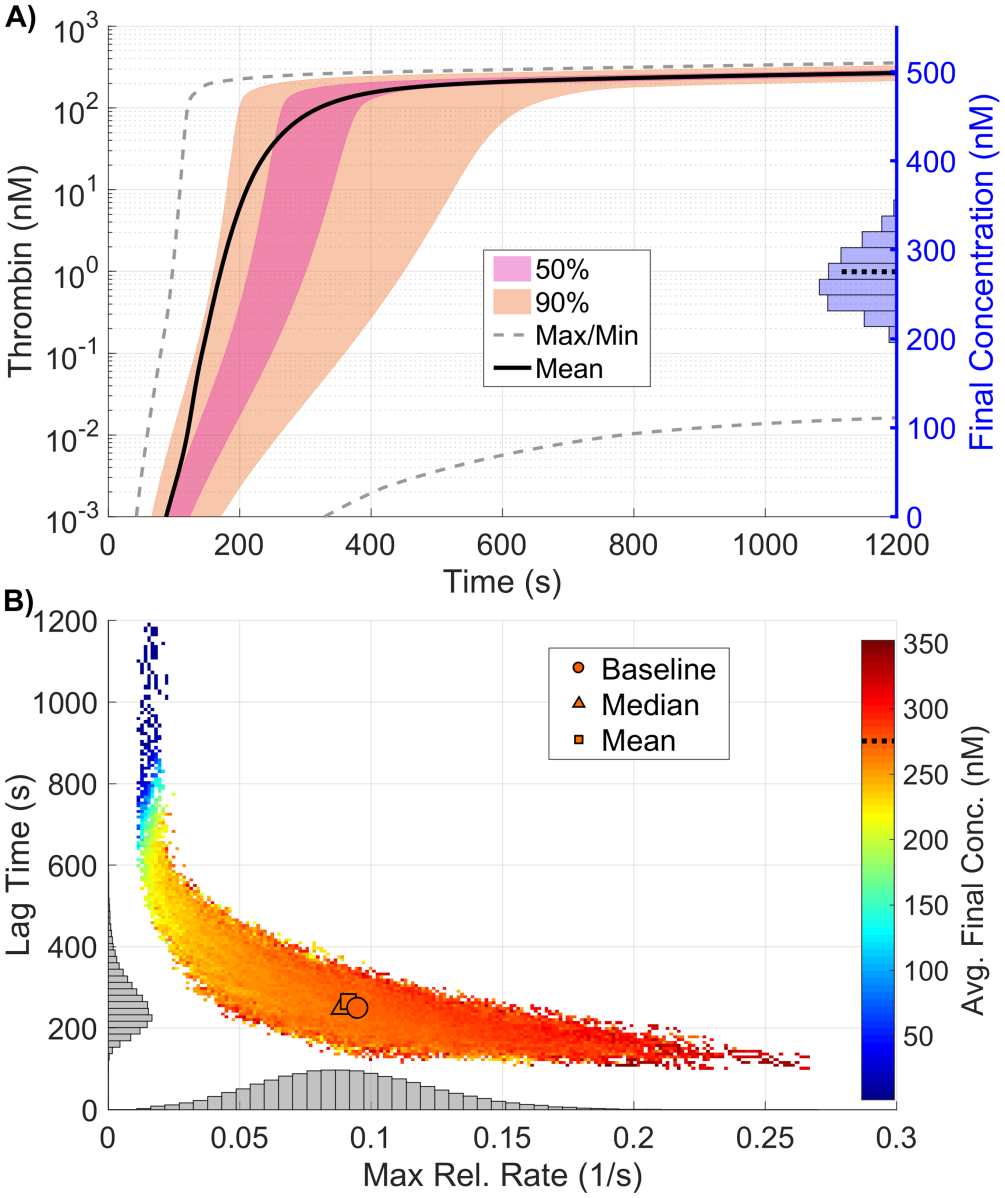
Variation in thrombin generation as a result of varying kinetic rate constants. **A)** *Left Axis:* Thrombin concentration time series showing the mean (solid black line) and boundaries that encompass 50% of the data (pink), 90% of the data (orange), and the maximum/minimum of the computed solutions (gray-dashed) generated by uniformly varying kinetic rates sampled uniformly between 50-150% of their nominal value simultaneously (270,000 total function evaluations). *Right Axis:* Marginal histogram of final thrombin concentration at *t* = 1200 seconds. **B)** Heatmap and marginal histograms relating three important thrombin generation metrics: lag time (*y-axis*), maximum relative rate (*x-axis*), and final concentration (*color-axis*). Results obtained by post-processing samples used to compute the global sensitivity indices. Dashed black bar in (A) and (B) represents the baseline case of 275nM of thrombin at 20 minutes.

Interestingly, the ability to simultaneously vary parameters results in radically different output from the model than seen when they are varied in a OAT fashion. This is most easily seen in the output distribution for thrombin (Fig 7A), where the support spans a significantly larger range than seen in the local SA of plasma levels. While the local SA found a maximum change of less than 30% across all metrics, the global SA produced variations exceeding 50% for *all three* thrombin generation metrics, even though parameters were varied over the same individual range. The observed output for the three metrics fell between 98-1200s for the lag time (39-482% of baseline), 0.011-0.267*s*^−1^ for the maximum relative rate (11-281% of baseline) and 0.02-355.69nM for the final concentration (0-129% of baseline).

### Varying platelet characteristics

**Fig 8:**
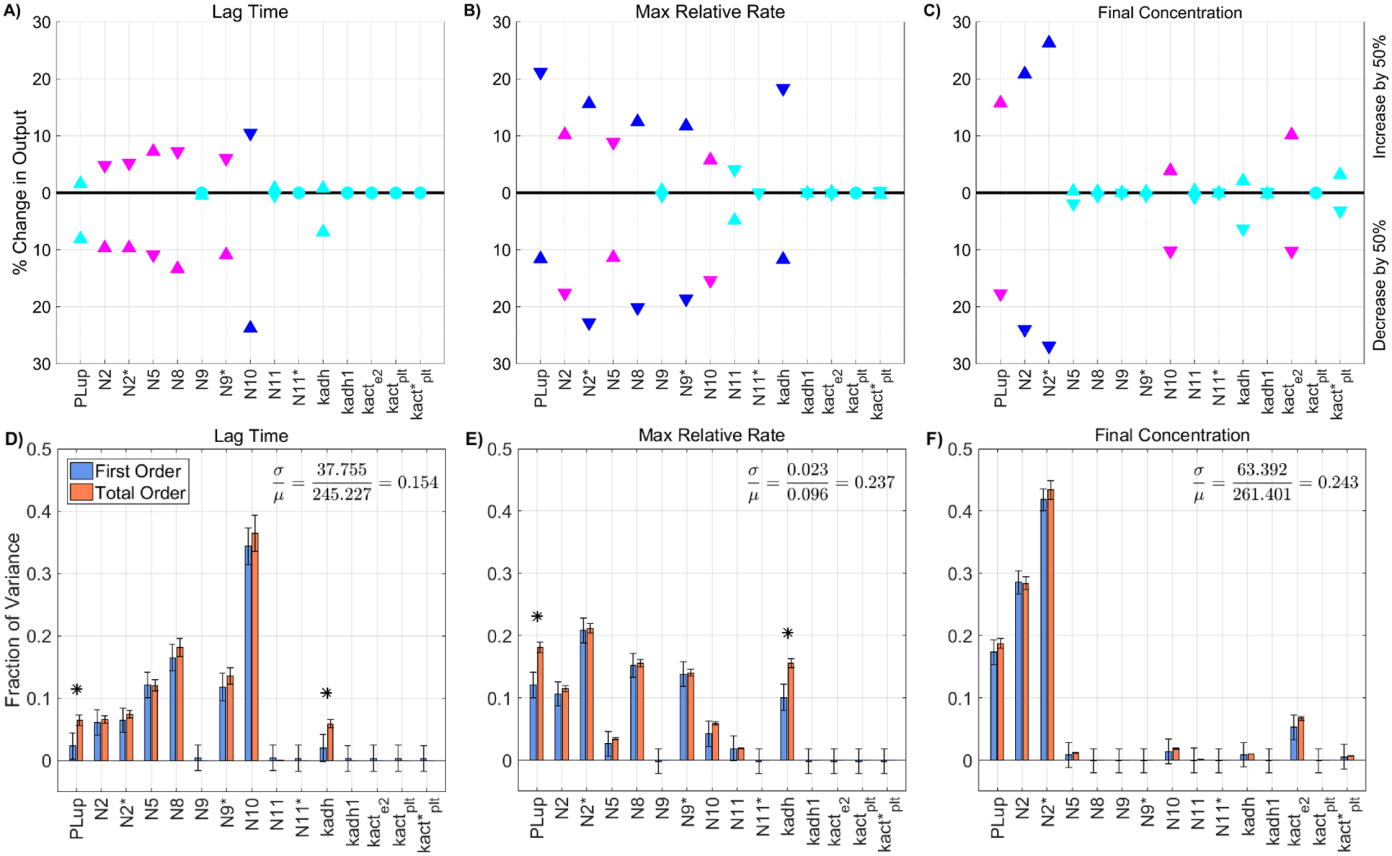
Sensitivity of thrombin generation to platelet characteristics. Variation in the **A,D)** lag time; **B,E)** maximum relative rate; **C,F)** final concentration; to platelet characteristics using the local (OAT) method (**A-C**) and global Sobol method (**D-F**). *Local*: Sensitivities $LS_{j}^{i}$ that lie between 0.75 and 1 (blue), between 0.25 and 0.75 (magenta), less than 0.25 (cyan) determine the rank-ordered list of platelet characteristics. The percent change of thrombin generation measures from standard model output for each initial condition are represented by triangles. The direction of variation is indicated with an upwards or downwards facing triangle. *Global*: First and Total Order Sobol indices are plotted as bars with errors of 2 standard deviation about the mean, computed with 5,000 bootstrap samples of the original 170,000 function evaluations. The coefficient of variation is included to provide a scale for the fraction of variance. PCs with Total Order index statistically significantly larger than the First order index are indicated with a star.

In this section we show how thrombin generation is modified due to variations in the platelet characteristics (PCs) in our model, specifically the rate of platelet adhesion to the subendothelium, rates of platelet activation by different agonists, platelet count (upstream platelet concentration), and the number and type of receptors/binding sites on activated platelet surfaces. We analyze local sensitivity by varying each platelet characteristic in a OAT fashion, from 50 to 150% of their baseline values. Fig 8A–8C show the most influential platelet characteristics for each thrombin metric; the triangle colors and directionality are as previously described. Fig 8D–8F show the first and total order Sobol indices computed for each platelet characteristic and metric, with the height of the bars indicating the fraction of variance that is attributable to the model output from each parameter individually (first order) and to the parameter including its interactions with other parameters (total order).

In Fig 8A–8C, we see that varying the number *N*_10_ of binding sites for fX/fXa on each activated platelet gives the greatest effect on the lag time; decreasing *N*_10_ by 50% leads to a 24% longer lag time. Variations in six platelet characteristics (binding site number per activated platelet for fIIa, fIXa, fVIII/fVIIIa and fII as well as platelet count and the platelet rate of adhesion) have the most effect on the maximum relative rate of thrombin generation (Fig 8B). A 50% reduction in platelet count or the rate of platelet adhesion increased the maximum relative rate by about 20%, while a 50% increase in these parameters decreased the maximum relative rate by about 10% (see Fig 8B). This reflects the role of platelet coverage of the injury in physically inhibiting TF:VIIa activity.

Lastly, the binding site numbers for fII and fIIa most dramatically affect the final thrombin concentration (Fig 8C). An increase of 50% in the value of $N_{2}^{*}$ or *N*_2_ leads to an increase of 28% or 22% in thrombin, respectively (Fig 8C). A 50% decrease in the two binding site numbers results in a 28% and 25% decrease in thrombin levels, respectively. Increasing or decreasing the binding site number for thrombin have the largest effects on the final concentration.

The global SA is in agreement with the findings from the local SA, with regard to the relative ranking of important parameters (*p* < 10^−6^) from a rank permutation test [54]). As seen when KRCs were varied, changes in the platelet characteristics had a much larger impact on the model variance in all three metrics than when plasma levels of zymogens and inhibition were varied, with coefficients of variations between 0.15 and 0.24. The lag time (Fig 8D) was most strongly influenced by variations in *N*_10_, with 37% of the variance explained by it alone. Two parameters, *PL*^*up*^, the upstream platelet concentration, and $k_{adh}^{+}$, the rate of platelet adhesion to the subendothelium, have statistically significant interaction effects (*p* < 0.05), as indicated by their increased total order indices (marked with a star). We note that the first order index for these two parameters are close to zero (less than 0.03), indicating that variations in the two parameters *individually* contribute little to the model output. Instead, only the interaction terms appear to be significant. This behavior is not directly observed in the local analysis, but it may be hinted at in the non-monotonic nature of the local SA endpoints for these two parameters (S2A Fig, solid black and dashed purple curves). For the maximum relative rate metric, several parameters have a non-zero Sobol index (Fig 8E), yet none explains more than 21% of the model output variance. Again, *PL*^*up*^ and $k_{adh}^{+}$ have statistically significant interaction effects but with non-trivial first order effects at 12% and 10%, respectively. For the final concentration of thrombin, almost 90% of the output variation can be explained by the first order effects of three parameters: *PL*^*up*^, *N*_2_, and $N_{2}^{*}$ with 17%, 29%, and 41% of the variance, respectively. It is interesting to note that while these parameters strongly influence the final thrombin concentration, they appear to do so in a nearly independent fashion, as no parameters appear to have significant interactions within this metric.

**Fig 9:**
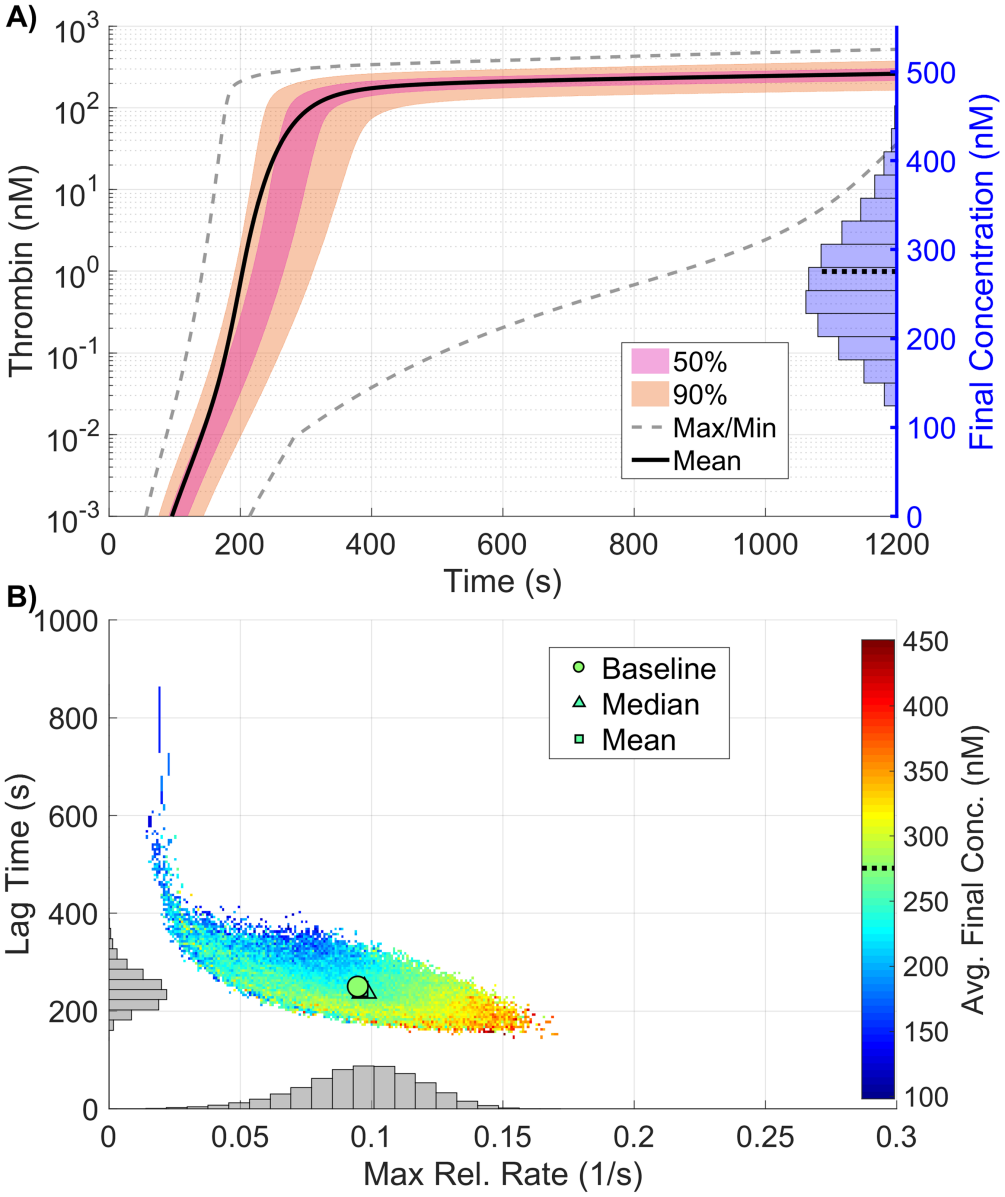
Variation in thrombin generation as a result of varying platelet characteristics. **A)** *Left Axis:* Thrombin concentration time series showing the mean (solid black line) and boundaries that encompass 50% of the data (pink), 90% of the data (orange), and the maximum/minimum of the computed solutions (gray-dashed) generated by varying platelet characteristics uniformly between 50-150% of their baseline values simultaneously (170,000 total function evaluations). *Right Axis:* Marginal histogram of final thrombin concentration at *t* = 1200 seconds. **B)** Heatmap and marginal histograms relating three important thrombin generation metrics: lag time (*y-axis*), maximum relative rate (*x-axis*), and final concentration (*color-axis*). Results obtained by post-processing samples used to compute the global sensitivity indices. Dashed black bar in (A) and (B) represents the baseline case of 275nM of thrombin at 20 minutes.

As with the KRC global results, the observed distribution of the thrombin curves (Fig 9A) and the resulting distribution of the coagulation metrics (Fig 9B), have a far larger variance than in the plasma level case. This is especially true for the final concentration of thrombin, where extremely high output is made possible by the combination of high platelet count and large numbers of fII/fIIa platelet binding sites (results not shown here).

### Varying all model parameters

In the previous sections, we examined both the local and global sensitivity resulting from variations in three classes of parameters: initial plasma levels, kinetic rate constants, and platelet characteristics. In this section, the three classes are combined and a sensitivity analysis on a subset of the parameters is performed. To identify a subset of parameters that have either a large individual effect on the thrombin output or interact strongly with other parameters, we again use the method of Morris to screen and select parameters. For this screening, the associated *ℓ*_2_-norm of the Morris sensitivity measures μ*, *σ* for lag time, maximum relative rate, and final concentration are shown in S6 Fig Parameters with the normalized *ℓ*_2_-norm of the Morris mean μ* and standard deviation *σ* greater than 0.5, for any of the paths, were chosen as candidates for the parameter subset.

The result of the screen is a subset of 33 parameters: four from plasma levels (*Z*_2_, *Z*_9_, *Z*_10_, *Z*_11_), six from platelet characteristics ($PL^{\text{up}},N_{2},N_{5},N_{9},N_{9}^{*}$, and $N_{11}^{*}$), with the remaining 23 from kinetic rate constants. There are six kinetic rate constants in the new subset that were *not* identified in the original Morris screen for KRCs only: $k_{z_{5}^{m}:e_{10}^{m}}^{cat}$, $k_{z_{8}^{m}:e_{2}^{m}}^{cat}$, $K_{PC:TM:e_{2}}^{\text{M}}$, $k_{z_{7}:e_{9}}^{-}$, and $k_{11}^{in}$. Six of the KRCs were also excluded from the new subset that *were* previously identified in the KRC-only Morris screen: $k_{10}^{\text{off}},k_{5}^{\text{off}},k_{2}^{\text{off},*},K_{z_{8}^{m}:e_{10}^{m}}^{\text{M}},K_{z_{8}^{m}:e_{2}^{m}}^{\text{M}}$, and $k_{e_{5}^{m}:e_{10}^{m}}^{+}$.

We note that some of the parameters show significant sensitivity, according to the Morris method, for *all three* of the thrombin generation metrics: the activation rates of fX by TF:VIIa and tenase ($k_{z_{10}^{m}:e_{7}^{m}}^{cat}$, $k_{z_{10}^{m}:TEN}^{cat}$), the activation rate of prothrombin by prothrombinase ($k_{z_{2}^{m}:PRO}^{cat}$), the binding rates of thrombin and fVIII/fVIII to a platelet surface ($k_{2}^{on,*}$, $k_{8}^{on}$), the unbinding rate of fIX/fIXa from a platelet surface ($k_{9}^{off}$), the inactivation rate of fXIa ($k_{11}^{in}$), and binding site numbers for fII, fIX/fIXa, and fXIa (*N*_2_, *N*_9_, $N_{11}^{*}$).

**Fig 10:**
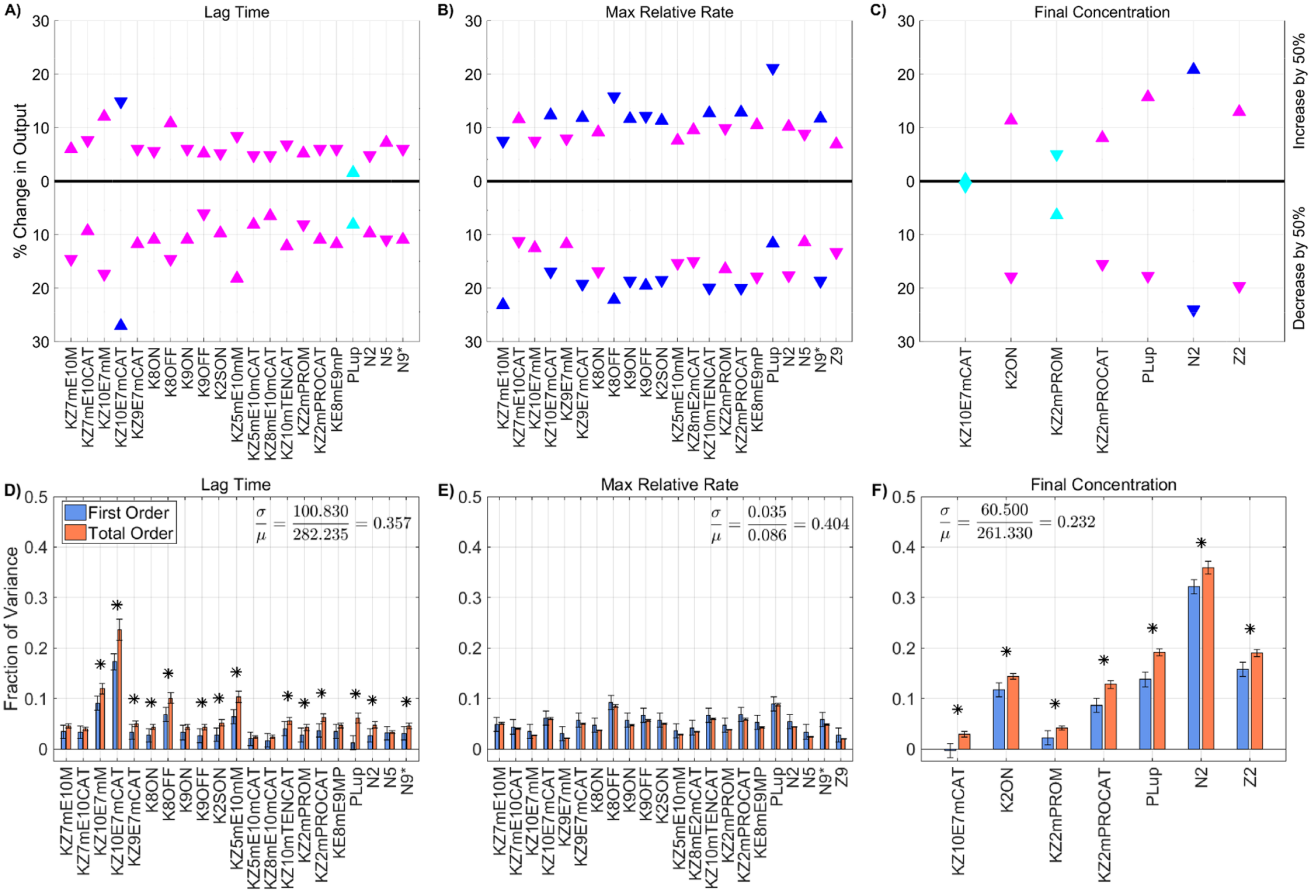
Sensitivity of thrombin generation to a subset of all model parameters. Variation in the **A,D)** lag time; **B,E)** maximum relative rate; **C,F)** final concentration; to a subset of all model parameters using the local (OAT) method (**A-C**) and global Sobol method (**D-F**). *Local*: Sensitivities $LS_{j}^{i}$ that lie between 0.75 and 1 (blue), between 0.25 and 0.75 (magenta), less than 0.25 (cyan) determine the rank-ordered list of platelet characteristics. The percent change of thrombin generation measures from standard model output for each initial condition are represented by triangles. The direction of variation is indicated with an upwards or downwards facing triangle. *Global*: First and Total Order Sobol indices are plotted as bars with errors of 2 standard deviation about the mean, computed with 5,000 bootstrap samples of the original 740,000 function evaluations. The coefficient of variation is included to provide a scale for the fraction of variance. PCs with Total Order index statistically significantly larger than the First order index are indicated with a star.

Next, a global SA was performed on the identified subset of parameters, in which the parameters again varied simultaneously between 50-150% of their baseline values. The results of the global SA were compared to those from the local SA, for each of the parameters in the subset, as shown in Fig 10. The local and global sensitivity analyses for the lag time and final concentration metrics agree in terms of ranking the parameter importance (*p* < 0.001 from a rank permutation test [54]), but for the maximum relative rate metric, the local and global methods were in less agreement (*p* < 0.1). The global Sobol information was able to identify interactions that influence the output variance for the lag time and the final concentration metrics (Fig 10D and 10F) but not for the maximum relative rate metric (Fig 10E).

In regard to the sensitivity of the lag time (see Fig 10D), the single parameter $k_{z_{10}:e_{7}^{m}}^{\text{cat}}$ had the largest individual effect, accounting for approximately 15% of the total model variance, with the remaining parameters individually contributing less than 10%. A sum of the non-zero first-order terms for the lag time reveals that approximately 87% of the variance in lag time can be described by the individual effects of the sampled parameters, with the remaining 13% being attributable to interactions between parameters. The large importance of first-order effects helps to explain why the local and global parameter rankings match so well, since first-order effects (the only effects that are measurable by local methods) dominate the system in this regime.

The variance in the final thrombin concentrations at 20 minutes (see Fig 10F) was dominated by the number of binding sites for prothrombin, *N*_2_, which accounted for more than 30% of the total model variance. Also playing a significant role was the plasma level of prothrombin, *z*_2_, the rate of prothrombin binding to platelet binding sites $k_{2}^{\text{on}}$, the rate of prothrombin activation by prothrombinase, $K_{z_{2}^{m}:PRO}^{\text{cat}}$, and the platelet count *PL*^*up*^. The sum of the first-order terms was approximately 84%, which means that about 16% of the output variance was due to parameter interactions.

While no significant interactions were detected for the maximum relative rate (Fig 10E), most of the parameters made small individual contributions to the output variance. A few of the parameters, namely $k_{2}^{\text{on}}$, $K_{PC:TM:e_{2}}^{\text{M}}$, $k_{11}^{\text{in}}$, *N*_9_, *N*_11_, and *z*_11_ had nearly zero effect as measured by first and total order Sobol indices. Of the parameters that did have an effect, we note that even though they each only contributed a small amount (less than 10% of the variance), there was still a high value for the coefficient of variation (about 40%). In other words, our global analysis revealed that there was high variation in the output, relative to the mean, due to a large number of individual, additive effects; this result would not have been captured with local SA alone.

**Fig 11:**
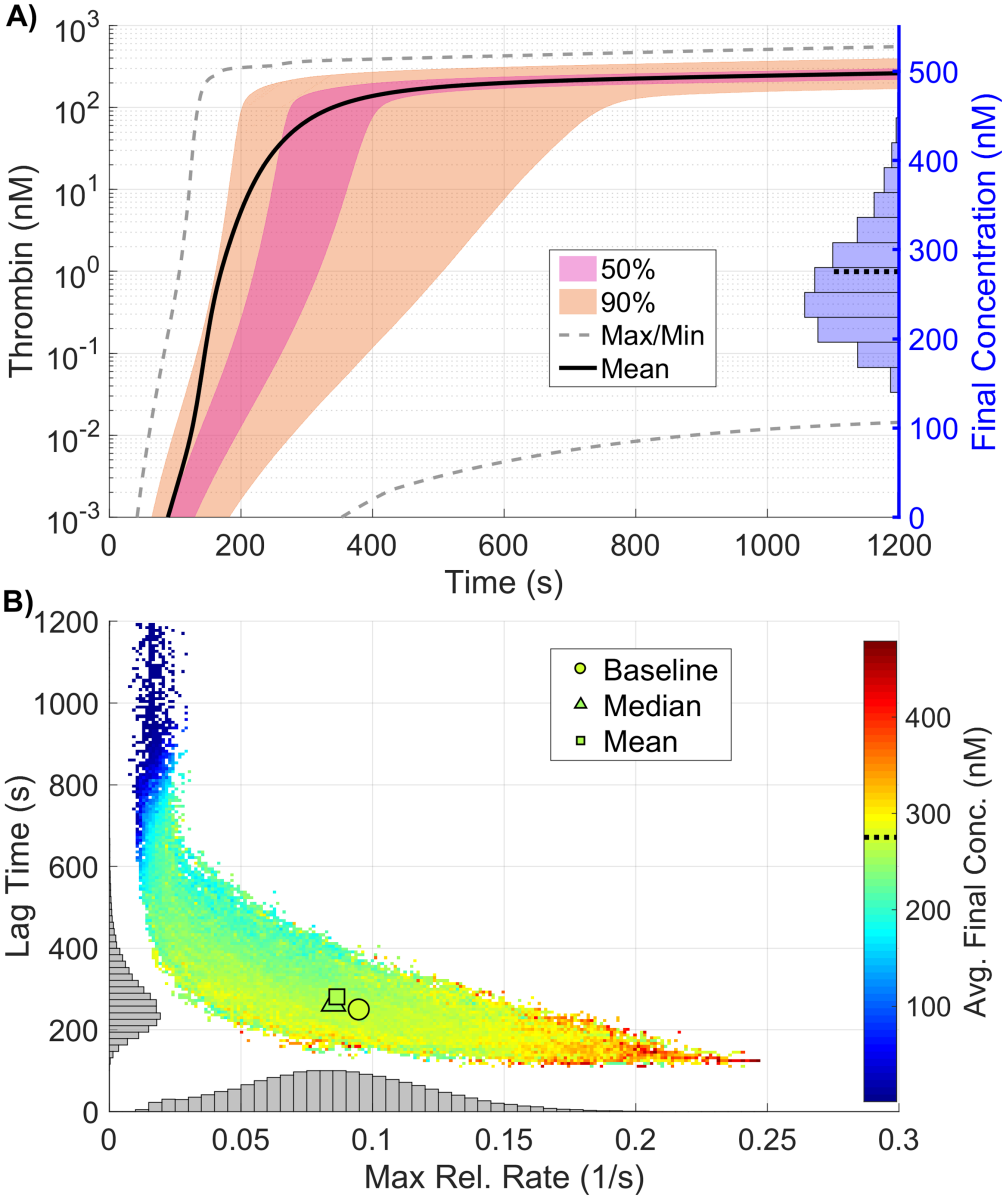
Variation in thrombin generation as a result of varying a subset of all model parameters. **A)** *Left Axis:* Thrombin concentration time series showing the mean (solid black line) and boundaries that encompass 50% of the data (purple), 90% of the data (orange), and the maximum/minimum of the computed solutions (gray-dashed) generated by uniformly varying a subset of all parameters (found via the Morris method) sampled between 50-150% of their nominal value simultaneously (740,000 total function evaluations). *Right Axis:* Marginal histogram of final thrombin concentration at *t* = 1200 seconds. **B)** Heatmap and marginal histograms relating three important thrombin generation metrics: lag time (*y-axis*), maximum relative rate (*x-axis*), and final concentration (*color-axis*). Results obtained by post-processing samples used to compute the global sensitivity indices. Dashed black bar in (A) and (B) represents the baseline case of 275nM of thrombin at 20 minutes.

The coefficient of variation was, in fact, high for *all three* thrombin metrics, relative to those from the previous studies in which we varied each parameter class individually. This means that simultaneously varying all three types of parameters leads to higher variation in the model output. This result can also be visualized by looking at the variation in thrombin time series output in Fig 11A. There we see very wide boundaries that encompass 90% of the data for the thrombin time series plots (orange region) and also a large total area within the grey dashed lines that represents the max/min boundary of all the data. In addition, the data revealed that almost 1% of the model evaluations in this case failed to generate 1nM of thrombin by 20 minutes (blue dots in heatmap in Fig 11B).

We found that for the lag time and the final concentration, a fraction of the variance originates from higher-order interactions among parameters whereas for the maximum relative rate, there were no interactions but a large number of individual additive effects. Because it is difficult to determine exactly which parameters are directly interacting and determine if they are interacting synergistically or antagonistically, we next further examine extreme behavior in the thrombin output to check for patterns or groupings in the parameter variations that lead to the extreme behavior.

#### Conditional input distributions for fast/slow thrombin production

To determine if there is a clear way to identify groupings of interacting parameters, we examined two extreme situations, one in which thrombin generation occurs quickly (fast bursts) and one in which thrombin forms very slowly (low producers). To do this we further filtered the simulations used for the global SA in Fig 11D–11F. In particular, we characterized those simulations that led to fast bursts or were low producers, defined by the smallest and largest 1% of the recorded lag times. In other words, we conditioned the input parameters on leading to fast or slow bursts, and then computed the resulting *conditional* distributions of parameters.

**Fig 12:**
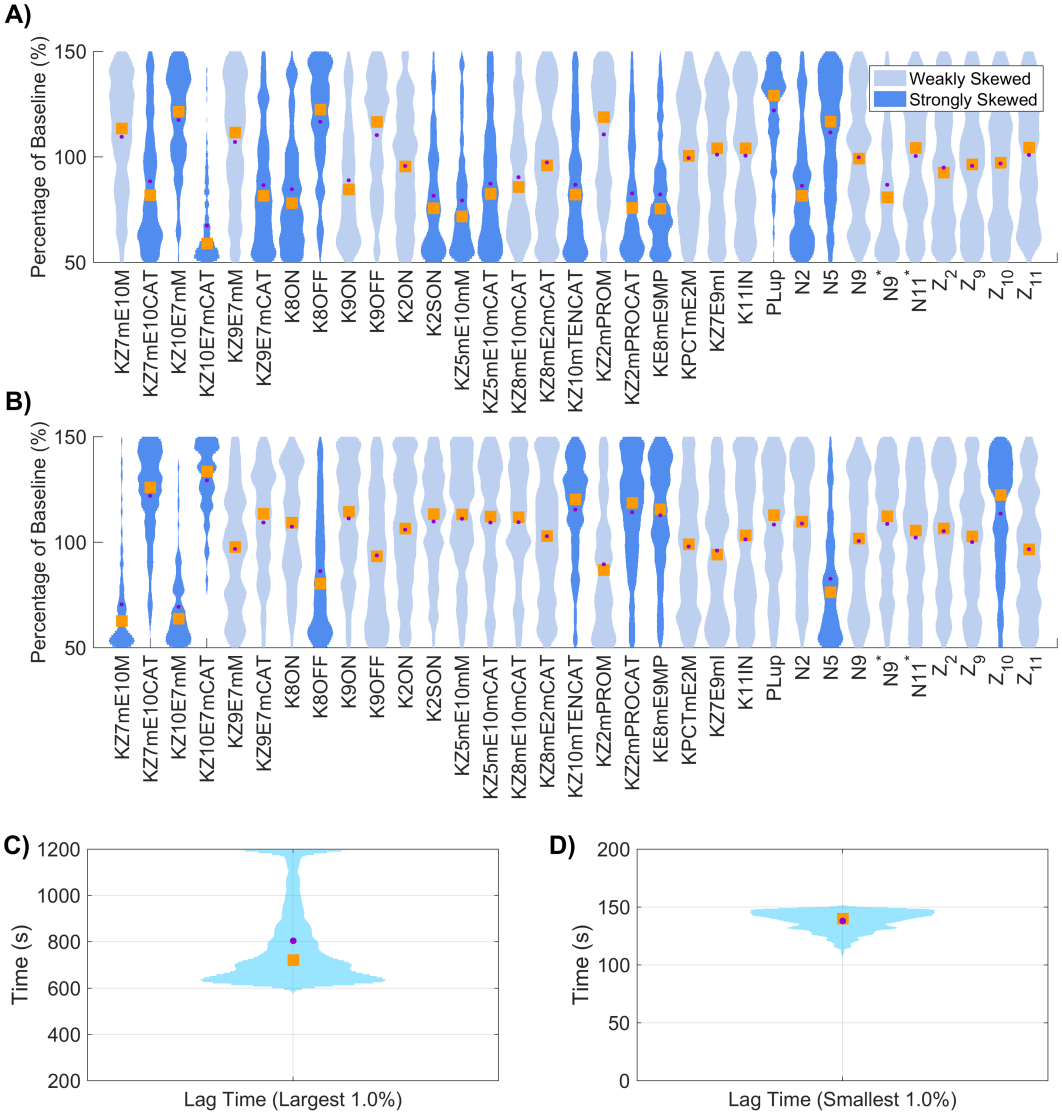
Conditional input distributions for globally varied subset of all parameters, of thrombin lag time and final concentration. The subset of parameters were sampled uniformly between 50-150% of their baseline value and the lag time of total thrombin was computed. The input distributions were conditioned on a slow burst: the largest 1% of lag time (A) and a fast burst: the smallest 1% of lag time (B). Distributions were colored dark blue if the empirical skew was greater than 0.5 in magnitude and light blue otherwise. Distributions of lag times for slow bursts (C) and fast bursts (D); skew shown when the mean (dot) and median (square) are not aligned.

Interesting patterns emerge in the resulting conditional parameter distributions, as shown in Fig 12. We see that specific combinations of parameters, when varied in concert, can achieve extreme responses in the thrombin production. For example, when we condition on low production of thrombin, (Fig 12A and 12C), we observe very low values for $k_{z_{10}:e_{7}^{m}}^{\text{cat}}$, $k_{2}^{*,\text{on}}$, $K_{z_{5}^{m}:e_{10}^{m}}^{\text{M}}$, $k_{z_{5}^{m}:e_{10}^{m}}^{\text{cat}}$, $k_{z_{10}^{m}:TEN}^{\text{cat}}$, $k_{z_{2}^{m}:PRO}^{\text{cat}}$, $k_{e_{8}^{m}:e_{9}^{m}}^{\text{+}}$, and *N*_2_ and a few others to a lesser degree. In addition, we see higher values of $K_{z_{10}:e_{7}^{m}}^{\text{M}}$, $k_{8}^{\text{off}}$, *PL*^up^, and *N*_5_. These parameter values slow the production of thrombin by *decreasing* the activation of fX by TF:VIIa, the rate that thrombin binds to platelet binding sites, the Michaelis-Menten constant for fV and fXa on the platelet surface and the catalytic rate for the same reaction, the catalytic rate for activation of fX by tenase, the catalytic rate for prothrombin conversion to thrombin by prothrombinase, the formation of tenase complexes, and the number of binding sites for prothrombin on the platelet surfaces. These parameter values slow the production of thrombin by *increasing* the Michaelis-Menten constant for fX and TF:VIIa on the platelet surface, the rate that fVIII/fVIIIa unbind from the platelet surface, the platelet count in the plasma, and the number of platelet binding sites for fV/fVa.

When we condition on fast thrombin production, (Fig 12B and 12D), we observe very low values for $K_{z_{7}:e_{10}^{m}}^{\text{M}}$, $K_{z_{10}:e_{7}^{m}}^{\text{M}}$, $k_{8}^{\text{off}}$, and *N*_5_, with higher values for $k_{z_{7}^{m}:e_{10}}^{\text{cat}}$, $k_{z_{10}:e_{7}^{m}}^{\text{cat}}$, $k_{z_{10}^{m}:TEN}^{\text{cat}}$, $k_{z_{2}^{m}:PRO}^{\text{cat}}$, $k_{e_{8}^{m}:e_{9}^{m}}^{\text{+}}$, and *Z*_10_. The skew of the distributions for the fast bursters are generally opposite from the skew of the same parameters for the low producers. In particular, reactions that increase/decrease the activation of fX by TF:VIIa intuitively affect the fast/slow production, including higher fX levels in the plasma. Similarly, the reactions that increase/decrease the activation of fX and prothrombin by tenase and prothrombinase intuitively affect the fast/slow production. Not so intuitively, perhaps, are the distributions for the binding rates for fVIII/fVIIIa to the platelet, the platelet count in the plasma, and binding site numbers for prothrombin and fV/fVa. But using our intuition with this model, we speculate that allowing fVIII/fVIIIa to stay bound to the platelets longer with fewer binding sites for fV/fVa can induce faster thrombin production in the following way: the more fVIIIa is available on the platelet surface, the more tenase could potentially form and thus the more fXa could be produced on the platelet surface; from here the fXa produced by the tenase feeds back and activates more bound fVIII, especially since there is less fV bound to the platelet that would compete with the fVIII for fXa. This is similar to the results in our previous study showing that fXa produced by tenase has an impact on the timing of the thrombin burst by feeding back to enhance more tenase formation [20]. Interestingly, the slow bursters are characterized by high numbers of binding sites for fV/fVa but also a highly skewed distribution for platelet count; here the platelets are inhibiting the system by covering the activity at the SE, similar to the interplay between fXI and platelet count reported in our previous study [16].

### Thrombin metric dependence on parameter class

Further inspection of Figs 5B, 7B, 9B and 11B leads to two important observations. First, we see that the mean of each thrombin metric is very close to the value produced when the input parameters are at their baseline level. Second, we see that these means are similar *independent* of parameter class variation. However, we have also demonstrated a wide range of coefficients of variation across the previous sections, with the highest values resulting from simultaneous variation of all three classes of parameters. Even though the thrombin metric means are similar, the spread in the thrombin metric output is shown to vary greatly across parameter class.

**Fig 13:**
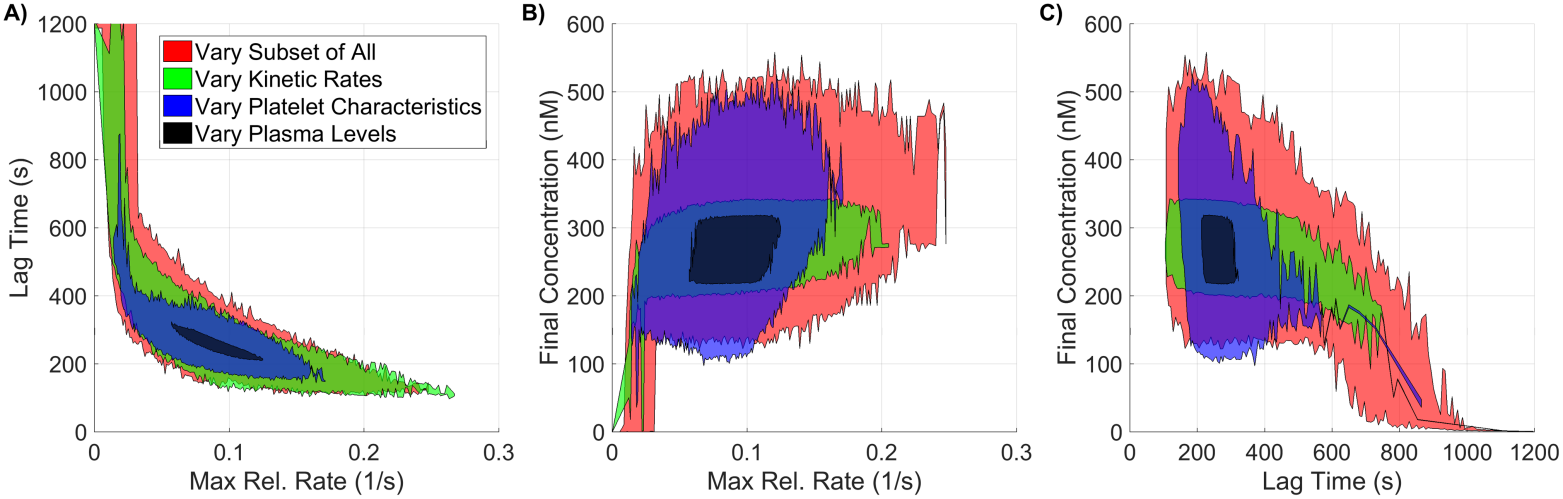
2D projections of ouput coagulation metric envelopes for different classes of varying inputs. Three classes of model parameters (plasma levels, a subset of important kinetic rate constants, and platelet characteristics) are varied within normal ranges. The envelope of three important coagulation metrics (lag time, maximum relative rate, and final concentration) are recorded and projected into two dimensions, where **A**, **B**, and **C** are the different orthogonal directions.

The thrombin metric outputs are the result of hundreds of thousands of samples through parameter space and can be represented as a three-dimensional cloud of points, where each dimension is a different thrombin metric: lag time, max relative rate, and final concentration. Note that we have compiled four of these point clouds, one for each of the parameter classes. To visualize the spread in the outputs, for each parameter class, we constructed a hull around the point clouds and projected them onto all possible two-dimensional planes to see how they compare. In Fig 13, we show these projections, with the result for each parameter class overlaid on top of one another, where plasma levels are in black, platelet characteristics are in blue, kinetic rate constants are in green, and the subset of all classes is red. It is clear from Fig 13 that the spread in the thrombin metric outputs is largest when all classes of parameters are varied simultaneously. In addition, we found that there are strong negative correlations among certain thrombin metrics. To quantify this, we computed the Pearson correlation coefficient between pairs of thrombin metrics. The correlation coefficients between the lag time and maximum relative rate showed strong negative correlation: -0.93, -0.68, -0.81, and -0.73, corresponding to variations in the plasma levels, kinetic rate constants, platelet characteristics, and subset of all classes. Interestingly, the Pearson correlation coefficients between lag time and final concentration were between -0.23 and -0.41, showing weak negative correlation, while the coefficients between maximum relative rate and final concentration ranged between 0.25 and 0.37, showing weak positive correlation.

### Varying flow

While higher blood flow velocities bring platelets and coagulation proteins to the site of injury at a faster rate, they also carry away enzymes produced at that site at a faster rate. Therefore, flow can both facilitate and inhibit thrombin generation and, thus, thrombus formation. This makes it difficult to intuit the exact response that the system will have to changes in flow. In this section, we show how thrombin generation in our model is modified due to variations in shear rate over the range of those found physiologically. To address overall variation in thrombin generation, samples using shear rates within the range of 1-1500 (1/s) were generated.

**Fig 14:**
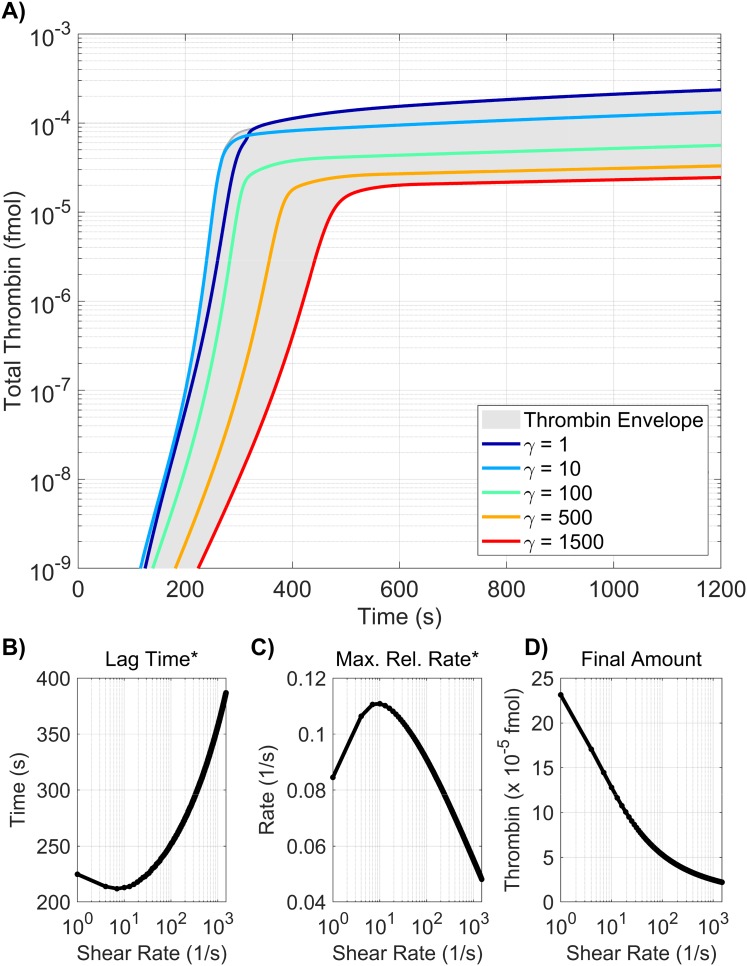
Variation in thrombin generation as a result of varying shear rate. **A)** Time series of the amount of thrombin showing the the maximum and minimum of the data (blue) generated by varying the shear rate from 1-1500 (1/s) as well as thrombin curves generated with shear rates 1, 10, 100, 500, and 1500 (1/s). Dependence of **B)** lag time*; **C)** maximum relative rate*; **D)** final amount on shear rate.

Fig 14A shows variations in thrombin generation with respect to time for several shear rates spanning the physiological range. Fig 14B–14D highlight the effect of shear rate on the three thrombin generation metrics. In our model, variations in shear rate lead to variations in the volume of the reaction zone in which the model’s reactions occur because of changes in the average boundary layer thickness. Therefore, rather than concentrations, we look at the *amount* (fmols) of thrombin in the reaction zone to assess the effect of shear rate on the system. To coordinate the three metrics of thrombin generation with those used in the previous analyses, we redefined the first metric to be the time to 2(10^−7^) fmol (lag time*). This quantity was determined using the average volume of the reaction zone for shear rate 100 (1/s) to calculate the number of moles of thrombin that are in the reaction zone when the thrombin concentration is 1 nM. The maximum relative rate* is calculated using the corresponding amounts of thrombin. The third metric is the amount of thrombin in the reaction zone at 20 minutes (final amount).

Lag time* varies from a little more than 200 seconds for shear rate 10/s to a little less than 400 seconds for shear rate 1500/s (Fig 14B). It is interesting that lag time* has a minimum for a shear rate about 10/s. The maximum relative rate* metric rises to a maximum at shear rate about 10/s and then falls by more than 50% as the shear rate increases to 1500/s (Fig 14C). The final amount of thrombin in the reaction zone decreases monotonically by about 10-fold as the shear rate increases from 1/s to 1500/s (Fig 14D). The decrease is fastest as the shear rate increases from 10/s to 150/s.

**Fig 15:**
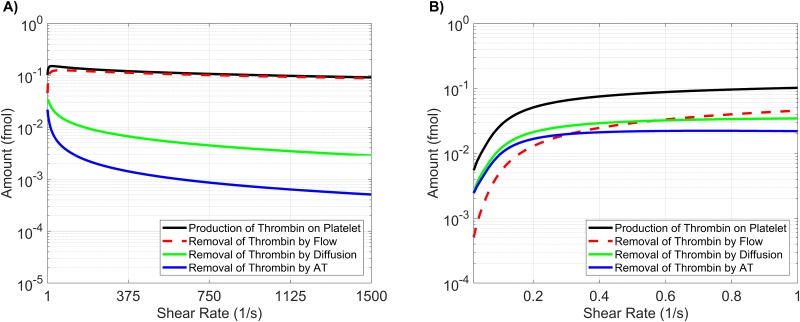
Effects of shear rate on production and removal of thrombin. Total production and removal of thrombin by flow, diffusion and chemical inhibition by antithrombin AT generated by varying shear rate from **A)** 1–1500 (1/s); **B)** 0.01–1 (1/s). At small and large shear rates (1-1500 (1/s)), the removal of thrombin is dominated by the effects of flow. Significantly smaller shear rates that approach static conditions result in diffusion and chemical inhibition dominated removal of thrombin. Static experiments support significant removal of thrombin by AT inhibition.

In Fig 15, we show the effect of shear rate on total thrombin production and on thrombin’s removal by flow, by lateral diffusion in a direction perpendicular to the flow direction, and by chemical inhibition by AT. For shear rates between 1/s and 1500/s, the removal of thrombin is dominated by the effects of flow. Indeed, the removal of thrombin by AT is approximately one hundred-fold smaller than that by flow. For very low shear rates (0.01/s to 0.4/s), the amount of thrombin removed by lateral diffusion or by chemical inhibition by AT is each larger than the amount removed by flow. This is consistent with static experiments which show significant removal of thrombin by AT inhibition [55, 56].

We also examined whether small variations in shear rate elicited large changes in model output that could effect the sensitivity analyses of this paper. To do this, we conducted the Method of Morris analysis on all model parameters at shear rates 90, 100, and 110 (1/s). Results of this analysis are shown in S3 Fig. From those plots it is clear that small changes in shear rate from the value 100/s used in most of our analyses do not effect the sensitivity of thrombin generation to variations in the model’s parameters, and do not effect the results of the Morris method based selection of parameters for the global SA.

## Discussion

In this study, we performed local and global sensitivity analyses of a mathematical model of coagulation and platelet deposition under flow. For the local analysis, input parameters were varied one at a time (OAT), whereas for the global methods, input parameters were varied simultaneously. We quantified the sensitivity of three thrombin metrics: lag time, maximum relative rate of generation, and final concentration after 20 minutes, to variations in three parameter classes: plasma levels, platelet characteristics, and kinetic rate constants. In addition, we examined sensitivity of the metrics to a subset of the union of all three parameter classes. For the cases of kinetic rate constants and all three parameter classes varied simultaneously, due to the large number of parameters under consideration, we performed subset selection using the method of Morris as a screening tool. Because our mathematical model considers flow and thus, the effect of flow on platelet adhesion and coagulation, we also examined the sensitivity of the thrombin metrics to changes in shear rate and the removal by flow, diffusion, and chemical inhibition/inactivation.

### Plasma levels

The local and global sensitivity analysis of the thrombin metrics due to variations in initial plasma levels were in good agreement with one another. No OAT variation in plasma levels led to more than a 20% change in any thrombin metric output; variations in prothrombin (*Z*_2_) produced the largest change in output, specifically in the final concentration. The results of global analysis reveals that there are no significant interactions between the plasma levels and thus suggests that the model is additive in this regime. The coefficient of variation for the maximum relative rate was 11% but was less than 1% for lag time and final concentration, indicating relatively small overall sensitivity of the model to normal variations in plasma levels. We also observed that all variations in plasma levels, using local or global methods, led to strong and steady thrombin generation.

To our knowledge, Danforth and colleagues [38] were the first and only other group to perform a sensitivity analysis on *thrombin metrics*, not simply overall thrombin sensitivity, and thus our results would compare more naturally with theirs than with other SA approaches on coagulation models. One limitation of their methodology, however, is the small number of parameters that they vary simultaneously (pairwise only), whereas our global methods vary all plasma levels simultaneously. Another difference is that their model simulated a closed system, which can provide a partial explanation for their observation that variations in initial levels of AT and TFPI had the most significant effects on the system. We also only compare to their data in which the plasma levels were varied within a normal range, but in their study and ours, there is always substantial thrombin generation by 20 minutes.

### Platelet characteristics

The local and global sensitivity analysis of the thrombin metrics due to variations in platelet characteristics were also in good agreement with each other. No OAT variation in platelet characteristics led to more than a 28% change in any thrombin metric output, and there was strong thrombin production in all cases. The prothrombin- and thrombin-specific binding site numbers, $N_{2},N_{2}^{*}$, produced the largest% change in the final concentration while the platelet count and adhesion rate, *PL*^*up*^, $k_{adh}^{+}$, produced the largest% change in the maximum relative rate. The global analysis highlights that *PL*^*up*^, $k_{adh}^{+}$ have statistically significant interaction effects. Interestingly, the non-monotonic behavior of the lag time when *PL*^*up*^ and $k_{\text{adh}}^{+}$ vary from 50% to 150% of the baseline values (S2 Fig) suggests such interaction effects. Both parameters are involved with platelet adhesion and determine how quickly the injury zone is paved over by platelets. Given that many reactions occur on platelet surfaces, it is intuitive that the thrombin metrics would be affected by the platelet count and rate of subendothelial coverage. The global analysis further suggests that the model is additive for most parameters in this regime. The coefficients of variation were 15%, 24%, and 24% for the lag time, maximum relative rate, and final concentration, respectively.

### Kinetic rate constants

The local sensitivity of thrombin metrics to kinetic rate constants (KRCs) showed that the maximum percent change in thrombin metric output due to OAT variation of any KRC is less than 30%. This means that in all of these simulations, there was always strong and steady thrombin generation. The global SA, however, showed something considerably different; when varying the KRCs simultaneously, there were situations (<1% of the total samples) where thrombin never reached 1 nM within 20 minutes. Interestingly, this resulted from variation in the KRCs from 50-150% of their baseline value. This is different from the results shown in the 2009 study by Danforth and colleagues [37]. In this early study by this group, overall thrombin sensitivity due to OAT variations of KRCs ranging from 10-1000% of baseline values was quantified, but not sensitivity of specific thrombin metrics. However, they showed that variations in the rate of TFPI inhibition (shown later to be one of their more sensitive parameters) still resulted in substantial thrombin generation by 20 minutes (see Fig 2A in [37]).

The global SA further revealed parameter interactions on a subset of KRCs selected by the method of Morris screen. Specifically, relating to the variance in lag time, the catalytic rate of activation of fX by TF:VIIa was found to interact with the rate that fVIII/fVIIIa unbind from the platelet surface, albeit these interactions only accounted for a small fraction of the variance. The coefficient of variation for the lag time was about 30%. The maximum relative rate metric had a larger coefficient of variation (37%) but there were no significant parameter interactions observed; all of the parameters accounted for small, additive, fractions of the variance. As for the variance in final concentration, the rate that prothrombin binds to the platelet surface accounted for about 50% of the variance but was not found to interact with other parameters, while the catalytic rate of activation of prothrombin by prothrombinase (accounting for about 30% of the variance) was shown to interact with other parameters that each accounted for only smal fractions of the variance. It was not possible to discern which of these parameters were specifically interacting with the others. The coefficient of variation for the final concentration was low, about 12%.

### Varying all parameter classes

To our knowledge, this is the first sensitivity analysis of a model of coagulation in which multiple parameter classes were varied simultaneously. A variation in the subset of all parameter classes led to the largest change in output of thrombin metrics, compared to variations in each parameter class alone. Interestingly, carrying out this variation in a OAT fashion leads to a maximum change in output of only about 25%, and again shows that every simulation in this case would lead to strong and steady thrombin generation. The global analysis further revealed potential interactions amongst parameters in regard to the lag time and final concentration while the maximum relative rate had the highest coefficient of variation but due to parameters that acted additively.

Again, the catalytic rate of activation of fX by TF:VIIa significantly affected the lag time, along with the rate that fVIII/fVIIIa unbinds from the platelet surface and the catalytic rate of activation of fV by fXa on the platelet surface. In addition to these KRCs, potential interactions were found between these and other parameters within the platelet characteristics class. The coefficient of variation for the lag time was about 36%. The final concentration metric was affected by multiple parameters from all classes, but involved platelet count and parameters associated with prothrombin: the plasma level for prothrombin, the binding rate for prothrombin to the surface of platelets, the number of platelet binding sites for prothrombin, and the catalytic rate of activation of prothrombin by prothrombinase on the platelet surface. The coefficient of variation for the final concentration was 23%. To better understand how the interactions affected extreme thrombin behavior, we conditioned the input distributions for globally varied parameters for fast/slow thrombin bursts. With this conditioning, we were able to clearly identify groups of parameters that resulted in fast/slow bursting behavior.

### Flow

We found that as the shear rate is increased from 1 s^−1^ to 1500 s^−1^, there is non-monotonic behavior in the lag time and the maximum relative rate, while the final thrombin amount decreases monotonically in this regime. A potential explanation for these results, as shown in Figs 14A–14D and 15, is the following. At large shear rates the height of the average chemical boundary layer and the platelet layers is smaller, producing a thinner reaction zone. Although higher shear rates imply that more platelets are brought into the reaction zone by the flow, the flow simultaneously washes more platelets away, which results in fewer platelets in the reaction zone overall (and thus, fewer platelets surfaces on which reactions may occur), although this also depends on adhesion and activation rates, proving that the effect of flow on each individual process is complex. We found, however, that there was both fewer active platelet surfaces and an increase in the removal of thrombin by flow that together, resulted in longer lag times, slower maximum relative rates of generation, and smaller final amounts.

### Limitations of our methodology

While our results from global SA showed considerable interactions between kinetic parameters, we emphasize that these results depend on the specific distributions and ranges that were chosen for each parameter. Because the uniform distribution is a maximum entropy distribution, it is possible that we have either over or underrepresented interaction strengths. Beyond the shape of the distribution, the range of viable kinetic rate constant values would similarly impact these findings. More accurate information on reasonable ranges of these values could greatly improve the ability of global SA methods to determine meaningful parameter interactions. In addition, we have assumed that parameters are independently distributed. However, if these values are in fact correlated among individuals (perhaps through a common genetic or biochemical mutation) an independent sampling method may again over or underrepresent interaction strengths depending on the functional consequences of the correlation. Finally, although the Morris method is a common screening tool for large sets of parameters, we note it is possible that in our global SA method we have missed interactions due to this screening method.

We note that the effects of flow and diffusion are given simplified treatment in this model. Despite these assumptions, results from this model have led to multiple experimentally-validated hypotheses regarding the TF-dependent threshold behavior of thrombin [57] and the synergistic behavior between TF and exogenous FXIa [20]. Extensions to this model include detailed fluid dynamics together with a continuum model of platelet aggregation [17, 18]. Performing sensitivity analysis of those model extensions will be the focus of future work.

### Local versus global analysis: What have we learned?

One important question that has resulted from this study is how to determine if and when it is more appropriate to use a local versus a global SA on models of complex biological systems. In terms of identifying and ranking our model’s most sensitive parameters, we found that the results of the local and global SA are in excellent agreement with one another, *in all cases*. However, we found that there were cases where if we wanted to explain the details of the variance in model outputs, the impact on the variance from parameters was not independent. This suggests that if a parameter ranking is the primary interest, a local SA may be sufficient, but if precise quantitative attribution of model output variance is necessary, global SA should be employed.

Beyond the information on parameter interactions, our global SA determined parameters whose variation significantly contributes to the variance of model output by themselves. In addition, the global SA provided the opportunity to examine subsets of input parameter space which were linked with strongly extremal output behavior, such as having a lag time in excess of 20 minutes despite still being in normal parameter ranges. Global SA results determined groupings of parameters that are more/less likely to lead to fast/slow thrombin bursting behavior. Finally, we note that all of our analyses were based on analyzing normal parameter ranges, it is possible that dependencies between parameters will be even stronger, and thus the global SA is more appropriate when considering disease states. Results of the local SA, method of Morris, and the global SA motivated the construction of a tailored sensitivity analysis approach for the model system described above. However, this methodology could potentially be employed to help identify biochemical and biophysical parameters that determine bleeding phenotype in some bleeding disorders, which is the focus of our future work.

## Supporting information

S1 Fig Monotonicity of change in thrombin generation due to variation in KRCs. Variation in the three physiologically relevant metrics of thrombin generation resulting from changes in kinetic rate constants.
(PDF)

S2 Fig Monotonicity of change in thrombin generation due to variation in PCs. Variation in the three physiologically relevant metrics of thrombin generation resulting from changes in platelet characteristics.
(PDF)

S3 Fig Sensitivity of MM screening results to shear rate. Results for the method of Morris procedure with trajectory selection at mulitple shear rates, demonstrating the insensitivity of selected parameters to shear rate.
(PDF)

S4 Fig Sensitivity of thrombin generation to all KRCs using OAT method. Variation in the lag time, maximum relative rate, and final concentration to the top 25 kinetic rate constants using the local (OAT) method.
(PDF)

S5 Fig Screening for KRCs using the method of Morris. Results for the method of Morris procedure with trajectory selection on the set of kinetic rate constants used to generate a collection of candidate parameters for global analysis.
(PDF)

S6 Fig Screening for all model parameters using the method of Morris. Results for the method of Morris procedure with trajectory selection on the set of all model parameters used to generate a collection of candidate parameters for global analysis.
(PDF)

S1 Appendix Notation, equations, and parameters. (PDF)
