# Supplementary material for: A local and global sensitivity analysis of a mathematical model of coagulation and platelet deposition under flow
Source: PLoS One. 2018 Jul 26;13(7):e0200917. doi: 10.1371/journal.pone.0200917 (PMC6062055; doi:10.1371/journal.pone.0200917)

S1 Fig. Monotonicity of Change in Thrombin Generation due to Variation in KRCs. Variation in the three physiologically relevant metrics of thrombin generation: A) lag time; B) maximum relative rate of thrombin generation; 3) final concentration of thrombin: due to variations in the top eight model sensitive kinetic rate constants.

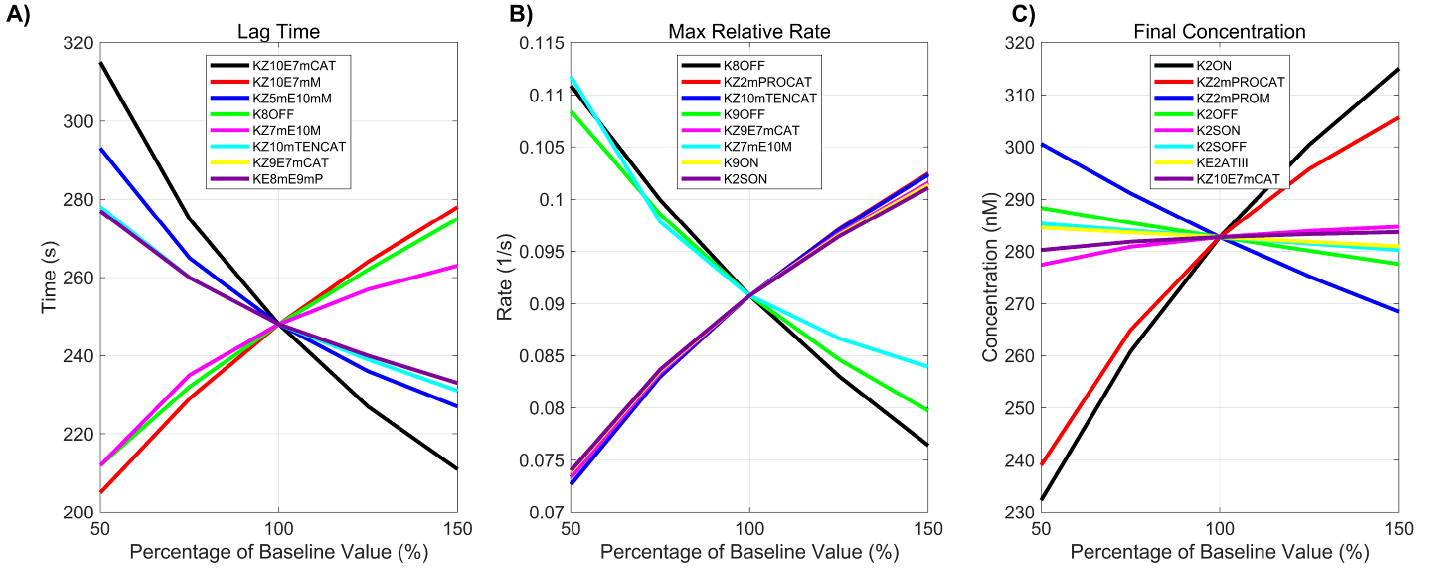

Supplement: S1 Fig — Variation in the three physiologically relevant metrics of thrombin generation resulting from changes in kinetic rate constants. (PDF) [file pone.0200917.s001.pdf]
