# Supplementary material for: A local and global sensitivity analysis of a mathematical model of coagulation and platelet deposition under flow
Source: PLoS One. 2018 Jul 26;13(7):e0200917. doi: 10.1371/journal.pone.0200917 (PMC6062055; doi:10.1371/journal.pone.0200917)

S3 Fig. Sensitivity of MM screening results to shear rate. The method of Morris procedure with trajectory selection was conducted and the associated ℓ_2-norm of the Morris sensitivity measures μ^*, σ for **A)** lag time*; **B)** maximum relative rate*; **C)** final amount; were calculated for shear rate 90, 100, and 110 (1/s). Parameters with the normalized ℓ_2-norm of the Morris mean μ^* and standard deviation σ greater than 0.5 for any of the path subsets were chosen to highlight that the results of the method of Morris are not sensitive to perturbations in shear rate.

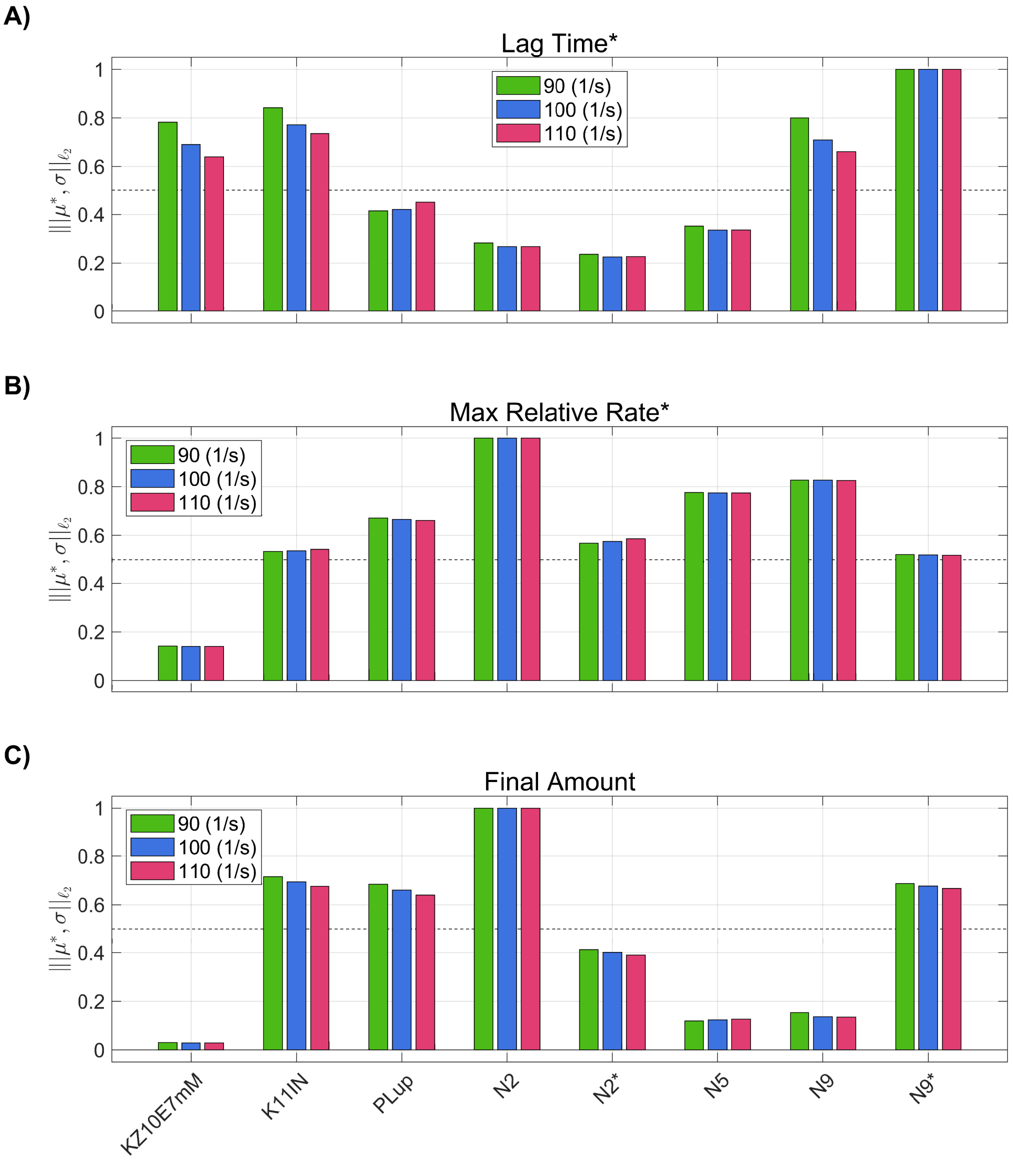

Supplement: S3 Fig — Results for the method of Morris procedure with trajectory selection at mulitple shear rates, demonstrating the insensitivity of selected parameters to shear rate. (PDF) [file pone.0200917.s003.pdf]
