# Supplementary material for: A local and global sensitivity analysis of a mathematical model of coagulation and platelet deposition under flow
Source: PLoS One. 2018 Jul 26;13(7):e0200917. doi: 10.1371/journal.pone.0200917 (PMC6062055; doi:10.1371/journal.pone.0200917)

S4 Fig. Sensitivity of thrombin generation to all KRCs using OAT method. Variation in the A) lag time; B) maximum relative rate; C) final concentration; to the top 25 kinetic rate constants using the local (OAT) method. Sensitivities LS_j^i that lie between 0.75 and 1 (blue), between 0.25 and 0.75 (magenta), less than 0.25 (cyan) determine the rank-ordered list of initial levels. The percent change of thrombin generation measures from standard model output for each initial condition are represented by triangles. The direction of variation is indicated with an upwards or downwards facing triangle.

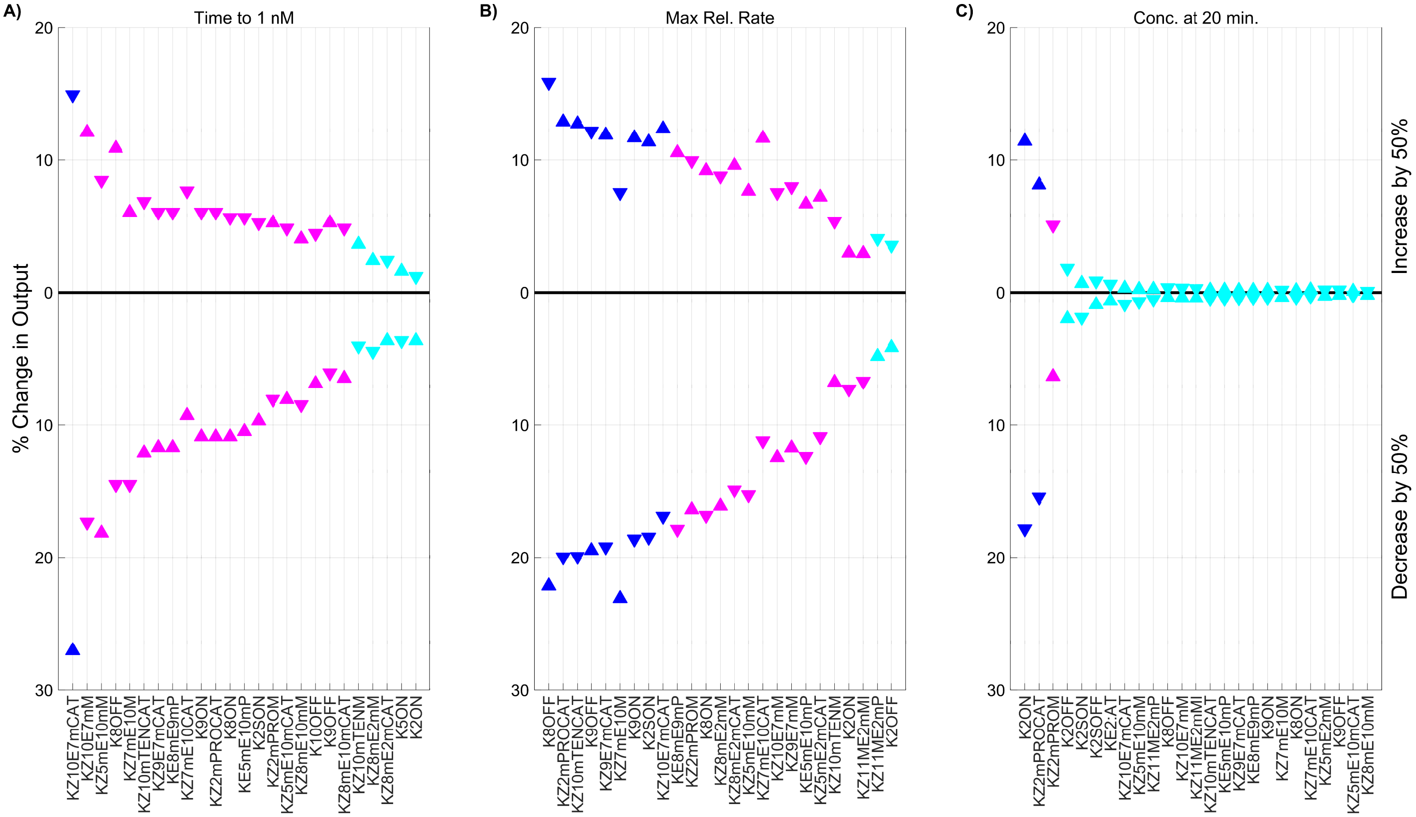

Supplement: S4 Fig — Variation in the lag time, maximum relative rate, and final concentration to the top 25 kinetic rate constants using the local (OAT) method. (PDF) [file pone.0200917.s004.pdf]
