# Supplementary material for: A local and global sensitivity analysis of a mathematical model of coagulation and platelet deposition under flow
Source: PLoS One. 2018 Jul 26;13(7):e0200917. doi: 10.1371/journal.pone.0200917 (PMC6062055; doi:10.1371/journal.pone.0200917)

S6 Fig. Screening for all model parameters using the Method of Morris. The method of Morris procedure with trajectory selection was conducted three times to generate path sets P_1, P_2, P_3. The associated ℓ_2-norm of the Morris sensitivity measures μ^*, σ for **A)** lag time; **B)** maximum relative rate; **C)** final concentration; were calculated. Parameters with the normalized ℓ_2-norm of μ^* and σ greater than 0.5 for any of the path subsets were chosen as candidates for the global Sobol method, determining a 33 model parameter subset.

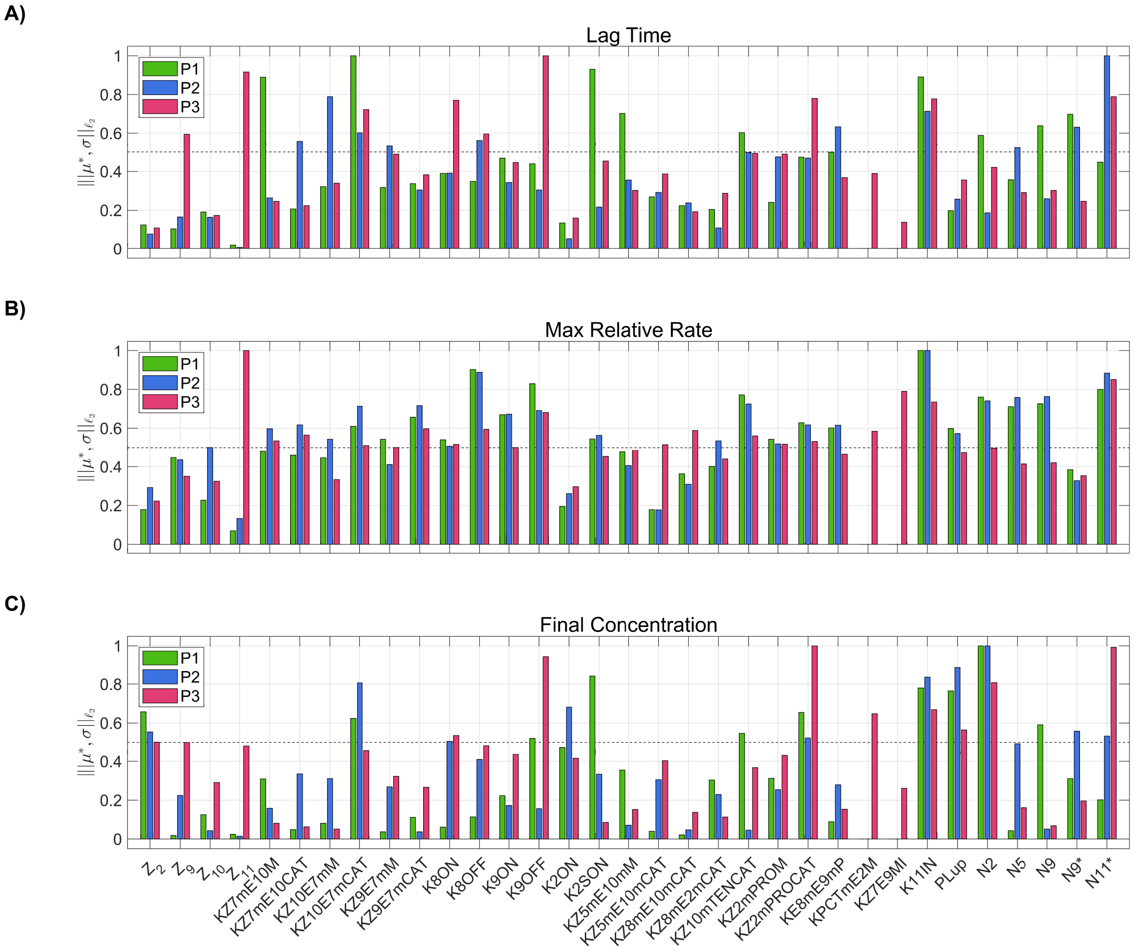

Supplement: S6 Fig — Results for the method of Morris procedure with trajectory selection on the set of all model parameters used to generate a collection of candidate parameters for global analysis. (PDF) [file pone.0200917.s006.pdf]
