# Supplementary material for: A local and global sensitivity analysis of a mathematical model of coagulation and platelet deposition under flow
Source: PLoS One. 2018 Jul 26;13(7):e0200917. doi: 10.1371/journal.pone.0200917 (PMC6062055; doi:10.1371/journal.pone.0200917)
Supplement: S1 Appendix — (PDF) [file pone.0200917.s007.pdf]

# S1 Appendix. Notation, equations, and parameters

Kathryn G. Link[2◑], Michael T. Stobb[3◑], Jorge A. Di Paola[5†], Keith B. Neeves [5,6†], Aaron L. Fogelson[1,2‡], Suzanne S. Sindi[3‡], Karin Leiderman[4‡*],

**1** Department of Mathematics, University of Utah, Salt Lake City, UT, USA
**2** Department of Bioengineering, University of Utah, Salt Lake City, UT, USA
**3** Department of Applied Mathematics, University of California, Merced, Merced, CA, USA
**4** Department of Applied Mathematics and Statistics, Colorado School of Mines, Golden, CO, USA
**5** Department of Pediatrics, University of Colorado School of Medicine, Aurora, CO, USA
**6** Department of Chemical and Biological Engineering, Colorado School of Mines, Golden, CO, USA

◑ These authors contributed equally to this work.
†These authors contributed equally to this work.
‡These authors contributed equally to this work.
*Corresponding author: kleiderman@mines.edu

S1-S4 Tables list input parameters that describe the physical processes and coagulation reactions we consider listed in S5-S12 Tables. $Z_i$ and $E_i$ refer to zymogen $i$ and enzyme $i$ in solution. A superscript 'm' indicates a membrane-bound versions of these proteins (e.g., $E_7^m$ refers to the TF:VIIa complex and $E_5^m$ refers to Factor Va bound to the platelet surface). Concentrations are denoted in a similar way but with lower-case $z$ and $e$. A complex of $Z_i$ and $E_j$ is denoted $Z_i : E_j$ and its concentration is denoted $[Z_i : E_j]$. Special symbols are used for the platelet-bound 'tenase' VIIIa:IXa and 'prothrombinase' Va:Xa complexes, $TEN$ = VIIIa:IXa and $PRO$= Va:Xa, and $[TEN]$ and $[PRO]$ denote their respective concentrations. The special symbol $TFPIa$ is used for the fluid-phase complex TFPI:Xa, and $[TFPIa]$ denotes its concentration. The inhibitors are denoted $APC$ and $TFPI$ and their concentrations are denoted $[APC]$ and $[TFPI]$.

The concentrations of unactivated, subendothelial bound, and activated but not subendothelial bound platelets are denoted $PL$, $PL_s^a$, and $PL_a^v$, respectively. Platelet binding sites for coagulation proteins are denoted $P_i$ or $P_i^*$. The former refers to binding sites for the zymogen $i$ or for zymogen and enzyme $i$. The latter refers to binding sites only for enzyme $i$. The number of $P_i$ or $P_i^*$ binding sites is denoted $N_i$ or $N_i^*$. The concentration $p_i$ or $p_i^*$ of each of these binding sites is needed in the model equations. It is obtained by multiplying the corresponding $N_i$ or $N_i^*$, respectively, by the concentration of activated platelets $PL_s^a + PL_v^a$.

Further discussion of model assumptions and parameter estimation can be found in [1, 2].

$$
\begin{aligned}
[TF]^{\text{avail}} &= [TF] - z_7^m - e_7^m - [Z_7^m : E_{10}] - [Z_7^m : E_2] - [Z_{10} : E_7^m] \\
&\quad -[Z_9 : E_7^m] - [TPFI : E_{10} : E_7^m] - [Z_7^m : E_9] \\
p_{PLAS}^{\text{avail}} &= p_{PLAS} - [PL_a^s] \\
p_5^{\text{avail}} &= p_5 - z_5^m - e_5^m - [Z_5^m : E_{10}^m] - [Z_5^m : E_2^m] \\
&\quad -[APC : E_5^m] - [PRO] - [Z_2^m : PRO] \\
p_8^{\text{avail}} &= p_8 - z_8^m - e_8^m - [TEN] - [Z_8^m : E_{10}^m] - [Z_8^m : E_2^m] \\
&\quad -[Z_{10}^m : TEN] - [APC : E_8^m] - [TEN^*] - [Z_{10}^m : TEN^*] \\
p_9^{\text{avail}} &= p_9 - z_9^m - e_9^m - [TEN] - [Z_{10}^m : TEN] \\
&\quad -[Z_9^m : E_{11}^{h,m}] - [Z_9^m : E_{11}^{m*}] \\
p_9^{*,\text{avail}} &= p_9^* - e_9^{m*} - [TEN^*] - [Z_{10}^m : TEN^*] \\
p_{10}^{\text{avail}} &= p_{10} - z_{10}^m - e_{10}^m - [Z_5^m : E_{10}^m] - [Z_8^m : E_{10}^m] \\
&\quad -[PRO] - [Z_2^m : PRO] - [Z_{10}^m : TEN] - [Z_{10}^m : TEN^*]
\end{aligned}
$$

$$
\begin{aligned}
p_2^{\text{avail}} &= p_2 - z_2^m - [Z_2^m : PRO] \\
p_2^{*,\text{avail}} &= p_2^* - e_2^m - [Z_5^m : E_2^m] - [Z_8^m : E_2^m] - [Z_{11}^m : E_2^m] - [E_{11}^{h,m*} : E_2^m] \\
p_{11}^{\text{avail}} &= p_{11} - z_{11}^m - e_{11}^{h,m} - [Z_9^m : E_{11}^{h,m}] - [Z_{11}^m : E_2^m] \\
p_{11}^{*,\text{avail}} &= p_{11}^* - e_{11}^{h,m*} - e_{11}^{m*} - [Z_9^m : E_{11}^{m*}] - [E_{11}^{h,m*} : E_2^m] \\
[TM]^{\text{avail}} &= ([TM] - [TM : E_2^{ec}] - [TM : E_2^{ec} : APC])
\end{aligned}
$$

$$\begin{aligned}
\frac{d}{dt}\ z_7 \quad &= \quad k_{\text{flow}}(z_7^{\text{up}} - z_7) - k_7^{\text{on}} z_7 [TF]^{\text{avail}} + k_7^{\text{off}} z_7^m - \ k^+_{z_7:e_2} z_7 e_2 \\
&\quad\ +k^-_{z_7:e_2}[Z_7 : E_2] - \ k^+_{z_7:e_{10}} z_7 e_{10} + k^-_{z_7:e_{10}}[Z_7 : E_{10}]
\end{aligned}$$

$$\begin{aligned}
\frac{d}{dt}\ e_7 \quad &= \quad k_{\text{flow}}(e_7^{\text{up}} - e_7) - k_7^{\text{on}} e_7 [TF]^{\text{avail}} + k_7^{\text{off}} e_7^m \\
&\quad\ +k^{\text{cat}}_{z_7:e_2}[Z_7 : E_2] + k^{\text{cat}}_{z_7:e_{10}}[Z_7 : E_{10}]
\end{aligned}$$

$$\begin{aligned}
\frac{d}{dt}\ z_7^m \quad &= \quad k_7^{\text{on}} z_7 [TF]^{\text{avail}} - k_7^{\text{off}} z_7^m - k^+_{z_7^m:e_{10}} z_7^m e_{10} + k^-_{z_7^m:e_{10}}[Z_7^m : E_{10}] \\
&\quad\ -k^+_{z_7^m:e_2} z_7^m e_2 + k^-_{z_7^m:e_2}[Z_7^m : E_2] - z_7^m \frac{d}{dt}\ [PL_a^s] \frac{1}{p^{\text{avail}}_{PLAS}}
\end{aligned}$$

$$\begin{aligned}
\frac{d}{dt}\ e_7^m \quad &= \quad k_7^{\text{on}} e_7 [TF]^{\text{avail}} - k_7^{\text{off}} e_7^m \\
&\quad\ -k^+_{TPFI:e_{10}:E_7^m} e_7^m [TFPI : E_{10}] + k^-_{TPFI:e_{10}:E_7^m}[TPFI : E_{10} : E_7^m] \\
&\quad\ +k^{\text{cat}}_{z_7^m:e_{10}}[Z_7^m : E_{10}] + k^{\text{cat}}_{z_7^m:e_2}[Z_7^m : E_2] \\
&\quad\ +(k^{\text{cat}}_{z_{10}:e_7^m} + k^-_{z_{10}:e_7^m})[Z_{10} : E_7^m] - k^+_{z_{10}:e_7^m} z_{10} e_7^m \\
&\quad\ +(k^{\text{cat}}_{z_9:e_7^m} + k^-_{z_9:e_7^m})[Z_9 : E_7^m] - k^+_{z_9:e_7^m} z_9 e_7^m - e_7^m \frac{d}{dt}\ [PL_a^s] \frac{1}{p^{\text{avail}}_{PLAS}}
\end{aligned}$$

$$\begin{aligned}
\frac{d}{dt}\ z_{10} \quad &= \quad k_{\text{flow}}(z_{10}^{\text{up}} - z_{10}) - k_{10}^{\text{on}} z_{10}\ p_{10}^{\text{avail}} + k_{10}^{\text{off}} z_{10}^m - k^+_{z_{10}:e_7^m} z_{10} e_7^m \\
&\quad\ +k^-_{z_{10}:e_7^m}[Z_{10} : E_7^m]
\end{aligned}$$

$$\begin{aligned}
\frac{d}{dt}\ e_{10} \quad &= \quad k_{\text{flow}}(e_{10}^{\text{up}} - e_{10}) - k_{\text{diff}}(e_{10} - e_{10}^{ec}) - k_{10}^{\text{on}} e_{10}\ p_{10}^{\text{avail}} + k_{10}^{\text{off}} e_{10}^m \\
&\quad\ +k^{\text{cat}}_{z_{10}:e_7^m}[Z_{10} : E_7^m] + (k^{\text{cat}}_{z_7:e_{10}} + k^-_{z_7:e_{10}})[Z_7 : E_{10}] - k^+_{z7:e10} z_7 e_{10} \\
&\quad\ +(k^{\text{cat}}_{z_7^m:e_{10}} + k^-_{z_7^m:e_{10}})[Z_7^m : E_{10}] - k^+_{z_m^7:e_{10}} z_7^m e_{10} \\
&\quad\ -k^+_{TFPI:e_{10}} e_{10}[TFPI] + k^-_{TFPI:e_{10}}[TFPI : E_{10}] - k^{\text{in}}_{AT:e_{10}} e_{10}
\end{aligned}$$

$$\begin{aligned}
\frac{d}{dt}\ z_{10}^m \quad &= \quad k_{10}^{\text{on}} z_{10}\ p_{10}^{\text{avail}} - k_{10}^{\text{off}} z_{10}^m - k^+_{z_{10}^m:ten} z_{10}^m [TEN] + k^-_{z_{10}:ten}[Z_{10}^m : TEN] \\
&\quad\ -k^+_{z_{10}^m:ten} z_{10}^m [TEN^*] + k^-_{z_{10}:ten}[Z_{10}^m : TEN^*]
\end{aligned}$$

$$\begin{aligned}
\frac{d}{dt}\ e_{10}^m \quad &= \quad k_{10}^{\text{on}} e_{10}\ p_{10}^{\text{avail}} - k_{10}^{\text{off}} e_{10}^m + k^{\text{cat}}_{z_{10}^m:ten}[Z_{10}^m : TEN] \\
&\quad\ +(k^{\text{cat}}_{z_5^m:e_{10}^m} + k^-_{z_5^m:e_{10}^m})[Z_5^m : E_{10}^m] - k^+_{z_5^m:e_{10}^m} z_5^m e_{10}^m \\
&\quad\ +(k^{\text{cat}}_{z_8^m:e_{10}^m} + k^-_{z_8^m:e_{10}^m})[Z_8^m : E_{10}^m] - k^+_{z_8^m:e_{10}^m} z_8^m e_{10}^m \\
&\quad\ +k^-_{e_5^m:e_{10}^m}[PRO] - k^+_{e_5^m:e_{10}^m} e_{10}^m e_5^m + k^{\text{cat}}_{z_{10}^m:ten}[Z_{10}^m : TEN^*]
\end{aligned}$$

$$\frac{d}{dt}\, z_5 = k_{\text{flow}}(z_5^{\text{up}} - z_5) - k_5^{\text{on}} z_5\; p_5^{\text{avail}} + k_5^{\text{off}} z_5^m - k^+_{z_5:e_2} z_5 e_2 + k^-_{z_5:e_2}[Z_5 : E_2]$$
$$+n_5\Big(k^+_{\text{adh}}\; p^{\text{avail}}_{PLAS} + k^{\text{act}}_{\text{plt}}\Big([PL^v_a] + [PL^s_a]\Big) + k^{\text{act}}_{\text{e2}}\frac{e_2}{(e_2 + 0.001)}\Big)[PL]$$

$$\frac{d}{dt}\, e_5 = k_{\text{flow}}(e_5^{\text{up}} - e_5) - k_5^{\text{on}} e_5\; p_5^{\text{avail}} + k_5^{\text{off}} e_5^m$$
$$+k^{\text{cat}}_{z_5:e_2}[Z_5 : E_2] + k^-_{e5:APC}[APC : E_5] - k^+_{e5:APC}[APC]e_5$$

$$\frac{d}{dt}\, z_5^m = k_5^{\text{on}} z_5\; p_5^{\text{avail}} - k_5^{\text{off}} z_5^m - k^+_{z_5^m:e_{10}^m} z_5^m e_{10}^m + k^-_{z_5^m:e_{10}^m}[Z_5^m : E_{10}^m]$$
$$-k^+_{z_5^m:e_2^m} z_5^m e_2^m + k^-_{z_5^m:e_2^m}[Z_5^m : E_2^m]$$

$$\frac{d}{dt}\, e_5^m = k_5^{\text{on}} e_5\; p_5^{\text{avail}} - k_5^{\text{off}} e_5^m + k^{\text{cat}}_{z_5^m:e_{10}^m}[Z_5^m : E_{10}^m] + k^{\text{cat}}_{z_5^m:e_2^m}[Z_5^m : E_2^m]$$
$$+k^-_{e_5^m:APC}[APC : E_5^m] - k^+_{e_5^m:APC}[APC]e_5^m$$
$$-k^+_{e_5^m:e_{10}^m} e_5^m e_{10}^m + k^-_{e_5^m:e_{10}^m}[PRO]$$

$$\frac{d}{dt}\, z_8 = k_{\text{flow}}(z_8^{\text{up}} - z_8) - k_8^{\text{on}} z_8\; p_8^{\text{avail}} + k_8^{\text{off}} z_8^m - k^+_{z_8:e_2} z_8 e_2 + k^-_{z_8:e_2}[Z_8 : E_2]$$

$$\frac{d}{dt}\, e_8 = k_{\text{flow}}(e_8^{\text{up}} - e_8) - k_8^{\text{on}} e_8\; p_8^{\text{avail}} + k_8^{\text{off}} e_8^m + k^{\text{cat}}_{z_8:e_2}[Z_8 : E_2] - 0.005 e_8$$
$$+k^-_{e_8:APC}[APC : E_8] - k^+_{e_8:APC}[APC]e_8$$

$$\frac{d}{dt}\, z_8^m = k_8^{\text{on}} z_8\; p_8^{\text{avail}} - k_8^{\text{off}} z_8^m - k^+_{z_8^m:e_{10}^m} z_8^m e_{10}^m + k^-_{z_8^m:e_{10}^m}[Z_8^m : E_{10}^m]$$
$$-k^+_{z_8^m:e_2^m} z_8^m e_2^m + k^-_{z_8^m:e_2^m}[Z_8^m : E_2^m]$$

$$\frac{d}{dt}\, e_8^m = k_8^{\text{on}} e_8\; p_8^{\text{avail}} - k_8^{\text{off}} e_8^m + k^{\text{cat}}_{z_8^m:e_{10}^m}[Z_8^m : E_{10}^m] + k^{\text{cat}}_{z_8^m:e_2^m}[Z_8^m : E_2^m]$$
$$-k^+_{e_8^m:APC}[APC]e_8^m + k^-_{e_8^m:APC}[APC : E_8^m] - 0.005 e_8^m$$
$$-k^+_{e_8^m:e_9^m} e_9^m e_8^m + k^-_{e_8^m:e_9^m}[TEN] - k^+_{e_8^m:e_9^m} e_8^m e_9^{m*} + k^-_{e_8^m:e_9^m}[TEN^*]$$

$$
\begin{aligned}
\frac{d}{dt}\, z_9 &= k_{\text{flow}}(z_9^{\text{up}} - z_9) - k_9^{\text{on}}\; p_9^{\text{avail}} z_9 + k_9^{\text{off}} z_9^m - k^+_{z_9:e_7^m} z_9 e_7^m + k^-_{z_9:e_7^m}[Z_9 : E_7^m] \\
&\quad - k^+_{z_9:e_{11}^h} z_9 e_{11}^h + k^-_{z_9:e_{11}^h}[Z_9 : E_{11}^h] - k^+_{z_9:e_{11}} z_9 e_{11} + k^-_{z_9:e_{11}}[Z_9 : E_{11}]
\end{aligned}
$$

$$
\begin{aligned}
\frac{d}{dt}\, e_9 &= k_{\text{flow}}(e_9^{\text{up}} - e_9) - k_{\text{diff}}(e_9 - e_9^{ec}) - k_9^{\text{on}}\; p_9^{\text{avail}} e_9 + k_9^{\text{off}} e_9^m + k^{\text{cat}}_{z_9:e_7^m}[Z_9 : E_7^m] \\
&\quad - k^{\text{in}}_{AT:e_9} e_9 + (k^{\text{cat}}_{z7:e9} + k^-_{z_7:e_9})[Z_7 : E_9] - k^+_{z_7:e_9} z_7 e_9 \\
&\quad + (k^{\text{cat}}_{z_7^m:e_9} + k^-_{z_7^m:e_9})[Z_7^m : E_9] - k^+_{z_7^m:e_9} z_7^m e_9 \\
&\quad - k_9^{\text{on}}\; p_9^{*,\text{avail}} e_9 + k_9^{\text{off}} e_9^{m*} + k^{\text{cat}}_{z_9:e_{11}^h}[Z_9 : E_{11}^h] + k^{\text{cat}}_{z_9:e_{11}}[Z_9 : E_{11}]
\end{aligned}
$$

$$
\begin{aligned}
\frac{d}{dt}\, z_9^m &= k_9^{\text{on}}\; p_9^{\text{avail}} z_9 - k_9^{\text{off}} z_9^m - k^+_{z_9^m:e_{11}^{h,m}} z_9^m e_{11}^{h,m} + k^-_{z_9^m:e_{11}^{h,m}}[Z_9^m : E_{11}^{h,m}] \\
&\quad - k^+_{z_9^m:e_{11}^{m*}} z_9^m e_{11}^{m*} + k^-_{z_9^m:e_{11}^{m*}}[Z_9^m : E_{11}^{m*}]
\end{aligned}
$$

$$
\begin{aligned}
\frac{d}{dt}\, e_9^m &= k_9^{\text{on}}\; p_9^{\text{avail}} e_9 - k_9^{\text{off}} e_9^m + k^-_{e_8^m:e_9^m}[TEN] - k^+_{e_8^m:e_9^m} e_8^m e_9^m \\
&\quad + k^{\text{cat}}_{z_9^m:e_{11}^{h,m}}[Z_9^m : E_{11}^{h,m}] + k^{\text{cat}}_{z_9^m:e_{11}^{m*}}[Z_9^m : E_{11}^{m*}]
\end{aligned}
$$

$$
\frac{d}{dt}\, z_2 = k_{\text{flow}}(z_2^{\text{up}} - z_2) - k_2^{\text{on}}\; p_2^{\text{avail}} z_2 + k_2^{\text{off}} z_2^m
$$

$$
\begin{aligned}
\frac{d}{dt}\, e_2 &= k_{\text{flow}}(e_2^{\text{up}} - e_2) - k_{\text{diff}}(e_2 - e_2^{ec}) + k^{\text{cat}}_{z2m:PRO}[Z_2^m : PRO] \\
&\quad - k_{2*}^{\text{on}}\; p_2^{*,\text{avail}} e_2 + k_{2*}^{\text{off}} e_2^m - k^{\text{in}}_{AT:e_2} e_2 \\
&\quad + (k^{\text{cat}}_{z_5:e_2} + k^-_{z_5:e_2})[Z_5 : E_2] - k^+_{z_5:e_2} z_5 e_2 \\
&\quad + (k^{\text{cat}}_{z_8:e_2} + k^-_{z_8:e_2})[Z_8 : E_2] - k^+_{z_8:e_2} z_8 e_2 \\
&\quad + (k^{\text{cat}}_{z_7:e_2} + k^-_{z_7:e_2})[Z_7 : E_2] - k^+_{z_7:e_2} z_7 e_2 \\
&\quad + (k^{\text{cat}}_{z_7^m:e_2} + k^-_{z_7^m:e_2})[Z_7^m : E_2] - k^+_{z_7^m:e_2} z_7^m e_2 \\
&\quad - k^+_{z_{11}:e_2} z_{11} e_2 + (k^-_{z_{11}:e_2} + k^{\text{cat}}_{z_{11}:e_2})[Z_{11} : E_2] \\
&\quad - k^+_{e_{11}^h:e_2} e_{11}^h e_2 + (k^-_{e_{11}^h:e_2} + k^{\text{cat}}_{e_{11}^h:e_2})[E_{11}^h : E_2]
\end{aligned}
$$

$$
\frac{d}{dt}\, z_2^m = k_2^{\text{on}}\; p_2^{\text{avail}} z_2 - k_2^{\text{off}} z_2^m - k^+_{z_2^m:PRO} z_2^m PRO + k^-_{z_2^m:PRO}[Z_z^m : PRO]
$$

$$
\begin{aligned}
\frac{d}{dt}\, e_2^m &= k_{2*}^{\text{on}}\; p_2^{\text{avail}} e_2 - k_{2*}^{\text{off}} e_2^m + (k^{\text{cat}}_{z_5^m:e_2^m} + k^-_{z_5^m:e_2^m})[Z_5^m : E_2^m] - k^+_{z_5^m:e_2^m} z_5^m e_2^m \\
&\quad + (k^{\text{cat}}_{z_8^m:e_2^m} + k^-_{z_8^m:e_2^m})[Z_8^m : E_2^m] - k^+_{z_8^m:e_2^m} z_8^m e_2^m \\
&\quad - k^+_{z_{11}^m:e_2^m} z_{11}^m e_2^m + (k^-_{z_{11}^m:e_2^m} + k^{\text{cat}}_{z_{11}^m:e_2^m})[Z_{11}^m : E_2^m] \\
&\quad - k^+_{e_{11}^{h,m*}:e_2^m} e_{11}^{h,m*} e_2^m + (k^-_{e_{11}^{h,m*}:e_2^m} + k^{\text{cat}}_{e_{11}^{h,m*}:e_2^m})[E_{11}^{h,m*} : E_2^m]
\end{aligned}
$$

$$
\begin{aligned}
\frac{d}{dt}\,[TEN] &= -k^-_{e_8^m:e_9^m}[TEN] + k^+_{e_8^m:e_9^m} e_8^m e_9^m \\
&\quad + (k^{\text{cat}}_{z_{10}^m:TEN} + k^-_{z_{10}:TEN})[Z_{10}^m : TEN] - k^+_{z_{10}^m:TEN} z_{10}^m [TEN]
\end{aligned}
$$

$$
\begin{aligned}
\frac{d}{dt}\,[PRO] &= -k^-_{e_5^m:e_{10}^m}[PRO] + k^+_{e_5^m:e_{10}^m} e_{10}^m e_5^m \\
&\quad + (k^{\text{cat}}_{z_2^m:PRO} + k^-_{z_2^m:PRO})[Z_2^m : PRO] - k^+_{z_2^m:PRO} z_2^m [PRO]
\end{aligned}
$$

$$
\frac{d}{dt}\,[PL_a^s] = k^+_{\text{adh}}\; p^{\text{avail}}_{PLAS}[PL] - k^-_{\text{adh}}[PL_a^s] + k^+_{\text{adh}}[PL_a^v]\; p^{\text{avail}}_{PLAS}
$$

$$
\begin{aligned}
\frac{d}{dt}\,[PL] &= k^p_{\text{flow}}\Big([PL]^{\text{up}} - [PL]\Big) - \Big(k^+_{\text{adh}}\; p^{\text{avail}}_{PLAS} + k^{\text{act}}_{\text{plt}}\Big([PL_a^v] + [PL_a^s]\Big) \\
&\quad + k^{\text{act}}_{\text{e2}} \frac{e_2}{e_2 + 0.001}\Big)[PL]
\end{aligned}
$$

$$
\begin{aligned}
\frac{d}{dt}\,[PL_a^v] &= k^-_{\text{adh}}[PL_a^s] - k^+_{\text{adh}}[PL_a^v]\; p^{\text{avail}}_{PLAS} \\
&\quad + \Big(k^{\text{act}}_{\text{plt}}\Big([PL_a^v] + [PL_a^s]\Big) + k^{\text{act}}_{\text{e2}} \frac{e_2}{e_2 + 0.001}\Big)[PL]
\end{aligned}
$$

$$
\begin{aligned}
\frac{d}{dt}\,[TFPI] &= k_{\text{flow}}([TPFI]^{\text{up}} - [TFPI]) - k^+_{TFPI:e_{10}} e_{10}[TFPI] \\
&\quad + k^-_{TFPI:e_{10}}[TFPI : Xa]
\end{aligned}
$$

$$
\begin{aligned}
\frac{d}{dt}\,[TFPI : E_{10}] &= -k_{\text{flow}}[TFPI : E_{10}] + k^+_{TFPI:e_{10}} e_{10}[TFPI] \\
&\quad - k^-_{TFPI:e_{10}}[TFPI : E_{10}] + k^-_{TFPI:e_{10}:e_7^m}[TFPI : E_{10} : E_7^m] \\
&\quad - k^+_{TFPI:e_{10}:e_7^m} e_7^m [TFPI : E_{10}]
\end{aligned}
$$

$$
\begin{aligned}
\frac{d}{dt}\,[TFPI : E_{10} : E_7^m] &= -k^-_{TFPI:e_{10}:e_7^m}[TFPI : E_{10} : E_7^m] \\
&\quad + k^+_{TFPI:e_{10}:e_7^m} e_7^m [TFPI : E_{10}] \\
&\quad - [TFPI : E_{10} : E_7^m] \frac{d}{dt}\,[PL_a^s] \frac{1}{p^{\text{avail}}_{PLAS}}
\end{aligned}
$$

$$
\begin{aligned}
\frac{d}{dt}\,[APC] &= k_{\text{flow}}\Big([APC]^{\text{up}} - [APC]\Big) - k_{\text{diff}}\Big([APC] - [APC^{ec}]\Big) \\
&+(k^{\text{cat}}_{e_5^m:APC} + k^{-}_{e_5^m:APC})[APC:E_5^m] - k^{+}_{e_5^m:APC}\; e_5^m[APC] \\
&+(k^{\text{cat}}_{e_8^m:APC} + k^{-}_{e_8^m:APC})[APC:E_8^m] - k^{+}_{e_8^m:APC}\; e_8^m[APC] \\
&+(k^{\text{cat}}_{e_5:APC} + k^{-}_{e_5:APC})[APC:E_5] - k^{+}_{e_5:APC}\; e_5[APC] \\
&+(k^{\text{cat}}_{e_8:APC} + k^{-}_{e_8:APC})[APC:E_8] - k^{+}_{e_8:APC}\; e_8[APC]
\end{aligned}
$$

$$
\frac{d}{dt}\,[APC:E_8^m] = k^{+}_{e_8^m:APC}\; e_8^m[APC] - (k^{\text{cat}}_{e_8^m:APC} + k^{-}_{e_8^m:APC})[APC:E_8^m]
$$

$$
\frac{d}{dt}\,[APC:E_5^m] = k^{+}_{e_5^m:APC}\; e_5^m[APC] - (k^{\text{cat}}_{e_5^m:APC} + k^{-}_{e_5^m:APC})[APC:E_5^m]
$$

$$
\frac{d}{dt}\,[APC:E_5] = k^{+}_{e_5:APC}\; e_5[APC] - (k^{\text{cat}}_{e_5:APC} + k^{-}_{e_5:APC})[APC:E_5]
$$

$$
\frac{d}{dt}\,[APC:E_8] = k^{+}_{e_8:APC}\; e_8[APC] - (k^{\text{cat}}_{e_8:APC} + k^{-}_{e_8:APC})[APC:E_8]
$$

$$
\begin{aligned}
\frac{d}{dt}\,[Z_7:E_2] &= k_{\text{flow}}\Big([Z_7:E_2]^{\text{up}} - [Z_7:E_2]\Big) + k^{+}_{z_7:e_2} z_7 e_2 \\
&-(k^{\text{cat}}_{z_7:e_2} + k^{-}_{z_7:e_2})[Z_7:E_2]
\end{aligned}
$$

$$
\begin{aligned}
\frac{d}{dt}\,[Z_7:E_{10}] &= k_{\text{flow}}\Big([Z_7:E_{10}]^{\text{up}} - [Z_7:E_{10}]\Big) + k^{+}_{z_7:e_{10}} z_7 e_{10} \\
&-(k^{\text{cat}}_{z_7:e_{10}} + k^{-}_{z_7:e_{10}})[Z_7:E_{10}]
\end{aligned}
$$

$$
\begin{aligned}
\frac{d}{dt}\,[Z_7^m:E_{10}] &= k^{+}_{z_7^m:e_{10}} z_7^m e_{10} - (k^{\text{cat}}_{z_7^m:e_{10}} + k^{-}_{z_7^m:e_{10}})[Z_7^m:E_{10}] \\
&-[Z_7^m:E_{10}]\frac{d}{dt}\,[PL_a^s]\frac{1}{p^{\text{avail}}_{PLAS}}
\end{aligned}
$$

$$
\begin{aligned}
\frac{d}{dt}\,[Z_7^m:E_2] &= k^{+}_{z_7^m:e_2} z_7^m e_2 - (k^{\text{cat}}_{z_7^m:e_2} + k^{-}_{z_7^m:e_2})[Z_7^m:E_2] \\
&-[Z_7^m:E_2]\frac{d}{dt}\,[PL_a^s]\frac{1}{p^{\text{avail}}_{PLAS}}
\end{aligned}
$$

$$
\begin{aligned}
\frac{d}{dt}\,[Z_{10}:E_7^m] &= k^{+}_{z_{10}:e_7^m} z_{10} e_7^m - (k^{\text{cat}}_{z_{10}:e_7^m} + k^{-}_{z_{10}:e_7^m})[Z_{10}:E_7^m] \\
&-[Z_{10}:E_7^m]\frac{d}{dt}\,[PL_a^s]\frac{1}{p^{\text{avail}}_{PLAS}}
\end{aligned}
$$

$$\frac{d}{dt}\,[Z_{10}^m : TEN] = k^+_{z_{10}^m:TEN} z_{10}^m [TEN] - (k^{\text{cat}}_{z_{10}^m:TEN} + k^-_{z_{10}:TEN})[Z_{10}^m : TEN]$$

$$\frac{d}{dt}\,[Z_5 : E_2] = k_{\text{flow}}\Big([Z_5 : E_2]^{\text{up}} - [Z_5 : E_2]\Big) + k^+_{z_5:e_2} z_5 e_2 - (k^{\text{cat}}_{z_5:e_2} + k^-_{z_5:e_2})[Z_5 : E_2]$$

$$\frac{d}{dt}\,[Z_5^m : E_{10}^m] = k^+_{z_5^m:e_{10}^m} z_5^m e_{10}^m - (k^{\text{cat}}_{z_5^m:e_{10}^m} + k^-_{z_5^m:e_{10}^m})[Z_5^m : E_{10}^m]$$

$$\frac{d}{dt}\,[Z_5^m : E_2^m] = k^+_{z_5^m:e_2^m} z_5^m e_2^m - (k^{\text{cat}}_{z_5^m:e_2^m} + k^-_{z_5^m:e_2^m})[Z_5^m : E_2^m]$$

$$\frac{d}{dt}\,[Z_8^m : E_{10}^m] = k^+_{z_8^m:e_{10}^m} z_8^m e_{10}^m - (k^{\text{cat}}_{z_8^m:e_{10}^m} + k^-_{z_8^m:e_{10}^m})[Z_8^m : E_{10}^m]$$

$$\frac{d}{dt}\,[Z_8^m : E_2^m] = k^+_{z_8^m:e_2^m} z_8^m e_2^m - (k^{\text{cat}}_{z_8^m:e_2^m} + k^-_{z_8^m:e_2^m})[Z_8^m : E_2^m]$$

$$\frac{d}{dt}\,[Z_8 : E_2] = k_{\text{flow}}\Big([Z_8 : E_2]^{\text{up}} - [Z_8 : E_2]\Big) + k^+_{z_8:e_2} z_8 e_2 - (k^{\text{cat}}_{z_8:e_2} + k^-_{z_8:e_2})[Z_8 : E_2]$$

$$\frac{d}{dt}\,[Z_9 : E_7^m] = k^+_{z_9:e_7^m} z_9 e_7^m - (k^{\text{cat}}_{z_9:e_7^m} + k^-_{z_9:e_7^m})[Z_9 : E_7^m] - [Z_9 : E_7^m]\frac{d}{dt}\,[PL_a^s]\frac{1}{p^{\text{avail}}_{PLAS}}$$

$$\frac{d}{dt}\,[Z_2^m : PRO] = k^+_{z_2^m:PRO} z_2^m [PRO] - (k^{\text{cat}}_{z_2^m:PRO} + k^-_{z_2^m:PRO})[Z_2^m : PRO]$$

$$\frac{d}{dt}\,[TF] = -[TF]\frac{d}{dt}\,[PL_a^s]\frac{1}{p^{\text{avail}}_{PLAS}}$$

$$\frac{d}{dt}\,e_9^{m*} = k_9^{\text{on}}\ p_9^{*,\text{avail}} e_9 - k_9^{\text{off}} e_9^{m*} + k^-_{e_8^m:e_9^m}[TEN^*] - k^+_{e_8^m:e_9^m} e_8^m e_9^{m*}$$

$$\frac{d}{dt}\,[TEN^*] = -k^-_{e_8^m:e_9^m}[TEN^*] + k^+_{e_8^m:e_9^m} e_8^m e_9^{m*} + (k^{\text{cat}}_{z_{10}^m:TEN} + k^-_{z_{10}:TEN})[Z_{10}^m : TEN^*] - k^+_{z_{10}^m:TEN} z_{10}^m [TEN^*]$$

$$\frac{d}{dt}\,[Z_{10}^m : TEN^*] = k^+_{z_{10}^m:TEN} z_{10}^m [TEN^*] - (k^{\text{cat}}_{z_{10}^m:TEN} + k^-_{z_{10}:TEN})[Z_{10}^m : TEN^*]$$

$$
\begin{aligned}
\frac{d}{dt}\, e_2^{ec} &= k_{\text{flow}}(e_2^{\text{up}} - e_2^{ec}) + k_{\text{diff}}(e_2 - e_2^{ec}) - -k_{AT:e_2}^{\text{in}} e_2^{ec} \\
&\quad -k_{TM}^{\text{on}} e_2^{ec} [TM]^{\text{avail}} + k_{TM}^{\text{off}} [TM : E_2^{ec}] \\
\frac{d}{dt}\, [APC^{ec}] &= k_{\text{flow}} \Big( [APC]^{\text{up}} - [APC^{ec}] \Big) + k_{\text{diff}}([APC] - [APC^{ec}]) \\
&\quad +k_{PC:TM:e_2^{ec}}^{\text{cat}} [TM : E_2^{ec} : APC] \\
\frac{d}{dt}\, [TM : E_2^{ec}] &= k_{TM}^{\text{on}} e_2^{ec} [TM]^{\text{avail}} - k_{TM}^{\text{off}} [TM : E_2^{ec}] - k_{PC:TM:e_2^{ec}}^{+} [TM : E_2^{ec}] \\
&\quad +(k_{PC:TM:e_2^{ec}}^{-} + k_{PC:TM:e_2^{ec}}^{\text{cat}}) [TM : E_2^{ec} : APC] \\
\frac{d}{dt}\, [TM : E_2^{ec} : APC] &= k_{PC:TM:e_2^{ec}}^{+} [TM : E_2^{ec}] \\
&\quad -(k_{PC:TM:e_2^{ec}}^{-} + k_{PC:TM:e_2^{ec}}^{\text{cat}}) [TM : E_2^{ec} : APC] \\
\frac{d}{dt}\, e_9^{ec} &= k_{\text{flow}}(e_9^{\text{up}} - e_9^{ec}) + k_{\text{diff}}(e_9 - e_9^{ec}) - k_{AT:e_9}^{\text{in}} e_9^{ec} \\
\frac{d}{dt}\, e_{10}^{ec} &= k_{\text{flow}}(e_{10}^{\text{up}} - e_{10}^{ec}) + k_{\text{diff}}(e_{10} - e_{10}^{ec}) - k_{AT:e_{10}}^{\text{in}} e_{10}^{ec} \\
\frac{d}{dt}\, z_{11} &= k_{\text{flow}}(z_{11}^{\text{up}} - z_{11}) - k_{z_{11}}^{\text{on}} z_{11}\; p_{11}^{\text{avail}} + k_{z_{11}}^{\text{off}} z_{11}^{m} - k_{z_{11}:e_2}^{+} z_{11} e_2 \\
&\quad +k_{z_{11}:e_2}^{-} [Z_{11} : E_2] \\
\frac{d}{dt}\, e_{11}^{h} &= k_{\text{flow}}(e_{11}^{h,\text{up}} - e_{11}^{h}) - k_{e_{11}^h}^{\text{on}*} e_{11}^{h}\; p_{11}^{*,\text{avail}} + k_{e_{11}^h}^{\text{off}*} e_{11}^{h,m*} \\
&\quad -k_{e_{11}^h}^{\text{on}} e_{11}^{h}\; p_{11}^{\text{avail}} + k_{e_{11}^h}^{\text{off}} e_{11}^{h,m} - k_{z_9:e_{11}^h}^{+} z_9 e_{11}^{h} \\
&\quad +(k_{z_9:e_{11}^h}^{-} + k_{z_9:e_{11}^h}^{\text{cat}}) [Z_9 : E_{11}^{h}] + k_{z_{11}:e_2}^{\text{cat}} [Z_{11} : E_2] \\
&\quad -k_{e_{11}^h:e_2}^{+} e_{11}^{h} e_2 + k_{e_{11}^h:e_2}^{-} [E_{11}^{h} : E_2] - k_{AT:e_{11}}^{\text{in}} e_{11}^{h} \\
\frac{d}{dt}\, e_{11} &= k_{\text{flow}}(e_{11}^{\text{up}} - e_{11}) - k_{e_{11}}^{\text{on}*} e_{11}\; p_{11}^{*,\text{avail}} + k_{e_{11}}^{\text{off}*} e_{11}^{m*} \\
&\quad -k_{z_9:e_{11}}^{+} z_9 e_{11} + (k_{z_9:e_{11}}^{-} + k_{z_9:e_{11}}^{\text{cat}}) [Z_9 : E_{11}] \\
&\quad +k_{e_{11}^h:e_2}^{\text{cat}} [E_{11}^{h} : E_2] - k_{AT:e_{11}}^{\text{in}} e_{11}
\end{aligned}
$$

$$\frac{d}{dt}\ z_{11}^{m} = k_{z_{11}}^{\text{on}} z_{11}\ p_{11}^{\text{avail}} - k_{z_{11}}^{\text{off}} z_{11}^{m} - k_{z_{11}^{m}:e_{2}^{m}}^{+} z_{11}^{m} e_{2}^{m} + k_{z_{11}^{m}:e_{2}^{m}}^{-} [Z_{11}^{m} : E_{2}^{m}]$$

$$\frac{d}{dt}\ e_{11}^{h,m} = k_{e_{11}^{h}}^{\text{on}} e_{11}^{h}\ p_{11}^{\text{avail}} - k_{e_{11}^{h}}^{\text{off}} e_{11}^{h,m} + k_{z_{11}^{m}:e_{2}^{m}}^{\text{cat}} [Z_{11}^{m} : E_{2}^{m}] \\ - k_{z_{9}^{m}:e_{11}^{h,m}}^{+} z_{9}^{m} e_{11}^{h,m} + (k_{z_{9}^{m}:e_{11}^{h,m}}^{-} + k_{z_{9}^{m}:e_{11}^{h,m}}^{\text{cat}})[Z_{9}^{m} : E_{11}^{h,m]}]$$

$$\frac{d}{dt}\ e_{11}^{h,m*} = k_{e_{11}^{h}}^{\text{on}*} e_{11}^{h}\ p_{11}^{*,\text{avail}} - k_{e_{11}^{h}}^{\text{off}*} e_{11}^{h,m*} \\ - k_{e_{11}^{h,m*}:e_{2}^{m}}^{+} e_{11}^{h,m*} e_{2}^{m} + k_{e_{11}^{h,m*}:e_{2}^{m}}^{-} [E_{11}^{h,m*} : E_{2}^{m}]$$

$$\frac{d}{dt}\ e_{11}^{m*} = k_{e_{11}}^{\text{on}*} e_{11}\ p_{11}^{*,\text{avail}} - k_{e_{11}}^{\text{off}*} e_{11}^{m*} + k_{e_{11}^{h,m*}:e_{2}^{m}}^{\text{cat}} [E_{11}^{h,m*} : E_{2}^{m}] \\ - k_{z_{9}^{m}:e_{11}^{m*}}^{+} z_{9}^{m} e_{11}^{m*} + (k_{z_{9}^{m}:e_{11}^{m*}}^{-} + k_{z_{9}^{m}:e_{11}^{m*}}^{\text{cat}})[Z_{9}^{m} : E_{11}^{m*}]$$

$$\frac{d}{dt}\ [Z_{9} : E_{11}^{h}] = k_{\text{flow}}\Big([Z_{9} : E_{11}^{h}]^{\text{up}} - [Z_{9} : E_{11}^{h}]\Big) + k_{z_{9}:e_{11}^{h}}^{+} z_{9} e_{11}^{h} \\ - (k_{z_{9}:e_{11}^{h}}^{-} + k_{z_{9}:e_{11}^{h}}^{\text{cat}})[Z_{9} : E_{11}^{h}]$$

$$\frac{d}{dt}\ [Z_{9} : E_{11}] = k_{\text{flow}\left([\mathrm{Z_9:E_{11}}]^{\mathrm{up}} - [\mathrm{Z_9:E_{11}}]\right)} + k_{z_{9}:e_{11}}^{+} z_{9} e_{11} \\ - (k_{z_{9}:e_{11}}^{-} + k_{z_{9}:e_{11}}^{\text{cat}})[Z_{9} : E_{11}]$$

$$\frac{d}{dt}\ [Z_{9}^{m} : E_{11}^{h,m}] = k_{z_{9}^{m}:e_{11}^{h,m}}^{+} z_{9}^{m} e_{11}^{h,m} - (k_{z_{9}^{m}:e_{11}^{h,m}}^{-} + k_{z_{9}^{m}:e_{11}^{h,m}}^{\text{cat}})[Z_{9}^{m} : E_{11}^{h,m}]$$

$$\frac{d}{dt}\ [Z_{9}^{m} : E_{11}^{m*}] = k_{z_{9}^{m}:e_{11}^{m*}}^{+} z_{9}^{m} e_{11}^{m*} - (k_{z_{9}^{m}:e_{11}^{m*}}^{-} + k_{z_{9}^{m}:e_{11}^{m*}}^{\text{cat}})[Z_{9}^{m} : E_{11}^{m*}]$$

$$\frac{d}{dt}\ [Z_{11} : E_{2}] = k_{\text{flow}}\Big([Z_{11} : E_{2}]^{\text{up}} - [Z_{11} : E_{2}]\Big) \\ + k_{z_{11}:e_{2}}^{+} z_{11} e_{2} - (k_{z_{11}:e_{2}}^{-} + k_{z_{11}:e_{2}}^{\text{cat}})[Z_{11} : E_{2}]$$

$$\frac{d}{dt}\ [E_{11}^{h} : E_{2}] = k_{\text{flow}}\Big([E_{11}^{h} : E_{2}]^{\text{up}} - [E_{11}^{h} : E_{2}]\Big) \\ + k_{e_{11}^{h}:e_{2}}^{+} e_{11}^{h} e_{2} - (k_{e_{11}^{h}:e_{2}}^{-} + k_{e_{11}^{h}:e_{2}}^{\text{cat}})[E_{11}^{h} : E_{2}]$$

$$\frac{d}{dt}\ [Z_{11}^{m} : E_{2}^{m}] = k_{z_{11}^{m}:e_{2}^{m}}^{+} z_{11}^{m} e_{2}^{m} - (k_{z_{11}^{m}:e_{2}^{m}}^{-} + k_{z_{11}^{m}:e_{2}^{m}}^{\text{cat}})[Z_{11}^{m} : E_{2}^{m}]$$

$$\frac{d}{dt}\ [E_{11}^{h,m*} : E_{2}^{m}] = k_{e_{11}^{h,m*}:e_{2}^{m}}^{+} e_{11}^{h,m*} e_{2}^{m} - (k_{e_{11}^{h,m*}:e_{2}^{m}}^{-} + k_{e_{11}^{h,m*}:e_{2}^{m}}^{\text{cat}})[E_{11}^{h,m*} : E_{2}^{m}]$$

**S1 Table. INITIAL PLASMA LEVELS.** Descriptions, notation and labels for each parameter associated with initial plasma levels are listed. The value of each parameter is found in the corresponding table listed above.

| Description | Notation | Label | Table |
|---|---|---|---|
| Prothrombin | $z_2$ | $Z_2$ | S5 |
| Factor V | $z_5$ | $Z_5$ | S5 |
| Factor VII | $z_7$ | $Z_7$ | S5 |
| Factor VIII | $z_8$ | $Z_8$ | S5 |
| Factor IX | $z_9$ | $Z_9$ | S5 |
| Factor X | $z_{10}$ | $Z_{10}$ | S5 |
| Factor XI | $z_{11}$ | $Z_{11}$ | S5 |
| TFPI | [TFPI] | TFPI | S5 |
| AT | $k^{in}_{AT:e_2}$, $k^{in}_{AT:e_9}$, $k^{in}_{AT:e_{10}}$, $k^{in}_{AT:e_{11}}$ | AT | S11 |

**S2 Table. PLATELET CHARACTERISTICS.** Descriptions, notation and labels for each parameter associated with platelet characteristics are listed. The value of each parameter is found in the corresponding table listed above.

| Description | Notation | Label | Table |
|---|---|---|---|
| Platelet count | $\mathrm{PL}^{up}$ | PLup | S5 |
| Binding site number for II | $N_2$ | N2 | S5 |
| Binding site number for IIa | $N_2^*$ | N2* | S5 |
| Binding site number for V/Va | $N_5$ | N5 | S5 |
| Binding site number for VIII/VIIIa | $N_8$ | N8 | S5 |
| Binding site number for IX | $N_9$ | N9 | S5 |
| Binding site number for IXa | $N_9^*$ | N9* | S5 |
| Binding site number for X/Xa | $N_{10}$ | N10 | S5 |
| Binding site number for XI | $N_{11}$ | N11 | S5 |
| Binding site number for XIa | $N_{11}^*$ | N11* | S5 |
| Rate of unactivated platelets adhering to SE | $k^{+}_{adh}$ | kadh | S12 |
| Rate of activated platelets adhering to SE | $k^{+,*}_{adh}$ | kadh1 | S12 |
| Rate of platelet activation by platelet in solution | $k^{act}_{plt}$ | $\mathrm{kact}_{plt}$ | S12 |
| Rate of platelet activation on SE | $k^{act,*}_{plt}$ | $\mathrm{kact*}_{plt}$ | S12 |
| Rate of platelet activation by thrombin | $k^{act}_{e_2}$ | $\mathrm{kact}_{e2}$ | S7 |

**S3 Table. KINETIC RATE CONSTANTS.** Descriptions, notation and labels for each parameter associated with kinetic rate constants are listed. The value of each parameter is found in the corresponding table listed above.

| Description | Notation | Label | Table |
|---|---|---|---|
| Rates of activation of TF:VII by fX | $K_M$ | KZ7mE10M | S7 |
| | $k^{cat}_{z_7^m:e_{10}}$ | KZ7mE10CAT | S7 |
| | $k^{-}_{z_7^m:e_{10}}$ | KZ7mE10MI | S7 |
| Rates of activation of fX by TF:VIIa | $K_M$ | KZ10E7mM | S7 |
| | $k^{cat}_{z_{10}:e_7^m}$ | KZ10E7mCAT | S7 |
| | $k^{-}_{z_{10}:e_7^m}$ | KZ10E7mMI | S7 |
| Rates of activation of fIX by TF:VIIa | $K_M$ | KZ9E7mM | S7 |
| | $k^{cat}_{z_9:e_7^m}$ | KZ9E7mCAT | S7 |
| | $k^{-}_{z_9:e_7^m}$ | KZ9E7mMI | S7 |
| Rates of binding of fVII/fVIIa to TF | $k_7^{on}$ | K7ON | S7 |
| | $k_7^{off}$ | K7OFF | S8 |
| Rates of activation of TF:VII by fXa | $K_M$ | KZ7E10M | S8 |
| | $k^{cat}_{z_7:e_{10}^m}$ | KZ7E10CAT | S8 |
| | $k^{-}_{z_7:e_{10}^m}$ | KZ7E10MI | S8 |
| Rates of activation of TF:VII by fIIa | $K_M$ | KZ7E2M | S8 |
| | $k^{cat}_{z_7:e_2}$ | KZ7E2CAT | S8 |
| | $k^{-}_{z_7:e_2}$ | KZ7E2MI | S8 |
| Rates of activation of TF:VII by fIXa | $K_M$ | KZ7E9M | S8 |
| | $k^{cat}_{z_7:e_9}$ | KZ7E9CAT | S8 |
| | $k^{-}_{z_7:e_9}$ | KZ7E9MI | S8 |
| Rates of activation of fV by fIIa | $K_M$ | KZ5E2M | S8 |
| | $k^{cat}_{z_5:e_2}$ | KZ5E2CAT | S8 |
| | $k^{-}_{z_5:e_2}$ | KZ5E2MI | S8 |
| Rates of activation of fVIII by fIIa | $K_M$ | KZ8E2M | S8 |
| | $k^{cat}_{z_8:e_2}$ | KZ8E2CAT | S8 |
| | $k^{-}_{z_8:e_2}$ | KZ8E2MI | S8 |
| Rates of activation of fIX by fXIa-fXIa | $k^{+}_{z_9:e_{11}}$ | KZ9E11P | S8 |
| | $k^{cat}_{z_9:e_{11}}$ | KZ9E11CAT | S8 |
| | $k^{-}_{z_9:e_{11}}$ | KZ9E11MI | S8 |
| Rates of activation of fIX by fXIa-fXI | $k^{+}_{z_9:e_{11}^h}$ | KZ9E11P | S8 |
| | $k^{cat}_{z_9:e_{11}^h}$ | KZ9E11CAT | S8 |
| | $k^{-}_{z_9:e_{11}^h}$ | KZ9E11MI | S8 |
| Rates of activation of fXI by fIIa | $k^{+}_{z_{11}:e_2}$ | KZ11E2P | S8 |
| | $k^{cat}_{z_{11}:e_2}$ | KZ11E2CAT | S8 |
| | $k^{-}_{z_{11}:e_2}$ | KZ11E2MI | S9 |
| Rates of binding of fX/fXa to plt. surface | $k_{10}^{on}$ | K10ON | S9 |
| | $k_{10}^{off}$ | K10OFF | S9 |
| Rates of binding of fV/fVa to plt. surface | $k_5^{on}$ | K5ON | S9 |
| | $k_5^{off}$ | K5OFF | S9 |
| Rates of binding of fVIII/fVIIIa to plt. surface | $k_8^{on}$ | K8ON | S9 |
| | $k_8^{off}$ | K8OFF | S9 |
| Rates of binding of fIX/fIXa to plt. surface | $k_9^{on}$ | K9ON | S9 |
| | $k_9^{off}$ | K9OFF | S9 |
| Rates of binding of fII/fIIa to plt. surface | $k_2^{on}$, $k_2^{on,*}$ | K2ON, K2SON | S9 |
| | $k_2^{off}$, $k_2^{off,*}$ | K2OFF, K2SOFF | S9 |
| Rates of binding of fXI/fXIa to plt. surface | $k_{11}^{on}$, $k_{11}^{on,*}$ | K11ON, K11SON | S9 |
| | $k_{11}^{off}$, $k_{11}^{off,*}$ | K11OFF, K11SOFF | S10 |

**S4 Table. KINETIC RATE CONSTANTS.** Descriptions, notation and labels for each parameter associated with kinetic rate constants are listed. The value of each parameter is found in the corresponding table listed above.

| **Description** | **Notation** | **Label** | **Table** |
|---|---|---|---|
| Rates of activation of fV by fXa on plt. surface | $K_M$ | KZ5mE10mM | S10 |
| | $k^{cat}_{z_5^m:e_{10}^m}$ | KZ5mE10mCAT | S10 |
| | $k^{-}_{z_5^m:e_{10}^m}$ | KZ5mE10mMI | S10 |
| Rates of activation of fV by fIIa on plt. surface | $K_M$ | KZ5mE2mM | S10 |
| | $k^{cat}_{z_5:e_2^m}$ | KZ5mE2mCAT | S10 |
| | $k^{-}_{z_5:e_2^m}$ | KZ5mE2mMI | S10 |
| Rates of activation of fVIII by fXa on plt. surface | $K_M$ | KZ8ME10MM | S10 |
| | $k^{cat}_{z_8:e_{10}^m}$ | KZ8ME10MCAT | S10 |
| | $k^{-}_{z_8:e_{10}^m}$ | KZ8ME10MMI | S10 |
| Rates of activation of fVIII by fIIa on plt. surface | $K_M$ | KZ8ME2MM | S10 |
| | $k^{cat}_{z_8:e_2^m}$ | KZ8mE2mCAT | S10 |
| | $k^{-}_{z_8:e_2^m}$ | KZ8mE2mMI | S10 |
| Rates of activation of fX by TEN on plt. surface | $K_M$ | KZ10mTENM | S10 |
| | $k^{cat}_{z_{10}^m:TEN}$ | KZ10mTENCAT | S10 |
| Rates of activation of fII by PRO on plt. surface | $K_M$ | KZ2mPROM | S10 |
| | $k^{cat}_{z_2^m:PRO}$ | KZ2mPROCAT | S10 |
| Rates of activation of fXI by fIIa on plt. surfaces | $k^{+}_{z_{11}^m:e_2^m}$ | KZ11mE2mP | S10 |
| | $k^{cat}_{z_{11}^m:e_2^m}$ | KZ11mE2mCAT | S10 |
| | $k^{-}_{z_{11}^m:e_2^m}$ | KZ11mE2mMI | S10 |
| Rates of activation of fIX by fXIa-fXIa on plt. surface | $K_M$ | KZ9mE11mP | S10 |
| | $k^{cat}_{z_9^m:e_{11}^m}$ | KZ9mE11mCAT | S10 |
| | $k^{-}_{z_9^m:e_2^m}$ | KZ9mE11mMI | S10 |
| Rates of formation of TEN on plt. surface | $k^{+}_{e_8^m:e_9^m}$ | KE8mE9mP | S10 |
| | $k^{-}_{e_8^m:e_9^m}$ | KE8mE9mMI | S10 |
| Rates of formation of PRO on plt. surface | $k^{+}_{e_5^m:e_{10}^m}$ | KE5mE10mP | S10 |
| | $k^{-}_{e_5^m:e_{10}^m}$ | KE5mE10mMI | S10 |
| Rates of inhibition of fXa by TFPI | $k^{+}_{tfpia:e_{10}}$ | KTFPI_E10_P | S11 |
| | $k^{-}_{tfpia:e_{10}}$ | KTFPI_E10_M | S11 |
| Rates of inhibition of TF:VIIa by TFPIa | $k^{+}_{tfpia:e_7^m}$ | KTFPIa_E7m_P | S11 |
| | $k^{-}_{tfpia:e_7^m}$ | KTFPIa_E7m_M | S11 |
| Rates of inhibition of fVa by APC on plt. surface | $K_M$ | KE5mAPCM | S11 |
| | $k^{cat}_{e_5^m:APC}$ | KE5mAPCCAT | S11 |
| | $k^{-}_{e_5^m:APC}$ | KE5mAPCMI | S11 |
| Rates of inhibition of fVIIIa by APC on plt. surface | $K_M$ | KE8mAPCM | S11 |
| | $k^{cat}_{e_8^m:APC}$ | KE8mAPCCAT | S11 |
| | $k^{-}_{e_8^m:APC}$ | KE8mAPCMI | S11 |
| Rates of inhibition of fIIa by TM on plt. surface | $k^{on}_{TM}$ | KTMP | S11 |
| | $k^{off}_{TM}$ | KTMM | S12 |

**Kinetic and Physical Parameter Values:**

**S5 Table. DIFFUSION COEFFICIENTS FOR PLATELETS AND FLUID-PHASE CHEMICAL SPECIES** (a) From [3]. (b) From [4].

| | | |
|---|---|---|
| Platelets | $2.5\times10^{-7}$ cm$^2$/s | a |
| Proteins | $5\times10^{-7}$ cm$^2$/s | b |

**S6 Table. NORMAL CONCENTRATIONS AND SURFACE BINDING SITE NUMBERS** (a) From [5]. (b) From [6]. (c) [7] suggests that normal plasma concentration of fVIIa is about 1% of the normal fVII concentration. (d) From [8]. (e) (f) From [9]. (g) Estimated as described in the text of the Supplementary Information. (h) From [10]. (i) From [11]. (j) From [12]. (k) From [13]. (l) From [14,15]. (m) Number of fV molecules released per activated platelet [16]. (n) Maximum concentration of platelets in a 2 $\mu$m high reaction zone assuming that 20 platelets can cover a 10$\mu$m-by-10$\mu$m injured surface [17].

| | | |
|---|---|---|
| Prothrombin | 1.4 $\mu$M | a |
| Factor V | 0.01 $\mu$M | b |
| Factor VII | 0.01 $\mu$M | a |
| Factor VIIa | 0.1 nM | c |
| Factor VIII | 1.0 nM | a |
| Factor IX | 0.09 $\mu$M | a |
| Factor X | 0.17 $\mu$M | a |
| Factor XI | 30.0 nM | a |
| TFPI | 2.5 nM | d |
| Protein C | 65 nM | e |
| Platelet count | $2.5(10)^5/\mu$l | f |
| $N_2$ | 1000/plt | g |
| $N_2^*$ | 1000/plt | g |
| $N_5$ | 3000/plt | h |
| $N_8$ | 450/plt | i |
| $N_9$ | 250/plt | j |
| $N_9^*$ | 250/plt | j |
| $N_{10}$ | 2700/plt | k |
| $N_{11}$ | 1500/plt | l |
| $N_{11}^*$ | 250/plt | l |
| $n_5$ | 3000/plt | m |
| $p_{PLAS}$ | 0.167 nM | n |

**S7 Table. REACTIONS ON SUBENDOTHELIUM** (a) $k^{\text{cat}}_{z_7^m:e_{10}} = 5.0$ sec$^{-1}$ and $K_M = 1.2 \cdot 10^{-6}$ M [18]. (b) $k^{\text{cat}}_{z_7^m:e_2} = 6.1 \cdot 10^{-2}$ sec$^{-1}$ and $K_M = 2.7 \cdot 10^{-6}$ M [18]. (d) $k^{\text{cat}}_{z_{10}:e_7^m} = 1.15$ sec$^{-1}$ and $K_M = 4.5 \cdot 10^{-7}$ M [5]. (d) $k^{\text{cat}}_{z_9:e_7^m} = 1.15$ sec$^{-1}$ and $K_M = 2.4 \cdot 10^{-7}$ M [19]. (e) $K_d = 1.0 \cdot 10^{-10}$ M [20].

| | | | | | | | |
|---|---|---|---|---|---|---|---|
| Activation (of -, by -) | | | | | | | |
| (TF:VII,fXa) | $E_{10}$, $Z_7^m$ | $Z_7^m : E_{10}$ | $E_7^m$ | $k^+_{z_7^m:e_{10}}$=$5.0 \cdot 10^6$ | $k^-_{z_7^m:e_{10}}$=1.0 | $k^{\text{cat}}_{z_7^m:e_{10}}$=5.0 | a |
| (TF:VII, fIIa) | $E_2$, $Z_7^m$ | $Z_7^m : E_2$ | $E_7^m$ | $k^+_{z_7^m:e_2}$=$3.92 \cdot 10^5$ | $k^-_{z_7^m:e_2}$=1.0 | $k^{\text{cat}}_{z_7^m:e_2}$=$6.1 \cdot 10^{-2}$ | b |
| (fX, TF:VIIa) | $E_7^m$, $Z_{10}$ | $Z_{10} : E_7^m$ | $E_{10}$ | $k^+_{z_{10}:e_7^m}$=$5.0 \cdot 10^6$ | $k^-_{z_{10}:e_7^m}$=1.0 | $k^{\text{cat}}_{z_{10}:e_7^m}$=1.15 | c |
| (fIX, TF:VIIa) | $E_7^m$, $Z_9$ | $Z_9 : E_7^m$ | $E_9$ | $k^+_{z_9:e_7^m}$=$9.4 \cdot 10^6$ | $k^-_{z_9:e_7^m}$=1.0 | $k^{\text{cat}}_{z_9:e_7^m}$=1.15 | d |
| Binding (of -, with -) | | | | | | | |
| (fVII, TF) | $Z_7$, $TF$ | | $Z_7^m$ | $k_7^{\text{on}}$=$5.0 \cdot 10^7$ | $k_7^{\text{off}}$=$5.0 \cdot 10^{-3}$ | | e |
| (fVIIa, TF) | $E_7$, $TF$ | | $E_7^m$ | $k_7^{\text{on}}$=$5.0 \cdot 10^7$ | $k_7^{\text{off}}$=$5.0 \cdot 10^{-3}$ | | e |

**S8 Table. REACTIONS IN THE PLASMA** (a) $k^{\text{cat}}_{z_7:e_{10}} = 5.0\ \text{sec}^{-1}$ and $K_M = 1.2 \cdot 10^{-6}$ M [18]. (b) $k^{\text{cat}}_{z_7:e_2} = 6.1 \cdot 10^{-2}\ \text{sec}^{-1}$ and $K_M = 2.7 \cdot 10^{-6}$ M [18] (c) $k^{\text{cat}}_{z_5:e_2} = 0.23\ \text{sec}^{-1}$ and $K_M = 7.17 \cdot 10^{-8}$ M [21]. (d) $k^{\text{cat}}_{z_8:e_2} = 0.9\ \text{sec}^{-1}$ [22] and $K_M = 2 \cdot 10^{-7}$ M [23]. (e) $k^{\text{cat}}_{z_{11}:e_2} = 1.3 \cdot 10^{-4}$, $K_M = 50$nM [24]. Rate constants apply also for thrombin-activation of XIa-XI. (f) $k^{\text{cat}}_{z_9:e^h_{11}} = 0.21$, $K_M = 0.2\mu$M [25, 26]. Rate constants apply also for activation of IX by XIa-XIa.

| Reaction | Reactants | Complex | Product | $\text{M}^{-1}\text{sec}^{-1}$ | $\text{sec}^{-1}$ | $\text{sec}^{-1}$ | Note |
|---|---|---|---|---|---|---|---|
| Activation (of -, by -) | | | | | | | |
| (fVII, fXa) | $Z_7$, $E_{10}$ | $Z_7 : E_{10}$ | $E_7$ | $k^+_{z_7:e_{10}}$=5 $\cdot\, 10^6$ | $k^-_{z_7:e_{10}}$=1.0 | $k^{\text{cat}}_{z_7:e_{10}}$=5.0 | a |
| (fVII, fIIa) | $Z_7$, $E_2$ | $Z_7 : E_2$ | $E_7$ | $k^+_{z_7:e_2}$=3.92 $\cdot\, 10^5$ | $k^-_{z_7:e_2}$=1.0 | $k^{\text{cat}}_{z_7:e_2}$=6.1 $\cdot\, 10^{-2}$ | b |
| (fV, fIIa) | $Z_5$, $E_2$ | $Z_5 : E_2$ | $E_5$ | $k^+_{z_5:e_2}$= 1.73 $\cdot\, 10^7$ | $k^-_{z_5:e_2}$=1.0 | $k^{\text{cat}}_{z_5:e_2}$=0.23 | c |
| (fVIII, fIIa) | $Z_8$, $E_2$ | $Z_8 : E_2$ | $E_8$ | $k^+_{z_8:e_2}$=2.64 $\cdot\, 10^7$ | $k^-_{z_8:e_2}$=1.0 | $k^{\text{cat}}_{z_8:e_2}$=0.9 | d |
| (fXI-fXI, fIIa) | $Z_{11}$, $E_2$ | $Z_{11} : E_2$ | $E^h_{11}$ | $k^+_{z_{11}:e_2} = 2.0 \cdot 10^7$ | $k^-_{z_{11}:e_2} = 1.0$ | $k^{\text{cat}}_{z_{11}:e_2} = 1.3 \cdot 10^{-4}$ | e |
| (fIX, fXIa) | $Z_9$, $E^h_{11}$ | $Z_9 : E^h_{11}$ | $E_9$ | $k^+_{z_9:e^h_{11}} = 0.6 \cdot (10)^7$ | $k^-_{z_9:e^h_{11}} = 1.0$ | $k^{\text{cat}}_{z_9:e^h_{11}} = 0.21$ | f |

**S9 Table. BINDING TO PLATELET SURFACES** (a) For fIX binding to platelets, $K_d = 2.5 \cdot 10^{-9}$ M [12], and for fX binding to platelets, $K_d$ has approximately the same value [10]. For fX binding to PCPS vesicles, the on-rate is about $10^7$ $\mathrm{M}^{-1}\mathrm{sec}^{-1}$ and the off-rate is about 1.0 $\mathrm{sec}^{-1}$ [27] giving a dissociation constant of about $10^{-7}$ M. To estimate on- and off-rates for the higher-affinity binding of fX to platelets, we keep the on-rate the same as for vesicles and adjust the off-rate to give the correct dissociation constant. The rates for fIX binding with platelets are taken to be the same as for fX binding. (b) We assume binding constants for fIXa binding to the specific fIXa binding sites are the same as for shared sites. (c) fV binds with high-affinity to phospholipids (PCPS) [27] and we use the same rate constants reported there to describe fV binding to platelets. (d) The $K_d$ for fVIII binding with platelets is taken from [11]. We set the off-rate $k_8^{\mathrm{off}}$ for fVIII binding to platelets equal to that for fV binding to platelets, and calculate the on-rate $k_8^{\mathrm{on}}$. (e) For prothrombin interactions with platelets, $K_d$ is reported to be $5.9 \cdot 10^{-7}$ M [28]. We choose $k_2^{\mathrm{off}}$ and set $k_2^{\mathrm{on}} = k_2^{\mathrm{off}}/K_d$. (f) Estimated as described in the text of the Supplementary Information. (g) $K_d$ = 10 nM [29]. (h) $K_d$ = 1.7 nM [15].

| Reaction | Reactants | Products | $\mathrm{M}^{-1}\mathrm{sec}^{-1}$ | $\mathrm{sec}^{-1}$ | Note |
|---|---|---|---|---|---|
| Factor IX | $Z_9$, $P_9$ | $Z_9^m$ | $k_9^{\mathrm{on}}$=$1.0 \cdot 10^7$ | $k_9^{\mathrm{off}}$=$2.5 \cdot 10^{-2}$ | a |
| Factor IXa | $E_9$, $P_9$ | $E_9^m$ | $k_9^{\mathrm{on}}$=$1.0 \cdot 10^7$ | $k_9^{\mathrm{off}}$=$2.5 \cdot 10^{-2}$ | a |
| Factor IXa | $E_9$, $P_9^*$ | $E_9^{m,*}$ | $k_9^{\mathrm{on}}$=$1.0 \cdot 10^7$ | $k_9^{\mathrm{off}}$=$2.5 \cdot 10^{-2}$ | b |
| Factor X | $Z_{10}$, $P_{10}$ | $Z_{10}^m$ | $k_{10}^{\mathrm{on}}$=$1.0 \cdot 10^7$ | $k_{10}^{\mathrm{off}}$=$2.5 \cdot 10^{-2}$ | a |
| Factor Xa | $E_{10}$, $P_{10}$ | $E_{10}^m$ | $k_{10}^{\mathrm{on}}$=$1.0 \cdot 10^7$ | $k_{10}^{\mathrm{off}}$=$2.5 \cdot 10^{-2}$ | a |
| Factor V | $Z_5$, $P_5$ | $Z_5^m$ | $k_5^{\mathrm{on}}$=$5.7 \cdot 10^7$ | $k_5^{\mathrm{off}}$=0.17 | c |
| Factor Va | $E_5$, $P_5$ | $E_5^m$ | $k_5^{\mathrm{on}} = 5.7 \cdot 10^7$ | $k_5^{\mathrm{off}}$=0.17 | c |
| Factor VIII | $Z_8$, $P_8$ | $Z_8^m$ | $k_8^{\mathrm{on}} = 5.0 \cdot 10^7$ | $k_8^{\mathrm{off}}$=0.17 | d |
| Factor VIIIa | $E_8$, $P_8$ | $E_8^m$ | $k_8^{\mathrm{on}} = 5.0 \cdot 10^7$ | $k_8^{\mathrm{off}}$=0.17 | d |
| Factor II | $Z_2$, $P_2$ | $Z_2^m$ | $k_2^{\mathrm{on}} = 1.0 \cdot 10^7$ | $k_2^{\mathrm{off}}$=5.9 | e |
| Factor IIa | $E_2$, $P_2$ | $E_2^m$ | $k_2^{*,\mathrm{on}} = 1.0 \cdot 10^7$ | $k_2^{*,\mathrm{off}} = 0.2$ | f |
| Factor XI | $Z_{11}$, $P_{11}$ | $Z_{11}^m$ | $k_{z_{11}}^{\mathrm{on}} = 1.0 \cdot 10^7$ | $k_{z_{11}}^{\mathrm{off}}$=0.1 | g |
| Factor XIa | $E_{11}$, $P_{11}^*$ | $E_{11}^m$ | $k_{e_{11}}^{\mathrm{on}} = 1.0 \cdot 10^7$ | $k_{e_{11}}^{\mathrm{off}}$=0.017 | h |

**S10 Table. REACTIONS ON PLATELET SURFACES** (a) $k^{\text{cat}}_{z_5^m:e_{10}^m} = 0.046\ \text{sec}^{-1}$ and $K_M = 10.4 \cdot 10^{-9}$ M [30]. (b) The rate constants for thrombin activation of fV on platelets are assumed to be the same as in plasma. (c) $k^{\text{cat}}_{z_8^m:e_{10}^m} = 0.023\ \text{sec}^{-1}$ and $K_M = 2.0 \cdot 10^{-8}$ M [23]. (d) The rate constants for thrombin activation of fVIII on platelets are assumed to be the same as in plasma. (e) The formation of the tenase and prothrombinase complexes is assumed to be very fast with $K_d = 1.0 \cdot 10^{-10}$ M [31]. (f) $k^{\text{cat}}_{z_{10}^m:ten} = 20\ \text{sec}^{-1}$ and $K_M = 1.6 \cdot 10^{-7}$ M [32]. (g) $k^{\text{cat}}_{z_2^m:pro} = 30\ \text{sec}^{-1}$ and $K_M = 3.0 \cdot 10^{-7}$ M [33]. (h) $k^{\text{cat}}_{z_{11}^m:e_2^m} = 1.3 \cdot 10^{-4}$, $K_M = 50$ nM [24]. Rate constants apply also for thrombin-activation of Plt-XIa-XI. (i) $k^{\text{cat}}_{z_9^m:e_{11}^{h,m}} = 0.21$, $K_M = 0.2\mu$M [25, 26]. Rate constants apply also for activation of platelet-bound IX by Plt-XIa-XIa.

| Reaction | Reactants | Complex | Product | $M^{-1}sec^{-1}$ | $sec^{-1}$ | $sec^{-1}$ | Note |
|---|---|---|---|---|---|---|---|
| Activation (of -, by -) | | | | | | | |
| (V, Xa) | $Z_5^m$, $E_{10}^m$ | $Z_5^m : E_{10}^m$ | $E_5^m$ | $k^+_{z_5^m:e_{10}^m}=1.0 \cdot 10^8$ | $k^-_{z_5^m:e_{10}^m}=1.0$ | $k^{\text{cat}}_{z_5^m:e_{10}^m}=4.6 \cdot 10^{-2}$ | a |
| (V, IIa) | $Z_5^m$, $E_2^m$ | $Z_5^m : E_2^m$ | $E_5^m$ | $k^+_{z_5^m:e_2^m}=1.73 \cdot 10^7$ | $k^-_{z_5^m:e_2^m}=1.0$ | $k^{\text{cat}}_{z_5^m:e_2^m}= 0.23$ | b |
| (VIII, Xa) | $Z_8^m$, $E_{10}^m$ | $Z_8^m : E_{10}^m$ | $E_8^m$ | $k^+_{z_8^m:e_{10}^m}=5.1 \cdot 10^7$ | $k^-_{z_8^m:e_{10}^m}=1.0$ | $k^{\text{cat}}_{z_8^m:e_{10}^m}=2.3 \cdot 10^{-2}$ | c |
| (VIII, IIa) | $Z_8^m$, $E_2^m$ | $Z_8^m : E_2^m$ | $E_8^m$ | $k^+_{z_8^m:e_2^m}=2.64 \cdot 10^7$ | $k^-_{z_8^m:e_2^m}=1.0$ | $k^{\text{cat}}_{z_8^m:e_2^m}= 0.9$ | d |
| (X, VIIIa:IXa) | $Z_{10}^m$, $TEN$ | $Z_{10}^m : TEN$ | $E_{10}^m$ | $k^+_{z_{10}^m:ten}=1.31 \cdot 10^8$ | $k^-_{z_{10}^m:ten}=1.0$ | $k^{\text{cat}}_{z_{10}^m:ten}=20.0$ | f |
| (X, VIIIa:IXa$^*$) | $Z_{10}^m$, $TEN^*$ | $Z_{10}^m : TEN^*$ | $E_{10}^m$ | $k^+_{z_{10}^m:ten}=1.31 \cdot 10^8$ | $k^-_{z_{10}^m:ten}=1.0$ | $k^{\text{cat}}_{z_{10}^m:ten}=20.0$ | f |
| (II, Va:Xa) | $Z_2^m$, $PRO$ | $Z_2^m : PRO$ | $E_2^m$ | $k^+_{z_2^m:pro}= 1.03 \cdot 10^8$ | $k^-_{z_2^m:pro}=1.0$ | $k^{\text{cat}}_{z_2^m:pro}=30.0$ | g |
| (XI-XI, IIa) | $Z_{11}^m$, $E_2^m$ | $Z_{11}^m : E_2^m$ | $E_{11}^{hm}$ | $k^+_{z_{11}^m:e_2^m} = 2.0 \cdot 10^7$ | $k^-_{z_{11}^m:e_2^m} = 1.0$ | $k^{\text{cat}}_{z_{11}^m:e_2^m} = 1.3 \cdot 10^{-4}$ | h |
| (IX, XIa) | $Z_9^m$, $E_{11}^{hm}$ | $Z_9^m : E_{11}^{hm}$ | $E_9$ | $k^+_{z_9^m:e_{11}^m} = 0.6 \cdot 10^7$ | $k^-_{z_9^m:e_{11}^m} = 1.0$ | $k^{\text{cat}}_{z_9^m:e_{11}^m} = 0.21$ | i |
| Binding (of -, with -) | | | | | | | |
| (VIIIa, IXa) | $E_8^m$, $E_9^m$ | | $TEN$ | $k^+_{\text{ten}}=1.0 \cdot 10^8$ | $k^-_{\text{ten}}=0.01$ | | e |
| (VIIIa, IXa$^*$) | $E_8^m$, $E_9^{m,*}$ | | $TEN^*$ | $k^+_{\text{ten}}=1.0 \cdot 10^8$ | $k^-_{\text{ten}}=0.01$ | | e |
| (Va, Xa) | $E_5^m$, $E_{10}^m$ | | $PRO$ | $k^+_{\text{pro}}=1.0 \cdot 10^8$ | $k^-_{\text{pro}}=0.01$ | | e |

**S11 Table. INHIBITION REACTIONS** (a) We estimate these parameters based on the half-lives of Factors IXa, Xa, IIa in plasma [34] and assume that the rate of fXIa inactivation is the same as that of fXa and thrombin. (b) For inhibition of fVa by APC, $k^{\text{cat}}_{e_5^m:APC} = 0.5$ sec$^{-1}$ and $K_M = 12.5 \cdot 10^{-9}$ [35]. We assume the same reaction rates for the inhibition of fVIIIa by APC. (c) From [36]. (d) $K_d = 0.5$ nM and $[PC] = 65$ nM [37]. (e) $k_{PC:TM:e_2^{ec}} = 0.167$ sec$^{-1}$ , $K_M = 0.7 \cdot 10^{-6}$ M [38].

| Reaction | Reactants | Product | M$^{-1}$sec$^{-1}$ | sec$^{-1}$ | sec$^{-1}$ | Note |
|---|---|---|---|---|---|---|
| Inactivation (of -, by -) | | | | | | |
| (IXa, AT-III) | $E_9$ | none | | $k^{in}_{AT:e_9}$=0.1 | | a |
| (Xa, AT-III) | $E_{10}$ | none | | $k^{in}_{AT:e_{10}}$=0.1 | | a |
| (IIa, AT-III) | $E_2$ | none | | $k^{in}_{AT:e_2}$=0.2 | | a |
| (XIa, AT-III) | $E_{11}$ | none | | $k^{in}_{AT:e_{11}}$=0.2 | | a |
| (APC, Va) | $APC,\ E_5^m$ | none | $k^{+}_{e_5^m:APC} = 1.2 \cdot 10^8$ | $k^{-}_{e_5^m:APC} = 1.0$ | $k^{\text{cat}}_{e_5^m:APC} = 0.5$ | b |
| (APC, VIIIa) | $APC,\ E_8^m$ | none | $k^{+}_{e_8^m:APC} = 1.2 \cdot 10^8$ | $k^{-}_{e_8^m:APC} = 1.0$ | $k^{\text{cat}}_{e_8^m:APC} = 0.5$ | b |
| Binding (of -, with -) | | | | | | |
| (TFPI, Xa) | $TFPI$,$E_{10}$ | $TFPIa$ | $k^{+}_{tfpia:e_{10}}$=$1.6 \cdot 10^7$ | $k^{-}_{tfpia:e_{10}}$=$3.3 \cdot 10^{-4}$ | | c |
| (TFPIa, TF:VIIa) | $TFPIa$,$E_7^m$ | $TFPIa : E_7^m$ | $k^{+}_{tfpia:e_7^m}$=$1.0 \cdot 10^7$ | $k^{-}_{tfpia:e_7^m}$=$1.1 \cdot 10^{-3}$ | | c |
| (TM, Thrombin) | $TM,\ E_2^{ec}$ | $TM : E_2^{ec}$ | $k^{\text{on}}_{TM} = 1.0 \cdot 10^8$ | $k^{\text{off}}_{TM} = 5.0 \cdot 10^{-2}$ | | d |
| Activation (of -, by -) | | | | | | |
| (PC, TM:$E_2^{ec}$) | $TM : E_2^{ec}$ | $APC$ | $k^{+}_{PC:TM:e_2^{ec}} = 1.7 \cdot 10^6$ | $k^{-}_{PC:TM:e_2^{ec}} = 1.0$ | $k^{\text{cat}}_{PC:TM:e_2^{ec}} = 0.16$ | e |

**S12 Table. PLATELET TRANSITIONS** (a) Estimated from data in [39, 40] as described in [1]. (b) Estimated from data in [41] as described in [1]. SE=subendothelium.

| Reactants | Reactants | Products | $M^{-1}sec^{-1}$ | $sec^{-1}$ | Note |
|---|---|---|---|---|---|
| Unactivated platelet adhering to SE | $PL$, SE | $PL_a^s$ | $k_{\text{adh}}^{+}=2 \cdot 10^{10}$ | $k_{\text{adh}}^{-}=0$ | a |
| Activated platelet adhering to SE | $PL_a^v$, SE | $PL_a^v$ | $k_{\text{adh}}^{+}=2 \cdot 10^{10}$ | $k_{\text{adh}}^{-}=0$ | a |
| Platelet activation by platelet in solution | $PL$, $PL_a^v$ | $2PL_a^v$ | $k_{plt}^{\text{act}} = 3 \cdot 10^8$ | | b |
| Platelet activation on SE | $PL$, $PL_a^s$ | $PL_a^v$, $PL_a^s$ | $k_{plt}^{\text{act}} = 3 \cdot 10^8$ | | b |
| Platelet activation by thrombin | $PL$, $E_2$ | $PL_a^v$ | | $k_{e_2}^{\text{act}}=0.50$ | b |

## References


1. Kuharsky AL, Fogelson AL. Surface-mediated Control of Blood Coagulation: The Role of Binding Site Densities and Platelet Deposition. Biophys J. 2001;80:1050–1074.

2. Fogelson AL, Tania N. Coagulation under flow: The influence of flow-mediated transport on the initiation and inhibition of coagulation. Pathophysiol Haemost Thromb. 2005;34:91–108.

3. Turitto VT, Leonard EF. Platelet adhesion to a spinning surface. Trans Amer Soc Artif Int Organs. 1972;18:348–54.

4. Young M, Carroad P, Bell R. Estimation of diffusion coefficients of proteins. Biotech and Bioeng. 1980;22(5):947–955.

5. Mann KG, Nesheim ME, Church WR, Haley P, Krishnaswamy S. Surface-dependent Reactions of the Vitamin K-dependent enzyme complexes. Blood. 1990;76:1–16.

6. Mann KG, Bovill EG, Krishnaswamy S. Surface-dependent reactions in the propagation phase of blood coagulation. Ann N Y Acad Sci. 1991;614:63–75.

7. Morrissey JH. Tissue Factor Modulation of Factor VIIa Activity: Use in Measuring Trace Levels of Factor VIIa in Plasma,. Thromb Haemost. 1995;74:185–188.

8. Novotny WF, Brown SG, Miletich JP, Rader DJ, Broze GJ. Plasma antigen levels of the lipoprotein-associated coagulation inhibitor in patient samples. Blood. 1991;78:387–93.

9. Weiss HJ. Platelet Physiology and Abnormalities of Platelet Function (Part 1). New Engl J Med. 1975;293:531–541.

10. Walsh PN. Platelet-Coagulant Protein Interactions. In: Colman RW, Hirsh J, Marder VJ, Salzman EW, editors. Hemostasis and Thrombosis: Basic Principles and Clinical Practice. 3rd ed. Philadelphia, PA: J.B. Lippincott Company; 1994. p. 629–651.

11. Nesheim ME, Pittman DD, Wang JH, Slonosky D, Giles AR, Kaufman RJ. The binding of S-labeled recombinant Factor VIII to activated and unactivated human platelets. J Biol Chem. 1988;263:16467.

12. Ahmad SS, Rawala-Sheikh R, Walsh PN. Comparative interactions of Factor IX and Factor IXa with human platelets. Journal of Biological Chemistry. 1989;264:3244–3251.

13. Mann KG, Krishnaswamy S, Lawson JH. Surface-dependent Hemostasis. Semin Hematol. 1992;29:213–26.

14. Baglia FA, Jameson BA, Walsh PN. Identification and Characterization of a Binding Site for Platelets in the Apple 3 domain of coagulation Factor XI. J Biol Chem. 1995;270:6734–40.

15. Miller TN, Sinha D, Baird TR, Walsh PN. A Catalytic Domain Exosite ($Cys^{527}$-$Cys^{542}$) in Factor XIa mediates binding to a site on activated platelets. Biochemistry. 2007;46:14450–60.

16. Tracy PB, Eide LL, Bowie EJ, Mann KG. Radioimmunoassay of factor V in human plasma and platelts. Blood. 1982;60:59–63.

17. Hubbell JA, McIntire LV. Platelet Active Concentration Profiles Near Growing Thrombi. A Mathematical Consideration. Biophys J. 1986;50:937–945.

18. Butenas S, Mann KG. Kinetics of Human Factor VII activation. Biochemistry. 1996;35:1904–1910.

19. Limentani SA, Furie BC, Furie B. The Biochemistry of Factor IX. In: Colman R, Hirsh J, Marder V, Salzman E, editors. Hemostasis and Thrombosis: Basic Principles and Clinical Practice. 3rd ed. Philadelphia, PA: J.B. Lippincott Company; 1994. p. 94–108.

20. Nemerson Y. The Tissue Factor Pathway of Blood Coagulation. Semin Hematol. 1992;29:170–176.

21. Monkovic DD, Tracy PB. Functional Characterization of Human Platelet-released Factor V and its Activation by Factor Xa and Thrombin,. J Biol Chem. 1990;265:17132–40.

22. Hill-Eubanks DC, Lollar P. von Willibrand factor is a cofactor for thrombin-catalyzed cleavage of the Factor VIII light chain. J Biol Chem. 1990;265:17854–8.

23. Lollar P, Knutson GJ, Fass DN. Activation of Porcine Factor VIII:C by Thrombin and Factor Xa. Biochemistry. 1985;24:8056–8064.

24. Gailani D, Broze Jr GJ. Factor XI activation in a revised model of blood coagulation. Science. 1991;253:909–12.

25. Gailani D, Ho D, Sun MF, Cheng Q, Walsh PN. Model for a Factor IX activation complex on blood platelets: dimeric conformation of Factor XIa is essential. Blood. 2001;97:3117–22.

26. Sinha D, Marcinkiewicz M, Navaneetham D, Walsh PN. Macromolecular substrate-binding exosite on both the heavy and light chains of Factor XIa mediate the formation of the Michaelis Complex required for Factor IX activaiton. Biochemistry. 2007;46:9830–9.

27. Krishnaswamy S, Jones KC, Mann KG. Prothrombinase Complex Assembly. Kinetic Mechanism of Enzyme Assembly on Phospholipid Vesicles. J Biol Chem. 1988;263:3823–3834.

28. Mann KG. Prothrombin and thrombin. In: Colman R, Hirsh J, Marder V, Salzman E, editors. Hemostasis and Thrombosis: Basic Principles and Clinical Practice. 3rd ed. Philadelphia, PA: J.B. Lippincott Company; 1994. p. 184–199.

29. Greengard JS, Heeb MJ, Ersdal E, Walsh PN, Griffin JH. Binding of coagulation Factor XI to washed human platelets. Biochemistry. 1986;25:3884–90.

30. Monkovic DD, Tracy PB. Activation of human Factor V by Factor Xa and thrombin. Biochemistry. 1990;29:1118.

31. Mann KG. The assembly of blood clotting complexes on membranes. TIBS. 1987;12:229–233.

32. Rawala-Sheikh R, Ahmad SS, Ashby B, Walsh PN. Kinetics of coagulation Factor X activation by platelet-bound Factor IXa. Biochemistry. 1990;29:2606–11.

33. Nesheim ME, Tracy RP, Tracy PB, Boskovic DS, Mann KG. Mathematical Simulation of Prothrominase. Methods Enzymol. 1992;215:316–328.

34. Rosenberg RD, Bauer KA. The Heparin-antithrombin System: A Natural Anticoagulant Mechanism. In: Colman RW, Hirsh J, Marder VJ, Salzman EW, editors. Hemostasis and Thrombosis: Basic Principles and Clinical Practice. 3rd ed. Philadelphia, PA: J.B. Lippincott Company; 1994. p. 837–860.

35. Solymoss S, Tucker MM, Tracy PB. Kinetics of inactivation of membrane-bound Factor Va by activated protein C. Protein S modulates Factor Xa protection. J Biol Chem. 1988;263:14884–90.

36. Jesty J, Wun TC, Lorenz A. Kinetics of the Inhibition of Factor Xa and the Tissue Factor-Factor VIIa Complex by the Tissue Factor Pathway Inhibitor in the Presence and Absence of Heparin. Biochemistry. 1994;33:12686–12694.

37. Griffin JH. Control of Coagulation Reactions. In: Beutler E, Coller B, Lichtman M, Kipps T, Seligsohn U, editors. Williams Hematology. New York: McGraw Hill; 2001. p. 1435–1447.

38. Broze GJ, Miletich JP. Biochemistry and Physiology of Protein C, Protein S, and Thrombomodulin. In: Colman RW, Hirsh J, Marder VJ, Salzman EW, editors. Hemostasis and Thrombosis: Basic Principles and Clinical Practice. Philadelpha: Lippincott Company; 1994. p. 259–276.

39. Turitto VT, Baumgartner HR. Platelet Interaction with Subendothelium in Flowing Rabbit Blood: Effect of Blood Shear Rate. Microvasc Res. 1979;17:38–54.

40. Turitto VT, Weiss HJ, Baumgartner HR. The Effect of Shear Rate on Platelet Interaction with Subendothelium Exposed to Citrated Human Blood. Microvasc Res. 1980;19:352–365.

41. Gear ARL. Platelet adhesion, shape change, and aggregation: rapid initiation and signal transduction events. Can J Physiol Pharmacol. 1994;72:285–94.
